# Structures of vertebrate R2 retrotransposon complexes during target-primed reverse transcription and after second-strand nicking

**DOI:** 10.1126/sciadv.adu5533

**Published:** 2025-06-20

**Authors:** Akanksha Thawani, Anthony Rodríguez-Vargas, Briana Van Treeck, Nozhat T. Hassan, David L. Adelson, Eva Nogales, Kathleen Collins

**Affiliations:** ^1^ California Institute for Quantitative Biosciences (QB3), Berkeley, CA, USA.; ^2^ Department of Molecular and Cell Biology, University of California, Berkeley, Berkeley, CA, USA.; ^3^ School of Biological Sciences, University of Adelaide, Adelaide, Australia.; ^4^ South Australian Museum, Adelaide, Australia.; ^5^ Howard Hughes Medical Institute, Chevy Chase, MD 20815, USA.; ^6^ Molecular Biophysics and Integrated Bioimaging Division, Lawrence Berkeley National Laboratory, Berkeley, CA, USA.

## Abstract

R2 retrotransposons are site-specific eukaryotic non–long terminal repeat retrotransposons that copy and paste into gene loci encoding ribosomal RNAs. Recently, we demonstrated that avian A-clade R2 proteins achieve efficient and precise insertion of transgenes into their native safe-harbor loci in human cells. The features of A-clade R2 proteins that support gene insertion are not well characterized. Here, we report high-resolution cryo–electron microscopy structures of two vertebrate A-clade R2 proteins at the initiation of target-primed reverse transcription and after cDNA synthesis and second-strand nicking. Using biochemical and cellular assays, we illuminate the basis for high selectivity of template use and unique roles for each of the three zinc-finger domains in nucleic acid recognition. Reverse transcriptase active site architecture is reinforced by an unanticipated insertion motif specific to vertebrate A-clade R2 proteins. Our work provides the first insights into A-clade R2 protein structure during gene insertion and may enable future improvement and adaptation of R2-based systems for precise transgene insertion.

## Teaser

Cryo-EM and functional analyses reveal features of vertebrate R2 retrotransposon proteins that support transgene insertion.

## INTRODUCTION

Non–long terminal repeat (non-LTR) retrotransposons are mobile genetic elements that are widespread in eukaryotic species. Retrotransposon-derived gene expression, mobilization, and rearrangement are recognized as major drivers of genome evolution and expansion (*1*–*3*). In mammals, retrotransposons have expanded via a copy-and-paste mechanism to compose a large portion of genomes. For example, nearly one-third of the human genome originated from the copy-and-paste activity of the reverse transcriptase (RT) encoded by the non-LTR retrotransposon, long interspersed element 1 (LINE-1) (*4*), which inserts within a degenerate DNA sequence stimulated in vitro by an adjacent single-stranded DNA gap (*5*–*7*). The abundant cDNA-derived genome content shapes nuclear organization, chromatin landscape, and transcription of genes and regulatory RNAs (*3*, *8*–*11*).

**Fig. 1.**
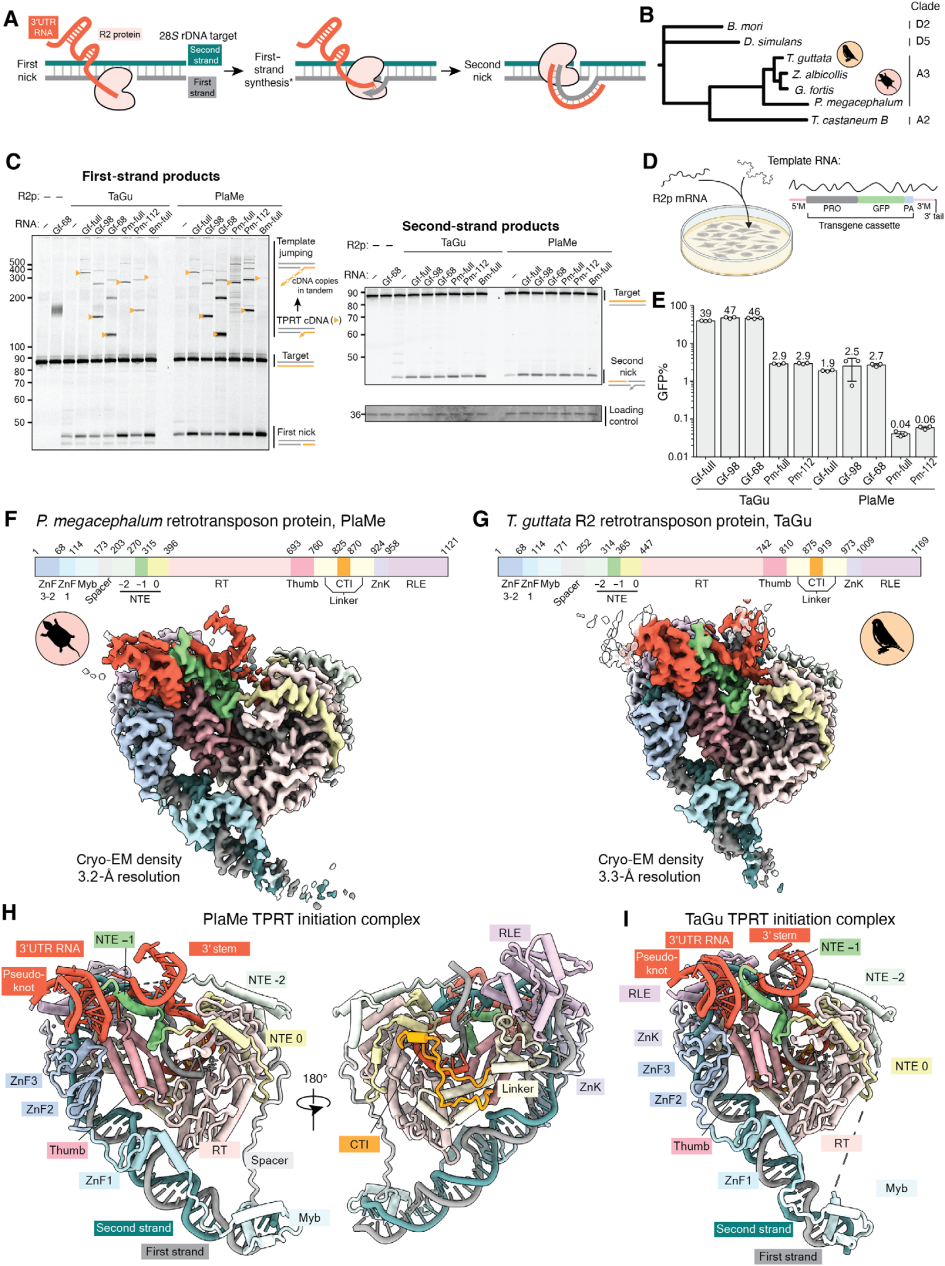
Activities and cryo-EM structures of A-clade R2 RNPs initiating TPRT. (**A**) Schematic of biochemical steps during DNA insertion. The second step marked with an asterisk (*) depicts the TPRT initiation state visualized in (F) and (G), and the final state depicts second-strand–nicked complex resolved in Fig. 5. (**B**) Phylogenetic analysis of R2 RT core (NTE −2 to CTI) from the A-clade (birds, turtle, and beetle) and D-clade (silk moth and fruit fly). (**C**) Denaturing PAGE with TPRT reaction products. 3′UTR RNA used were either a full-length 3′UTR RNA (Gf-full, Pm-full, and Bm-full) or their truncated versions (Gf-98, Gf-68, and Pm-112), each terminating in 5-nt homology to primer strand (R5). Orange triangles indicate expected TPRT product lengths for copying a single full-length template (TPRT cDNA). Multiple template RNAs may be copied in series (template jumping products). Regions of the same gel are imaged separately using different 5′ dyes. Loading control was detected with SYBR Gold. This is consistent for all TPRT gels hereafter. (**D**) PRINT assay schematic: mRNA encoding an R2p is transfected with an engineered template RNA comprising a 5′ module (5′M), modified CMV promoter (PRO), GFP ORF, polyadenylation signal (PA), and 3′ module (3′M) containing the 3′UTR or its truncation with terminal R4A22. Created with BioRender (*45*). (**E**) PRINT assays with PlaMe or TaGu mRNA and template RNA indicated. Data presented are mean values ± error bars indicating SD for three technical replicates. Note the log-scale *y* axis, which is consistent for PRINT assays. (**F** and **G**) Top: Colored domain schematics of PlaMe and TaGu with amino acid numbering (abbreviations given in the text). Bottom: Cryo-EM density maps of TPRT initiation complexes assembled with rDNA target site and either Gf-full (F) or Gf-98 RNA (G), colored by domains. (**H** and **I**) Ribbon diagrams of TPRT initiation complexes colored by domains.

Other non-LTR retrotransposons are more target site selective (*12*, *13*). R2 retrotransposons are sequence-specific mobile elements that insert to the tandemly repeated 28*S* ribosomal RNA (rRNA) precursor gene locus (the rDNA) and are found within the genomes of multicellular animals including insects, crustaceans, and nonmammalian vertebrates (*14*, *15*). R2 protein (R2p) from a moth *Bombyx mori*, hereafter BoMo, has long been the model system for biochemical characterization of target-primed reverse transcription (TPRT). R2p inserts a cDNA for the mRNA transcribed from the R2 element by nicking one of the two DNA strands of the target site to create a primer for cDNA synthesis directly into the genome (Fig. 1A) (*16*, *17*). R2p-mediated TPRT was recently repurposed to insert transgenes into rDNA loci in cultured human cells (*18*–*21*). This technology, called precise RNA-mediated insertion of transgenes (PRINT), relies on an avian R2p translated from an engineered mRNA co-delivered with a second RNA that templates transgene synthesis (*18*).

The avian R2 retrotransposons belong to the A-clade, which, among other clade-distinguishing differences, has an expanded number of N-terminal zinc-finger domains (ZnFs) compared to D-clade BoMo (*14*). Recent structural studies have revealed the architecture of BoMo ribonucleoprotein (RNP) bound to duplex DNA and the RNA from *B. mori* R2 3′ untranslated region (3′UTR) launched into TPRT (*22*, *23*). In comparison, A-clade R2p remains less well characterized biochemically and structurally. In particular, the role of the expanded array of ZnFs has not been elucidated, other than its significance for generating a more precise rDNA location of transgene 5′ junction formation with PRINT (*21*). Besides the N-terminal ZnFs, additional sequence differences distinguish A-clade from D-clade R2s, arising with the specialization of these two lineages in early multicellular animals (*14*, *24*). Understanding the structural features and biochemical properties of A-clade vertebrate R2ps will enable understanding of the underpinnings of their evolutionary specialization. Further, while the initial stage of TPRT has recently been characterized using BoMo (*22*), subsequent stages, such as when cDNA synthesis has completed and second-strand nicking occurs, remain unvisualized.

In this work, using cryo–electron microscopy (cryo-EM), we determine structures of an A-clade avian R2p that has optimal transgene insertion activity in human cell PRINT (R2p from the zebrafinch *Taeniopygia guttata*) as well as a newly isolated testudine (turtle) R2p that is in the Reptilia phylogenetic branch proximal to Aves. We resolve structures for each R2p RNP initiating TPRT as well as a structure after completion of cDNA synthesis and the second-strand nicking necessary for stable gene insertion (Fig. 1A). We also investigate the functional significance of A-clade-specific R2p structural features using biochemical and cellular assays.

## RESULTS

### TPRT activity of avian and testudine R2ps

Our work recently demonstrated that two avian R2p, from either zebrafinch (TaGu) or white-throated sparrow (*Zonotrichia albicollis*, ZoAl), support the PRINT protocol for RNA-templated transgene insertion into the native R2 rDNA target site in human cells (*18*). We also previously identified which of the 3′UTR sequences among closely related finch and sparrow R2s was optimal as a template RNA 3′ end, resulting in the choice of R2 3′UTR from the medium ground finch *Geospiza fortis* [*Gf* 3′UTR, henceforth Gf-full; 292 nucleotides (nt)] (*18*). For comparison to the avian R2s used in PRINT, we identified R2s outside of Aves but within the Reptilia phylogenetic group that gave rise to Aves (Fig. 1B). We chose R2p from the big-headed turtle *Platysternon megacephalum* (PlaMe) for additional study based on its robust biochemical activities as purified recombinant protein (described below). Upon comparing predicted structures of R2 3′UTRs across avian and testudine R2s, despite primary sequence divergence, we discovered a shared possible pseudoknot-hinge-stem architecture at the 3′ end of their 3′UTRs (fig. S1, A to C). The optimal template RNA Gf-full motif has this predicted structure (fig. S1, B and C) whereas a similar pseudoknot is not predicted or experimentally observed in *Bm* R2 RNA (fig. S1, A to C).

We assayed TPRT by TaGu and PlaMe using various 3′UTR RNAs: the initial avian Gf-full (*18*, *20*) and two Gf-full 5′-truncations containing the pseudoknot-hinge-stem motif (Gf-98 containing the 3′-terminal 98 nt and Gf-68 containing the 3′-terminal 68 nt; fig. S1, A to C) that also support PRINT (*25*), as well as the 210-nt full-length *Pm* R2 3′UTR (Pm-full) or 3′-truncated Pm-112 containing a predicted pseudoknot-hinge-stem motif (fig. S1A). In parallel, we assayed BoMo using the 248-nt full-length *Bm* 3′UTR (Bm-full), because the determinants of R2p binding and TPRT stimulation are distributed across the 3′UTR (*22*, *23*, *26*). Each 3′UTR sequence was followed by 5 nt of downstream rRNA (R5) that can base pair with primer created by the first-strand nick. TaGu and PlaMe used the A-clade 3′UTRs Gf-full, Pm-full, and each of their 5′-truncated versions for TPRT in vitro, but not the D-clade 3′UTR Bm-full (Fig. 1C), consistent with previous assays of avian R2p (*18*, *20*). After initial cDNA synthesis, R2p can bind excess free RNA in the TPRT reaction to extend the initial cDNA by template jumping (*26*, *27*), generating products larger than the initial cDNA (Fig. 1C, initial cDNA product is annotated with mustard arrowhead). Although all three R2ps initiated TPRT robustly, only A-clade R2ps nicked the second strand efficiently (fig. S2A). BoMo had low second-strand nicking activity under standard TPRT conditions, in agreement with previous studies (*23*).

### PRINT activity of avian and testudine R2ps

To compare A-clade R2p efficiency of transgene insertion to the TPRT target site in cells, we performed PRINT with template RNAs that encode an autonomous green fluorescent protein (GFP) expression cassette comprising a modified cytomegalovirus (CMV) promoter, GFP open reading frame (ORF), and polyadenylation signal (Fig. 1D). Template RNAs have a 5′ module for RNA biostability and a 3′ module with R2 3′UTR sequence followed by primer-complementary R4 and 22 adenosines (A22) optimal for PRINT (*18*, *20*, *25*). GFP-transgene template RNAs with a 3′ module containing Gf-full, Pm-full, or their truncated versions were delivered to human RPE-1 cells paired with an mRNA encoding TaGu or PlaMe (Fig. 1, D and E). Template RNA transfected in combination with mRNA encoding an RT-dead protein gave only background GFP signal (fig. S2B). TaGu paired with Gf-full, Gf-98, or Gf-68 template RNA generated between 39 and 47% GFP-positive (GFP^+^) cells, whereas, with Pm-full or Pm-112 template RNA, only ~2% of cells were GFP^+^. Although PlaMe paired with Gf-full, Gf-98, or Gf-68 template RNA generated about 2% GFP^+^ cells, PlaMe paired with Pm-full or Pm-112 template RNAs had much lower PRINT efficiency (Fig. 1E and fig. S2B). We conclude that despite robust biochemical activities in vitro, PlaMe has lower transgene insertion efficiency than TaGu. This is not necessarily unexpected because PRINT efficiency relies on many more activities than TPRT, such as nuclear entry of R2 RNP and nucleolar localization. However, that both A-clade R2p preferred the avian 3’UTR motif was unexpected. We suggest that this outcome reflects a more homogeneous folding of Gf-full and its truncations compared to Pm-full and its truncation, which is critical for PRINT in cells more than for TPRT in vitro.

### Structures of TaGu and PlaMe RNPs during TPRT initiation

**Fig. 2.**
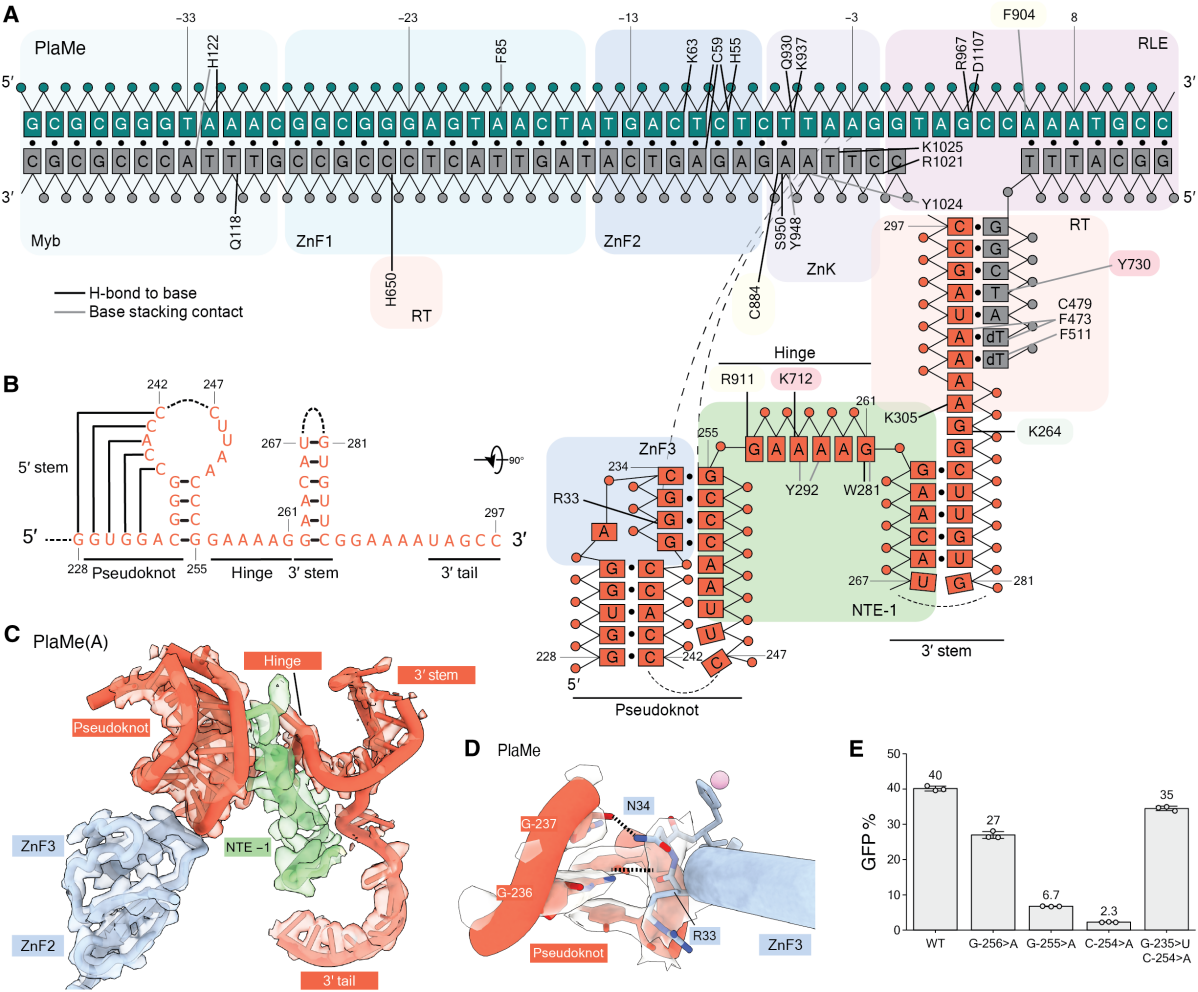
Protein and DNA recognition of R2 3′UTR RNA. (**A**) Schematic of direct interactions between PlaMe protein, rDNA target site, and 3′UTR RNA in the TPRT initiation complex (see fig. S6A for TaGu). Color scheme is consistent with Fig. 1. Solid black lines denote sequence-specific hydrogen bonds between protein residues and (deoxy)ribonucleobases, while dashed black lines represent hydrogen bonds between target site DNA and RNA bases. Solid gray lines denote pi-stacking contacts with (deoxy)ribonucleobases. Black circles represent base pairs. DNA numbering (green and gray strands) is negative upstream or positive downstream of the first-strand nick. RNA numbering (red strand) is from the start of Gf-full, as annotated in (A). (**B**) Secondary structure of the *Gf* 3′UTR RNA portions resolved in the TPRT initiation complexes for PlaMe and TaGu. Unresolved portions of the RNA are represented with dashed lines. (**C**) The 3′UTR RNA is engaged by the NTE −1 and ZnF3 domains (here, PlaMe; see fig. S6D for TaGu). ZnF3 domain from PlaMe contacts the pseudoknot of 3′UTR RNA. The cryo-EM map is shown in transparency. (**D**) Side chains in PlaMe ZnF3 make base-specific hydrogen bonds with G-236. PlaMe ZnF3 also makes a contact with the phosphate backbone of base G-237 at the junction of hinge and pseudoknot. The transparent densities correspond to the cryo-EM map around critical residues. Here and in subsequent figure panels, heteroatom representation has oxygen in red and nitrogen in blue. (**E**) PRINT assays using mRNA encoding TaGu and template RNA with 3′ module Gf-98 or a variant Gf-98 with R4A22 3′ tail. Base substitutions are numbered according to their position in Gf-full, with specific mutations described in the main text.

We sought to capture cryo-EM structures of A-clade R2p RNPs during TPRT using RNA templates that are optimal for efficient transgene insertion in cells. We therefore assembled TPRT complexes with PlaMe and TaGu proteins with either Gf-full or Gf-98 RNA. We incubated the proteins with biotinylated rDNA target site duplex (fig. S3, A and B), halted elongation after 1 nt of cDNA synthesis with dideoxythymidine triphosphate (ddTTP), and isolated complexes using a streptavidin-based pull-down strategy (fig. S3B). All intended components of ternary complexes were present in the eluted samples, and both proteins had nicked the first strand and initiated cDNA synthesis (fig. S3, C and D). Cryo-EM structure determination for PlaMe with Gf-full RNA in TPRT initiation stage reached an overall resolution of 3.2 Å (Fig. 1F and figs. S3E, S4A, and S5, A to C). While initial attempts to determine high-resolution cryo-EM structure of TaGu with Gf-full RNA did not succeed due to lower particle density, the particle density improved when we use the truncated Gf-98 RNA (fig. S3F), and we were able to obtain a structure of TaGu RNP in the TPRT initiation stage at an overall resolution of 3.3 Å (Fig. 1G and figs. S4B and S5, A to C). The cryo-EM density maps allowed us to model nearly the entire protein chain for PlaMe and TaGu as well as the upstream and downstream rDNA and an RNA pseudoknot-hinge-stem fold that forms an extensive surface for protein interaction (Figs. 1, H to I, and 2A, and fig. S6A). We also resolved density for ddTTP bound in the active site that is unable to join the cDNA 3′ end due to the incorporated ddTTP (fig. S6B).

The overall architectures of the A-clade R2p ternary complexes have both similarities and differences with the D-clade BoMo ternary complex captured at a similar stage of cDNA synthesis (fig. S6C) (*22*). The shared R2p core domains include the RT fingers and palm motifs (colored as RT domain) followed by the Thumb, a Linker, the C-terminal zinc-knuckle (ZnK), and the restriction-like endonuclease domain (RLE) (Fig. 1, F to I). As shown for D-clade BoMo, the A-clade R2p’s ZnK and RLE domains melt double-stranded DNA into single-stranded DNA across the first-strand nick site. Instead of the two N-terminal extension regions (NTE 0 and −1) observed in the BoMo structures (*22*, *23*), the A-clade R2p RT core is preceded by three segments of NTE: the two previously recognized (NTE 0 and −1) and a third (NTE −2) that was not described in the TPRT initiation complex of BoMo (*21*) or structures of bacterial retroelement proteins (*28*, *29*). NTE motifs are, in turn, preceded by an evolutionarily variable length of Spacer and the N-terminal ZnF and Myb domains (Fig. 1, F to I) that engage rDNA upstream of the first-strand nick. Large differences are present in the architecture of A-clade versus D-clade R2p interactions with RNA (see below) and in the unique two A-clade-specific R2p ZnF, which make contacts with DNA and RNA that have not been predicted from previous biochemical assays (*21*, *30*). Overall, our structures establish a divergence of A-clade and D-clade R2p nucleic acid interactions.

### RNA recognition by an N-terminal zinc finger and target DNA

Within Gf-full or Gf-98, only the RNA 3′-terminal 65 nt before the base-paired RNA-primer duplex is visible in our cryo-EM density maps (Fig. 1, F and H). The resolved regions of RNA correspond to the predicted pseudoknot and 3′ stem connected by a 6-nt hinge (Fig. 2, A to C, and fig. S1A). In addition to the provided primer-template duplex, an added ddTTP was also resolved. The fold and topology of RNA engaged with the A-clade R2ps differs from that of Bm-full engaged with BoMo, and more RNA length was resolved in the A-clade RNPs than was visible in BoMo RNP structures (*22*). The A-clade R2p NTE −1 and ZnF3-2 amino acid sequences form part of the large surface for RNA recognition (Fig. 2, A to C, and fig. S6, A and D). Sequence-specific recognition of the GAAAAG hinge sequence is mediated by residues within NTE −1, Thumb, and Linker regions (Fig. 2A and fig. S6A). These interactions likely contribute to the RNA template selectivity shared by avian and testudine R2p.

A-clade R2p N-terminal ZnF3 and ZnF2 fold together through a previously unanticipated interaction of β strands. This folding unit is sandwiched on target site DNA between ZnF1 and RLE and bookends the RNA pseudoknot from the side opposite NTE −1 (Fig. 2, A and C, and fig. S6, A and D). ZnF3 contacts the pseudoknot with hydrogen bonding interactions to both backbone and bases (Fig. 2D and fig. S6E). Our structures also reveal that the rDNA target site itself contributes to RNA recognition. We find that bases within the second-strand DNA region melted by R2p face toward the pseudoknot. In both PlaMe and TaGu structures, the base dA(−3) of the second strand creates a sequence-specific hydrogen bond with the base of G-255 at the junction between the pseudoknot and the hinge (fig. S6F). Analogous pairing of second-strand DNA and template RNA was observed in BoMo TPRT initiation (*22*).

To assay the functional significance of the visualized RNA secondary structure and its sequence, we made mutations in the pseudoknot and hinge regions and assessed change in PRINT efficiency in cells. Mutating the hinge base G-256 to A reduced PRINT efficiency and disrupting the pseudoknot base pairing via mutation of G-255 to A or C-254 to A markedly reduced PRINT efficiency (Fig. 2E). Restoring the pseudoknot base pairing with compensatory mutations (G-235>U, C-254>A) restored PRINT activity to a level comparable to PRINT with template RNA containing the wild-type pseudoknot sequence (Fig. 2E). Together, our structural and functional assays demonstrate that multiple regions of an A-clade R2p recognize and position template RNA predominantly via recognition of a tertiary structure 3′UTR RNA pseudoknot and hinge sequence.

### Target site recognition by R2 N-terminal DNA binding domains

**Fig. 3.**
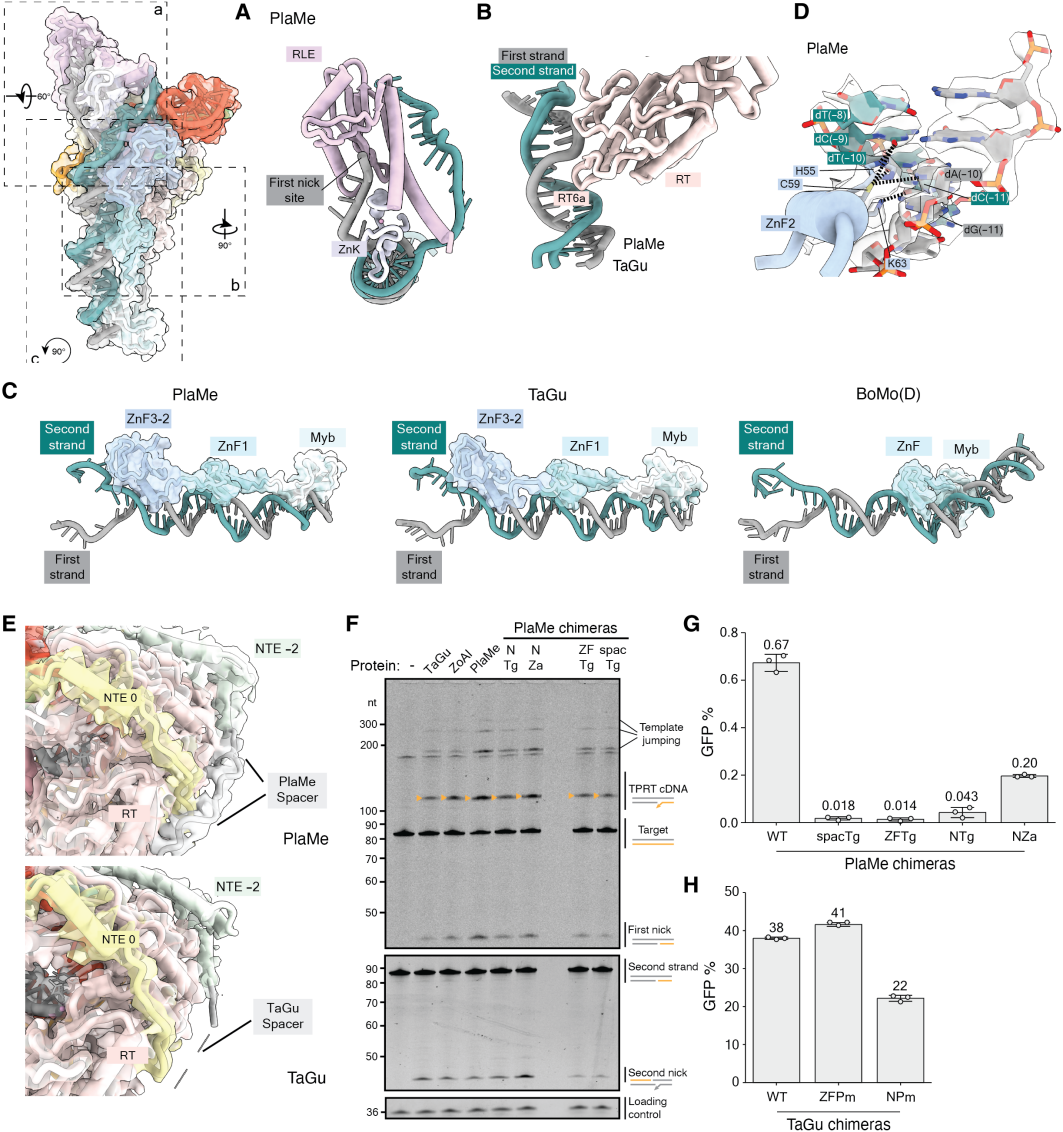
Protein recognition of the target site DNA and N-terminal R2p domain requirements. Top left: PlaMe TPRT initiation complex is represented with boxes marking regions highlighted in (A) to (C); relative rotational angles are indicated. Transparent density is the atomic surface. (**A**) RLE and ZnK domains surrounding the nicked first strand and single-stranded second strand for PlaMe. (**B**) RT motif 6a loop is shown protruding into the target site DNA. (**C**) Configuration on target site DNA of the N-terminal DNA binding domains: The three ZnFs and the Myb domain for A-clade PlaMe (left) and TaGu (center) are compared with the single ZnF and Myb in D-clade BoMo (right). Transparent density is the atomic surface. (**D**) Base-reading hydrogen bonds between ZnF2 and target site DNA proximal to the nick site. Transparent density depicts the cryo-EM map for critical protein residues and nucleotides involved in interactions. (**E**) Top: PlaMe Spacer and its interaction with the RT domain and NTE 0 motif. Bottom: Unresolved TaGu Spacer denoted by dashed lines. Atomic models are presented alongside the transparent unsharpened cryo-EM densities. (**F**) Denaturing PAGE of TPRT reaction products with wild-type TaGu, ZoAl, PlaMe, and chimeric proteins (~20 nM each): PlaMe with the N terminus (Spacer, Myb, and three ZnFs) from TaGu (NTg) or ZoAl (NZa), PlaMe with ZnF3-2 domains from TaGu (ZFTg), and PlaMe with Spacer from TaGu (spacTg). Gf-68 RNA with 5-nt primer homology (400 nM) and target site DNA (5 nM) were used for all reactions. (**G**) PRINT assays using mRNA encoding PlaMe or the chimeras described in (F). (**H**) PRINT assays using mRNA encoding TaGu or TaGu chimeras: TaGu with the N terminus (Spacer, Myb, and three ZnFs) from PlaMe (NPm), or TaGu with ZnF3-2 domains from PlaMe (ZFPm). For both (G) and (H), the template RNA 3′ module was Gf-full followed by R4A22.

As observed for BoMo (*22*), the A-clade R2p ZnK and RLE domains split double-stranded DNA around the first-strand nick site (Fig. 3A). The nicked strand upstream of the target site remains buried within the ZnK and RLE domains (Fig. 3A). As a second similarity with BoMo, the TaGu and PlaMe motif 6a within the RT domain wedges into a distortion of the upstream target site DNA (Fig. 3B), although the contacts and degree of DNA bending are not conserved (fig. S7A). As a major distinction, the three zinc fingers ZnF3-1 and the Myb domain of A-clade R2p create a more extended surface protecting the target site than occurs with D-clade BoMo, with the entirety of the four domains and connecting amino acid segments between them forming DNA contacts (Fig. 3C, summarized in Fig. 2A and fig. S6A). In comparison, BoMo ZnF and Myb domains occupy a much smaller surface of upstream target site (Fig. 3C). A-clade R2p ZnF3-2 engages the target site close to the first-strand nick site (Figs. 3C and 2A and fig. S6A). ZnF2 makes sequence-specific contacts (Fig. 3D), whereas ZnF1 and ZnF3 predominantly make sequence nonspecific contacts with the phosphate backbone of the target DNA (fig. S7, B to D). In contrast, BoMo ZnF, which corresponds to the A-clade R2p ZnF1, makes sequence-specific contacts (*22*, *23*).

In previous work using the ZoAl avian R2p with PRINT efficiency equal to TaGu (*21*), we found that deletion of ZnF3-2 had minimal impact on TPRT in vitro but reduced PRINT efficiency (*21*). The most marked consequence of ZnF3-2 removal was the decreased positional precision of transgene 5′ junction formation from the rDNA side (*21*). The sequence-specific contact between ZnF2 and upstream rDNA target site could be retained throughout cDNA synthesis (discussed in more detail below) and thereby influence DNA accessibility or positioning for the second-strand nicking necessary to generate the boundary between upstream rDNA and transgene 5′ end. In concurrence with this idea, our previous results showed that deletion of ZnF3-2 affects second-strand nicking in biochemical assays under some conditions (*21*). Overall, we show that the three A-clade R2p ZnFs have entirely different nucleic acid recognition principles compared against each other and to D-clade BoMo ZnF, which were not predicted by previous biochemical or structural assays.

A major difference between the PlaMe and TaGu proteins, in comparison to each other, is the disposition of the Spacer, the region that connects the N-terminal DNA binding domains to the NTE motifs (Fig. 1, H and I). The Spacer sequences and length are variable within A- and D-clade R2s (fig. S7E). TaGu has a Spacer of ~80 amino acids that could not be resolved in our cryo-EM map, whereas PlaMe has a Spacer of only ~30 amino acids that we partially observe in our structure in contact with the RT core (Fig. 3E). To investigate whether the difference in Spacer length or the N-terminal DNA binding domains gives PlaMe lower PRINT efficiency than TaGu, we used human cells to express chimeric PlaMe proteins with segments swapped to have an avian R2p Spacer, ZnF3-2, or entire N-terminal region before the NTE motifs. Purified domain-chimera proteins had robust TPRT activity roughly comparable to wild-type PlaMe (Fig. 3F), but each of the domain-chimera proteins suffered a large loss of PRINT efficiency especially severe for the Spacer or ZnF3-2 replacement alone (Fig. 3G). PlaMe with the entire N terminus of TaGu had substantially lower PRINT efficiency than PlaMe with the entire N terminus of ZoAl, the other avian R2p that supports high PRINT efficiency but, nonetheless, remained compromised for PRINT relative to wild-type PlaMe (Fig. 3G). Conversely, the chimera of TaGu with ZnF3-2 from PlaMe suffered no loss of PRINT efficiency, and substitution of the entire N-terminal region also supported PRINT albeit at lower efficiency (Fig. 3H). Together, the PlaMe chimeric protein results could hint at a functional divergence of the N-terminal nucleic acid binding domains and Spacer within vertebrate R2 A-clade proteins to an extent that they are not necessarily exchangeable modules of R2p domain architecture, despite appearing so from structures at TPRT initiation. The TaGu RT-RLE core may be less limited in activity than PlaMe RT-RLE core by changes in nucleic acid handling by the N-terminal domains.

### Expansion of the C-terminal insertion in vertebrate A-clade R2ps

**Fig. 4.**
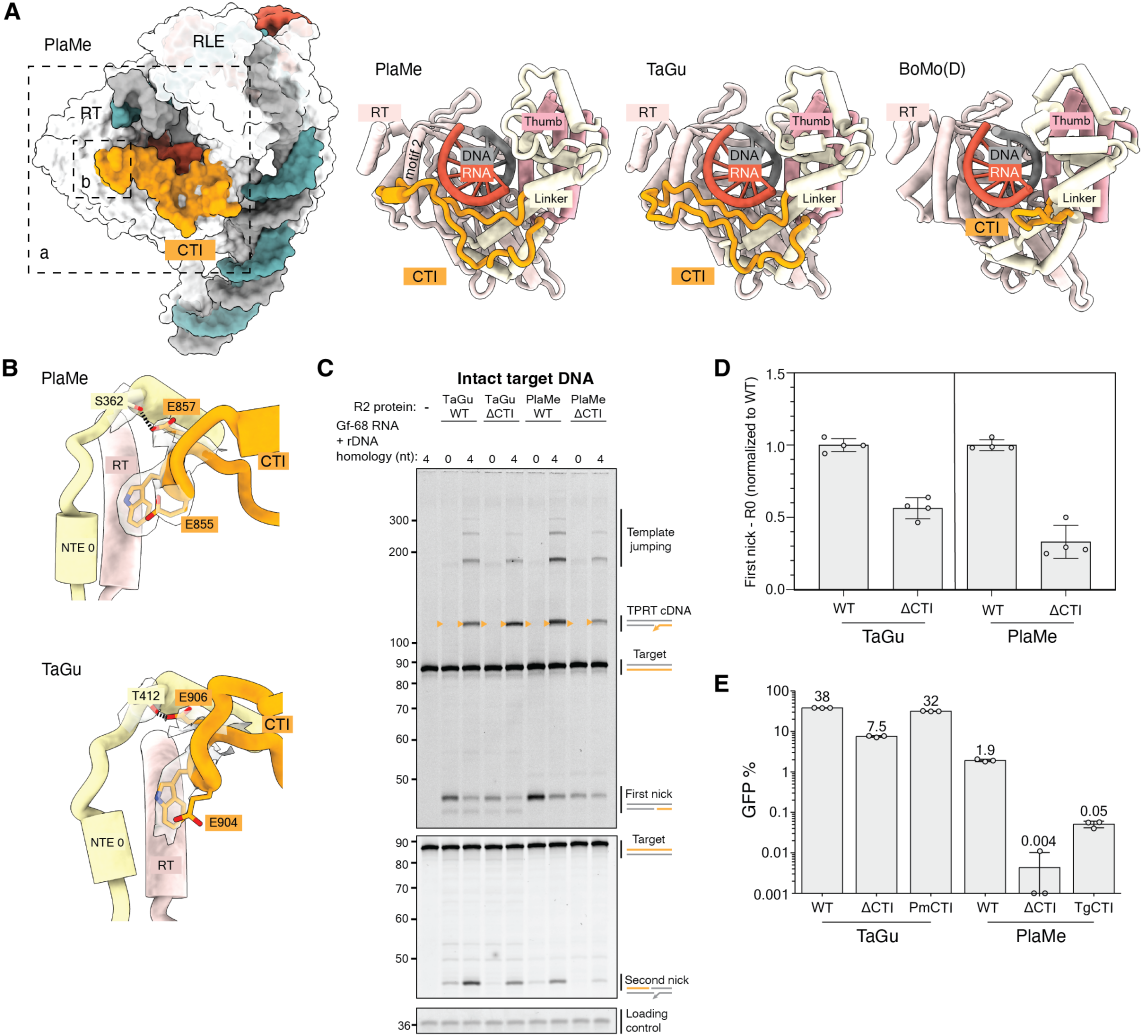
A C-terminal insertion in A-clade R2p. (**A**) The CTI is rendered in orange against the RT, Linker, and Thumb domains and RNA:cDNA duplex. The shorter CTI loop present in BoMo is shown for comparison (*22*, *23*). TaGu CTI protruding away from the RT domain and the central RNA:DNA duplex could only be traced using our unsharpened cryo-EM density map at low threshold. (**B**) Side chains of the conserved EWE motif that anchors the CTI to the RT domain and NTE 0 motif are displayed for PlaMe (top) and TaGu (bottom), where each dashed line represents a hydrogen bond. The transparent density corresponds to the cryo-EM map around those residues. (**C**) Denaturing PAGE of TPRT reaction products with proteins indicated and Gf-68 RNA with or without primer-complementary 3′ tail (4 or 0 nt). Orange triangles indicate expected TPRT product lengths for copying a single full-length template (TPRT cDNA). Multiple templates may also be copied in series (template jumping products). (**D**) Quantification of first strand nicking products in (C) when proteins are incubated with RNA lacking homology to target site DNA. Each replicate data point is shown, and bars are graphed as means ± 1 SD. (**E**) PRINT assays used the indicated R2p mRNA and template RNA with Gf-full followed by R4A22. Mean GFP % is indicated above each bar. WT, wild type.

A structural feature specific to the two vertebrate A-clade R2p is a sequence insertion following the RT and Thumb domains [hereafter C-terminal insertion (CTI)] that threads from the Thumb all the way to NTE 0 and RT motif 2 and then returns to the Thumb (fig. S8A and Fig. 4A). While this region in BoMo has 11 amino acids connecting two α helices (Fig. 4A), TaGu and PlaMe have a much longer 44 or 46 amino acids, respectively (fig. S8A). The CTI anchors to NTE 0 and RT domain within a glutamic acid–tryptophan–glutamic acid (EWE) amino acid triplet (Fig. 4B and fig. S8A) and, in PlaMe, has a short α helix not present in the TaGu CTI (Fig. 4, A and B). While the entire PlaMe CTI could be mapped in the cryo-EM density, the density for the part of the TaGu CTI away from the RT domain and the active site RNA:DNA duplex is only visible in our unsharpened cryo-EM map displayed with a low density threshold.

To investigate the functional significance of the longer CTI in avian and testudine A-clade R2p, we made partial truncations of the CTI in TaGu and PlaMe to match the length of this region in D-clade BoMo (ΔCTI mutants), deleting the intramolecular EWE anchor but not changing the fold of adjacent regions (fig. S8B) when modeled using AlphaFold3 (*31*). Wild-type and ΔCTI versions of TaGu and PlaMe were assayed for in vitro TPRT activity using Gf-68 RNA. Due to CTI positioning near the primer-template duplex, we investigated whether truncation of the CTI relaxed the requirement for template RNA 3′ tail base pairing to primer for TPRT initiation. CTI truncation did not markedly alter the amount of cDNA product synthesized using template RNA with R4 or template RNA lacking base pairing to primer DNA (Fig. 4C). Instead, unexpectedly, it decreased first-strand DNA nicking (Fig. 4C), which was especially evident in reactions with the template RNA that did not support TPRT (quantified in Fig. 4D). We also compared second-strand nicking activity using target-site DNA with the first-strand “pre-nicked” to obviate differences in first-strand nicking. Both the wild-type and ΔCTI mutant R2ps used pre-nicked rDNA for TPRT, but CTI truncation reduced second-strand nicking (fig. S8, C and D).

In contrast to the modest alteration of reconstituted TPRT, CTI truncation resulted in strong reduction of PRINT activity for both TaGu and PlaMe (Fig. 4E). To investigate whether this loss of efficiency could be due to reduced processivity of cDNA synthesis in cells, we used droplet-digital polymerase chain reaction (ddPCR) to quantify the percentage of full-length transgene insertions (*18*). A slight reduction in full-length transgene fraction was evident for ΔCTI TaGu (fig. S8F), but the overall reduction in PRINT efficiency upon CTI truncation is far more severe (compare Fig. 4E and fig. S8E). When we implanted PlaMe’s deleted CTI region into the TaGu protein with truncated CTI, or vice versa, PRINT efficiency recovered (Fig. 4E). These results reinforce the necessity of the CTI motif for PRINT. Together, we hypothesize that CTI expansion stabilized the R2p RT fold in a manner that enhances endonuclease activity and is more critical in cells than in reconstituted TPRT reactions. In a recent study (*19*), the TaGu CTI was inferred to be a disordered loop and, therefore, used as a location for insertion of accessory protein modules. In contrast, our R2p structure and the results from our functional assays recommend against CTI disruption in an avian A-clade R2p used for transgene insertion.

### Structure of PlaMe after cDNA synthesis and second-strand nicking

**Fig. 5.**
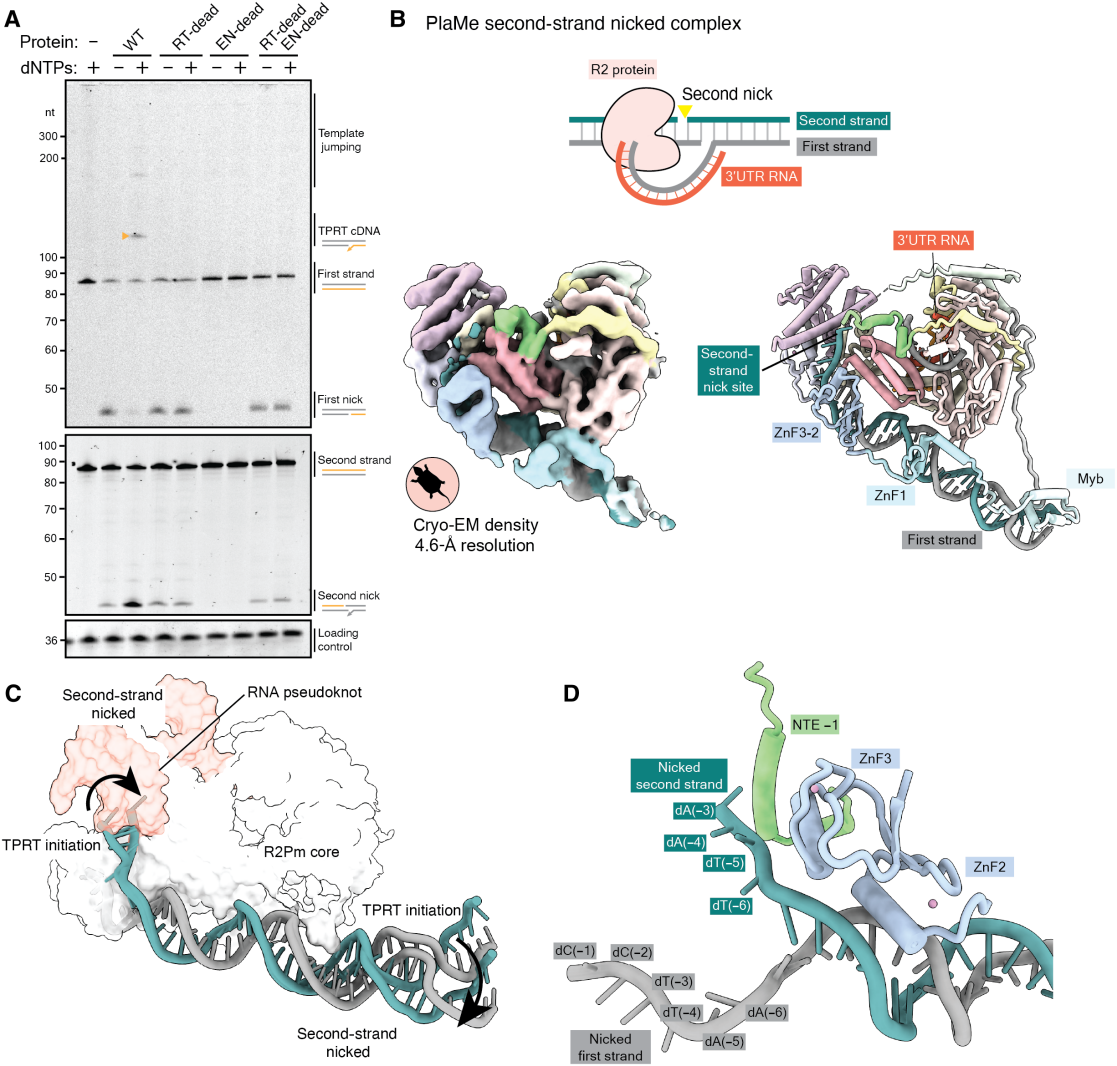
Biochemical activity and cryo-EM structure of A-clade R2p related to second-strand nicking. (**A**) Denaturing PAGE of target site DNA nicking and TPRT reaction products from assays using wild-type TaGu or its RT-dead and EN-dead variants. Gf-68 RNA with R5 was used as template RNA. Small triangle (mustard) indicates TPRT cDNA. (**B**) Top: Nucleic acid substrate design to capture a post-TPRT structure, accomplished using PlaMe. Bottom: Cryo-EM density (left) and ribbon diagram (right) of assembled PlaMe second-strand–nicked complex, colored by domains. (**C**) Comparison of upstream target site DNA position in the PlaMe TPRT initiation complex versus second-strand–nicked complex. The atomic surface of the PlaMe RT core is displayed in white and the 3′UTR RNA in red hue. (**D**) Nicked ends of upstream target site DNA are illustrated with nearby PlaMe protein regions NTE −1 and ZnF3-2.

Beyond TPRT initiation, biochemical and structural insights about the sequence of steps required for stable DNA insertion are scarce. For example, although second-strand nicking is required for second-strand synthesis, it is unknown whether that can occur with R2p still bound to the upstream target site, protecting it from nuclease and signaling machineries. First, to confirm that second-strand nicking required the R2p EN active site, we purified wild-type TaGu as well as RT and endonuclease active-site mutants (RT-dead and EN-dead, respectively). When combined with Gf-68 RNA and target site DNA, the wild-type and RT-dead proteins but not EN-dead proteins nicked the second strand (Fig. 5A). Second-strand nicking improved when the wild-type protein was able to perform first-strand cDNA synthesis upon addition of deoxynucleotide triphosphates (dNTPs; Fig. 5A). A mixture of EN-dead and RT-dead proteins gave the same level of second-strand nicking as RT-dead protein alone (Fig. 5A), consistent with a lack of cooperation between RT-dead and EN-dead proteins in PRINT (*18*). We conclude that RT-dead and EN-dead R2p are not rapidly exchanging on the target site.

For structure determination of an R2p stage after completion of first-strand synthesis, we assembled PlaMe and TaGu proteins with an annealed nucleic acid structure corresponding to near full-length cDNA synthesis on the Gf-68 template RNA (Fig. 5B and fig. S9A). The RNA had one unpaired 5′ nt that R2p should use to template addition of dideoxycytidine (ddCTP) to complete cDNA synthesis. Reactions including ddCTP were used to generate the intended R2p complexes. Following purification, we analyzed composition by denaturing polyacrylamide gel electrophoresis (PAGE; fig. S9, B and C). Some complexes had an intact second strand, but complexes with the second strand nicked were also evident (fig. S9, B and C). Cryo-EM sample preparation to visualize second-strand–nicked complexes was more successful for PlaMe samples, while TaGu samples suffered from low particle densities.

Our PlaMe cryo-EM analysis identified a particle population with a nicked second strand from which we were able to generate a structure of PlaMe after cDNA synthesis and second-strand nicking with an overall resolution of 4.6 Å (figs. S9D and S10 and Fig. 5B). The structure revealed that R2p is still bound to upstream target site with all three ZnFs and the Myb domain, as at the initiation of TPRT (fig. S11), with only a slight upstream target site DNA unbending (Fig. 5C). The major change in nucleic acid configuration was the lack of downstream target site DNA density. Only a minimal length RNA-DNA duplex appeared well-ordered, suggesting that most of the duplex cDNA product and contiguous downstream target site DNA are not constrained. The upstream target site second strand near the nick site was not well resolved in the cryo-EM density (Fig. 5C). Nevertheless, we could trace the single-stranded region of the upstream second strand toward the second nick site (Fig. 5D) through dA(−3), consistent with second-strand nicking 2 base pairs (bp) upstream from the first-strand nick (*17*, *21*). Unexpectedly, upon second-strand nicking, the 3′ end of the second strand moved into a position occupied by the RNA pseudoknot at the initiation of TPRT, closer to ZnF3-2 and NTE −1 (Fig. 5, C and D). We suggest that this positioning could enable R2p to protect the nicked second-strand 3′ end from inappropriate processing or joining until R2p is actively removed from the upstream target site by machinery that forms the insertion second-strand DNA 5′ junction.

## DISCUSSION

### Structural variations during R2 evolution

In this study, we investigated the structural underpinnings of TPRT progression for A-clade R2, a lineage that began diverging from D-clade R2 in early multicellular eukaryotic evolution. A-clade R2p is distinguished by its expanded array of N-terminal ZnFs (*13*, *15*) as well as other specializations visualized and functionally assayed in this work. We find that each of the three A-clade R2p ZnFs has a distinct nucleic acid recognition specificity, with ZnF3 having a network of interactions as it co-folds with ZnF2, binds target site DNA proximal to the nick sites, and anchors a template RNA tertiary structure through TPRT initiation. Gain of the ZnF3-2 module would have increased specificity of nucleic acid interaction at upstream target site DNA, likely precluding the R2p trade-off between binding upstream and downstream of the nick positions that has been investigated with two insect D-clade R2p (*21*, *32*). An interesting feature of vertebrate A-clade R2p revealed by our study is the CTI anchor, which is more critical for PRINT in cells than it is for TPRT in vitro. It will be of high interest to investigate CTI sequence and structure across a wider diversity of A-clade R2p and link its structural diversity to functional differences at biochemical and cellular levels.

Extending from our initial findings (*18*), here, we found that PlaMe has higher PRINT efficiency when combined with template RNA containing an avian R2 3′UTR versus the species-matched *Pm* R2 3′UTR. The *Gf* 3′UTR RNAs’ outperformance of *Pm* 3′UTR RNAs could reflect differences in native R2 features that contribute to suboptimal retrotransposition by the PlaMe protein in its native context. Alternately, the suboptimal function of *Pm* 3′UTR could be due to differences in folding of in vitro transcribed RNA transfected into human cells relative to RNA folding by RNA polymerase I expression in the native context. What 3′UTR RNA sequence, structure, and folding stability are optimal for retrotransposition in native R2 contexts could be investigated in future studies.

### Second-strand nicking and DNA repair

We visualized the first structure of R2p, or any non-LTR retrotransposon protein, at an intermediate stage of new gene insertion subsequent to TPRT-launched cDNA synthesis. The R2p complex that remains stable after second-strand nicking protects both the upstream target site and the cDNA 3′ end, which would bridge the target site upstream and downstream flanking sequences via protein. Second-strand nicking mechanism seems to differ between D-clade and A-clade R2p, with only A-clade R2p remaining bound to upstream DNA. Also, because the nicked 3′ end of the second strand is not located near the RT active site, it is unlikely that second-strand cDNA synthesis could be initiated by the initially bound R2p. Recruitment of DNA repair factors encoded by the host cell genome would be required to complete the insertion of a new, stable double-stranded DNA gene.

We note that, while R2p can make an appropriately positioned second-strand nick in vitro, whether R2p makes the second-strand nick in cells remains unresolved. We favor the hypothesis that R2p does make the second-strand nick, in part, because deletion of ZnF3-2 inhibits second-strand nicking under some conditions in vitro and strongly decreases the fidelity of 5′ junction formation for transgene insertion by PRINT (*20*). If R2p does second-strand nicking, whether this is mediated by the initially recruited R2p or by a second R2p acting in concert will be important to resolve. In comparison, BoMo second-strand nicking activity may be triggered by binding of an R2 RNA 5′ region potentially unique to *B. mori* R2 (*23*), which, in biochemical assays, favors a downstream binding site for the N-terminal ZnF and Myb domains (*32*). We have not found any similarly functional 5′ region within the ORFs of other R2 retrotransposons from A-clade or D-clade; however, more thorough analysis will be required to establish the evolutionary origin and biological function of a D-clade R2 5′ RNA motif.

### R2-derived transgene insertion tools

Recent work from our group and others demonstrates that an avian A-clade R2p can precisely deliver transgenes to rDNA loci in human cells, which, in the case of PRINT, was founded on the principle of RNA-only delivery (*18*, *19*). A next frontier in retrotransposon-based transgene insertion tools will be to develop a site-programmable transgene insertion technology that exploits site-specific TPRT. Besides creating a programmable Cas enzyme fusion with an R2p, similar to prime editor design (*33*), one possibility would be to replace or supplement the ZnF array with heterologous sequence-specific DNA binding domains, adopting a design principle from zinc-finger nucleases and transcription activator-like effector nucleases (*34*, *35*). However, our domain swap results indicate that the N-terminal DNA binding domains and adjacent Spacer are not modularly exchangeable even between structurally similar R2p. It will be critical to understand in more detail the structural and kinetic coordination of nucleic acid recognition, nicking, and cDNA synthesis. The structures described in this work inform the high specificity of native vertebrate A-clade R2p for both template RNA and target site DNA and provide the groundwork for future improvements and possible reprogramming of R2p-based transgene insertion to the human genome.

## MATERIALS AND METHODS

### Testudine R2 retrotransposon identification

BLASTN+ searches used avian R2 sequences as queries against testudine genome assemblies including *P. megacephalum* (sensitive search, word size of 7) (*36*). Top hits flanked by 28*S* rDNA were annotated as full-length and ORFs translated using ExPASY (*37*). The R2 used for this study was selected on the basis of ORF completeness and conservation of essential residues.

### Protein expression and purification

Construct sequences used in this work are provided in table S1. Codon-optimized R2 ORFs and other DNA modules were purchased from GenScript. R2 ORFs were cloned into a pET45b vector with N-terminal His^14^-MBP-bdSUMO tags and C-terminal TwinStrep for bacterial expression (Addgene, vector no. 176534). R2 plasmids were transformed into BL21(DE3) *Escherichia coli* and expressed in modified terrific broth medium with autoinduction as described previously (*22*) with modifications that included supplementing medium with 40 mM dipotassium hydrogen phosphate, 50 mM potassium dihydrogen phosphate, 1 mM magnesium sulfate, and 25 mM ammonium sulfate. Additionally, the lysis buffer for resuspension of bacterial cell pellets was supplemented with ribonuclease A (RNase A; Sigma-Aldrich, R6513, 15 μg/ml), 0.1% Igepal CA-630 (USB Corporation), lysozyme (0.5 mg/ml; Sigma-Aldrich), 0.2 mM phenylmethylsulfonyl fluoride (PMSF), and protease inhibitor cocktail (Sigma-Aldrich). Pelleted cells from a 1-liter *E. coli* culture were lysed with sonication, and the lysate was clarified by centrifugation at 30,000 rpm using a Ti45 rotor (Beckman Coulter) for 30 min.

For cryo-EM analysis of the TaGu TPRT initiation and PlaMe second-strand–nicked complexes, pelleted cells from 1 liter of *E. coli* cultures were lysed in binding buffer [25 mM Na-Hepes (pH 7.5), 1 M NaCl, 10% glycerol, 6 mM β-mercaptoethanol (BME), 0.1% NP-40 supplemented with 0.5 mM PMSF, protease inhibitor cocktail, and deoxyribonuclease I (DNase I)]. The proteins were purified with the Strep-Tactin Superflow Plus resin (QIAGEN) and eluted with buffer [25 mM Na-Hepes (pH 7.5), 500 mM NaCl, 10% glycerol, 6 mM BME, 0.01% NP-40] supplemented with 5 mM d-desthiobiotin. For cryo-EM analysis of PlaMe TPRT initiation complex, the bacterial cell pellet was lysed in the same lysis buffer with 40 mM Imidazole. This protein was purified with Ni-NTA resin (QIAGEN), followed by elution with elution buffer containing 300 mM imidazole. All eluates for cryo-EM analyses were subjected to further purification on a HiTrap Heparin HP 5-ml column (Cytiva) to remove contaminating nucleic acids. The heparin column was equilibrated with a buffer containing 20 mM Hepes-Na (pH 7.5), 300 mM NaCl, 10% glycerol, 6 mM BME, and 0.01% NP-40; peak fractions from the previous affinity step were loaded, and protein was eluted with a gradient of 300 mM to 2 M NaCl. Peak elution fractions were analyzed on SDS-PAGE, concentrated, flash frozen in liquid, and stored in −80°C. Protein concentrations were determined by analyzing with Bradford reagent (Bio-Rad) against a known bovine serum albumin standard.

For in vitro TPRT, we used predominantly bacterially expressed proteins purified with a single step of Strep-Tactin Superflow Plus resin (QIAGEN) contained in a gravity-flow column (Bio-Rad), which was washed and eluted following the resin manufacturers’ protocol and compatible buffers described previously (*22*) with modifications including supplementing storage buffer with 2 mM dithiothreitol. The N-terminal solubility tag was retained for in vitro assays because the presence or absence of the tag did not affect TPRT results (fig. S1E). The N-terminal domain chimera proteins were expressed in and isolated from human embryonic kidney (HEK) 293T cells to avert concerns that these chimeric proteins could have altered folding upon bacterial expression that was not relevant for PRINT assays in human cells. N-terminally 1xFLAG-tagged proteins were purified using FLAG antibody resin and determined for concentration as described previously (*17*, *35*). Proteins were flash frozen in liquid and stored in −80°C. SDS-PAGE gels were scanned using the scanner Epson Perfection V550 Photo (Epson America), and protein concentrations were determined by densitometry analysis of Coomassie blue–stained gels using ImageJ relative bovine serum albumin standard.

The protein mutations made in this study included internal truncations (∆CTI), double alanine substitutions (TaGu RT-dead and EN-dead), or protein segment swaps (PlaMe and TaGu chimeras). For full N terminus swaps, we swapped PlaMe protein residues Q1-G204 with M1-Q252 from TaGu or M1-G242 from ZoAl. For ZnF swap, ZnF3-2 within the PlaMe protein (Q1-P72) was substituted TaGu (M1-P70) or the opposite. For the swap of the Spacer region of PlaMe (L170-G204), the chimera had TaGu K171-Q252. For CTI partial truncations (∆CTI) in TaGu and PlaMe, we truncated positions P884-F914 and P833-Y865, respectively. The CTI exchange between TaGu and PlaMe replaced A876-N919 from TaGu with PlaMe A825-N870 or the converse). TaGu EN-dead is D1054A and D1067A, and RT-dead is D657A and D658A. Swaps and truncations were designed to minimize impact on protein fold as modeled using AlphaFold3.

### RNA transcription and purification

Nucleic acid sequences used in this study are provided in table S1. For in vitro assays, 3′UTR sequences of the vertebrate R2 retrotransposons were PCR amplified from parent vectors to include the T7 RNA polymerase promoter. All RNAs were transcribed with T7 RNA polymerase in 40 to 60 μl of reactions with HiScribe T7 High Yield RNA Synthesis Kit [New England Biolabs (NEB)] for 5 hours at 37°C. Template DNA was removed with DNase RQ1 (Promega), and RNA was separated on an 8 to 12% denaturing polyacrylamide gel. Full-length RNA was excised and eluted with RNA elution buffer [300 mM NaCl, 10 mM tris (pH 8), 0.5% SDS, and 5 mM EDTA] overnight at 4°C. The RNA was supplemented with 25 μg of glycogen, precipitated with 100% ethanol, centrifuged, washed with 70% ethanol, and air dried. When used for cryo-EM, RNA was supplemented with RiboLock (Thermo Fisher Scientific) before storage at −20°C. Integrity of purified RNA was verified by denaturing PAGE and SYBR Gold nucleic acid gel stain (Thermo Fisher Scientific) detected by scanning with Typhoon 5 (Cytiva). For PRINT assays, previous protocols were used (*18*, *25*).

### Preparation of TPRT DNA substrates for in vitro assays

Oligonucleotide duplex strands [Integrated DNA Technologies (IDT)] used in this study have a 3′ block to prevent cDNA synthesis without target site nicking (table S1). Target DNA for in vitro assays was an 84-bp duplex with both of its strands labeled on their 5′ ends with fluorescent dyes that had nonoverlapping emission spectra. For the first strand, the sequence is /5IRD800CWN/ATTCATGCGCGTCACTAATTAGATGACGAGGCATTTGGCTACCTTAAGAGAGTCATAGTTACTCCCGCCGTTTACCCGCGCTTG/3Phos/. The complementary second strand is /5Cy5/CAAGCGCGGGTAAACGGCGGGAGTAACTATGACTCTCTTAAGGTAGCCAAATGCCTCGTCATCTAATTAGTGACGCGCATGAAT/3Phos/. Before annealing, to improve purity and reduce background signal, we size selected and purified from denaturing PAGE each strand following the same approach as for extracting RNA (see the “RNA transcription and purification” section). To anneal these 84-nt strands, we first made 10× stocks of expected duplex DNA resuspended in 50 mM KCl and 1 mM MgCl_2_ before heating ssDNA to 95°C for 1 min and then gradually cooled to 25°C over 1 hour using a thermocycler. These annealed substrates were stored at −20°C until use. For all experiments, we used a final concentration of 12 nM duplex DNA, except for Fig. 5A, where concentration was reduced to 5 nM to minimize background signal that could obscure product detection.

### TPRT reactions

In vitro TPRT was performed as previously (*18*, *26*), with modifications. TPRT reactions were assembled on ice in a volume of 20 μl with final concentrations of 25 mM tris-HCl (pH 7.5), 150 mM KCl, 5 mM MgCl_2_, 10 mM dithiothreitol, 2% w/v PEG-6000, 12 nM target DNA duplex, 400 nM template RNA, 0.5 mM dNTPs, and 30 nM bacterially expressed protein. Protein was added last in the reactions. For Fig. 3F, we used instead 20 nM HEK293T protein and 5 nM target DNA. For Fig. 5A, one (30 nM) or two proteins (15 nM each) were added simultaneously as the last component in the reaction, and 5 nM rDNA target was used. Reactions were incubated at 30°C for 15 min before heat inactivation at 70°C for 5 min, followed by addition of 2 μl of RNase A (10 mg/ml), incubation for 15 min at 55°C, and dilution with 80 μl of stop solution [50 mM tris-HCl (pH 7.5), 20 mM EDTA, and 0.2% SDS] spiked with 5 to 20 ng of a loading control oligonucleotide (table S1). Product DNA was purified by phenol-chloroform-isoamyl alcohol (Thermo Fisher Scientific, BP17521-100) extraction and ethanol precipitation with 10 μg of glycogen as carrier with snap freezing with liquid nitrogen. Samples were pelleted at ~18,000*g* for 15 to 20 min at 4°C, and pellets were washed with 75% (v/v) ethanol and resuspended in 15 μl of 0.5× formamide loading dye [95% v/v deionized formamide, 0.025% w/v bromophenol blue, 0.025% w/v xylene cyanol, and 5 mM EDTA (pH 8.0)]. Samples were incubated at 95°C for 3 min and then placed on ice before loading half of the sample on a denaturing PAGE gel (9% acrylamide/bis 19:1, 7 M urea, and 0.6× tris-borate EDTA). Gel scans used a Typhoon 5 (Cytiva) for detection of fluorescent dyes. Size markers were detected by performing a subsequent gel scan after 6-min incubation with SYBR Gold stain (Thermo Fisher Scientific, S11494). ImageJ was used to analyze signal intensity of products indicated in Fig. 4C. Products of interest in Fig. 4C are quantified in Fig. 4D by subtracting background before normalizations relative to the wild-type versions of each protein. We similarly analyzed products of fig. S8C and quantified in fig. S8D. All graphical data represents means ± 1 SD for the indicated number of technical replicates.

### R2 RT phylogenetic tree, RNA, and protein sequence alignments

R2p sequences used in Fig. 1B and figs. S7E and S8A were collected from previous publications (*14*, *18*, *24*, *30*), except the identification of PlaMe described above. For R2ps without a cryo-EM structure, we used AlphaFold3 (*28*) to predict domain and motif boundaries. We used MAFFT v7.490 (auto model selection) (https://mafft.cbrc.jp/alignment/server/index.html) to align our amino acid sequences of interest. We then used IQTREE v1.6.11 (www.hiv.lanl.gov/content/sequence/IQTREE/iqtree.html) for tree reconstruction with 20 maximum likelihood trees and 1000 bootstraps (ModelFinder -m MFP). We used *B. mori* as the outgroup. The protein alignments in figs. S7E and S8A were generated using MAFFT (v7), and the RNA sequence alignment in fig. S1A was performed using Clustal Omega (www.ebi.ac.uk/jdispatcher/msa/clustalo).

### Cell culture

RPE-1 cells were grown in Dulbecco’s modified Eagle’s medium/F12 (Gibco) supplemented with 10% fetal bovine serum (FBS; Seradigm) and Primocin (100 μg/ml; InvivoGen). Cells were cultured at 37°C under 5% CO_2_. All cells were tested for mycoplasma contamination, and human cell lines were validated by short tandem repeat profiling (Promega, B9510).

### PRINT by two-RNA delivery

RPE-1 cells at 50% confluency, in log-phase growth, were replated at 350,000 cells per well in 12-well plates. Cells were reverse transfected with mRNA and template RNA using Lipofectamine MessengerMAX at ^1^/_2_ mass/volume ratio as per the manufacturer’s instructions. A total of 0.5 μg of RNA was transfected using an mRNA:template RNA molar ratio of 1:3. Cells were collected 20 to 24 hours after transfection. R2p mRNAs encoded C-terminally 3xFLAG-tagged proteins and had a plasmid-encoded A30 tract to reduce plasmid instability with the longer A-tract used in initial PRINT studies.

### Flow cytometry

Cells were trypsinized, and trypsin was inactivated by addition of Dulbecco’s Phosphate-Buffered Saline (dPBS; -Mg^2+^ and -Ca^2+^) supplemented with 0.5 mM EDTA and 2% FBS. Cell samples were then analyzed by Attune NxT Flow Cytometer (Thermo Fisher Scientific) under the voltage setting of forward scatter (FSC) at 70 V, side scatter (SSC) at 280 V, BL1 at 250 V. Data analysis was performed in FlowJo (v. 10.8.1). Cells transfected with template RNA only were used as negative controls. The %GFP^+^ was calculated by subtracting template-alone %GFP^+^. Three technical replicates were performed for each PRINT experiment, such that three different RPE-1 wells were used and displayed for each bar of bar graphs. All data within one bar graph was from transfections done in parallel with the same cells. All PRINT experiments described here were repeated with at least one biological replicate, with similar results. The error bars represent means and SD of the data. Use of log scale *y* axis was necessary for large differences in PRINT efficiency.

### Genomic DNA purification and ddPCR

Methods followed previous work (*18*). In brief, genomic DNA was digested for 2 hours with Bam HI and Xmn I (NEB). Multiplex 24-μl ddPCR reactions were prepared by mixing 12 μl of ddPCR supermix (no dUTP; Bio-Rad, 1863024), forward and reverse primers for target and reference genes (IDT, 833 nM final concentration each), probes complementary to target and reference amplicons (IDT; FAM for target and HEX for reference, 250 nM final concentration each), and digested genomic DNA at a final concentration of 1 to 5 ng/μl. Oligonucleotide sequences are listed in table S1. Reaction mix was transferred to a DG8 cartridge (Bio-Rad, 1864007) along with 70 μl of droplet generation oil (Bio-Rad, 1863005), and droplets were generated in a Bio-Rad QX200 Droplet Generator. The droplets were thermal cycled under the manufacturer’s recommended conditions with an annealing and/or extension temperature of 56°C and analyzed using QX Manager software with default settings. *RPP30* (copy number of 3 in RPE cells) was used as the reference gene for all copy number analysis.

### Pull-down of TPRT initiation complexes for cryo-EM analysis

The 76-bp 28*S* DNA target with 5′ biotinylated second strand was annealed separately. TPRT initiation complex was assembled by incubating 160 nM pre-annealed 76-bp 28*S* DNA target, 250 to 300 nM R2p, 300 nM 3′UTR RNA, bdSENP1 protease (1 μg/ml; Addgene plasmid 104962), and 100 μM of 2′,3′-dideoxythymidine (ddTTP) in 1-ml total volume in pull-down buffer [25 mM Hepes-KOH (pH 7.9), 400 mM potassium acetate, 10 mM magnesium acetate, and 1 mM Tris(2-chloroethyl) Phosphate (TCEP)]. While PlaMe TPRT initiation complex was assembled with full-length *Gf* 3′UTR RNA terminating in 5-nt rDNA homology (Gf-full), TaGu TPRT complex was assembled with the shorter Gf-98 RNA terminating in 5-nt rDNA homology due to low particle density when TaGu complex was assembled with Gf-full RNA. The complex was assembled on a rotator and incubated for 30 min at 37°C. Streptavidin Sepharose High-Performance resin (80 μl; Cytiva) was prewashed and incubated with the pull-down reaction at room temperature for 30 min. The flowthrough was removed, and the beads were washed twice with 0.5 ml of pull-down buffer. The elution was performed for 30 min at 37°C in the presence of 5 mM desthiobiotin and 5 μl of Pvu II (NEB, R3151S). The input, flowthrough, washes, and elution samples were analyzed on an SDS-PAGE and denaturing PAGE gels and stained with Coomassie blue and SYBR Gold (Thermo Fisher Scientific) stains, respectively. The pull-down eluate was concentrated to the smallest volume of 30 μl using Amicon Ultra-0.5 30-kDa molecular weight cut-off (MWCO) centrifugal filter. The eluted protein complex was directly applied to the EM grids for cryo-EM sample preparation.

### Pull-down of second-strand cleavage complex for cryo-EM analysis

28*S* DNA target with pre-nicked first strand to mimic synthesized Gf-68 cDNA, 5′ biotinylated second strand, and Gf-68 RNA with 5-nt rDNA homology were annealed separately. Sub-stoichiometric RNA concentration of 0.7× was used to anneal the cDNA substrate. Second-strand synthesis complex was assembled by incubating 160 nM cDNA substrate, 250 to 300 nM R2p, bdSumo protease (1 μg/ml), and 100 μM of ddCTP in 1-ml total volume in pull-down buffer [25 mM Hepes-KOH (pH 7.9), 400 mM potassium acetate, 10 mM magnesium acetate, and 1 mM TCEP]. The complex was assembled on a rotator and incubated for 30 min at 37°C. Streptavidin Sepharose High-Performance resin (80 μl; Cytiva) was prewashed and incubated with the pull-down reaction at room temperature for 30 min. The flowthrough was removed, and the beads were washed twice with 0.5 ml of pull-down buffer. The elution was performed for 30 min at 37°C in the presence of 5 mM desthiobiotin and 5 μl of Pvu II enzyme (NEB, R3151S). The input, flowthrough, washes, and elution samples were analyzed on an SDS-PAGE and denaturing PAGE gels and stained with Coomassie blue and SYBR Gold (Thermo Fisher Scientific) stains, respectively. The pull-down eluate was concentrated to the smallest volume of 30 μl using Amicon Ultra-0.5 30-kDa MWCO centrifugal filter. The eluted protein complex was directly applied to the EM grids for cryo-EM sample preparation.

### Cryo-EM grid preparation and data collection

Preparation of graphene oxide grids was adapted from our previously developed protocol (*38*). Briefly, Quantifoil Au/Cu R1.2/1.3 grids 200-mesh (Quantifoil, Micro Tools GmbH, Germany) were cleaned by applying two drops of chloroform and then glow discharged. Four microliters of polyethylenimine HCl MAX Linear Mw 40k (1 mg/ml; PEI, Polysciences) in 25 mM K-Hepes (pH 7.5) was applied to the grids, incubated for 2 min, blotted away, washed twice with H_2_O, and dried for 15 min on Whatman paper. Graphene oxide (Sigma-Aldrich, 763705) was diluted to 0.2 mg/ml in H_2_O, vortexed for 30 s, and precipitated at 1200*g* for 60 s. Supernatant (4 μl) was applied to the PEI treated grids, incubated for 2 min, blotted away, washed twice with 4 μl of H_2_O each, and dried for 15 min on Whatman paper before using for grid preparation. R2 complex (4 μl) was applied to the freshly prepared graphene oxide–coated grid and incubated for 60 s at 12°C and 100% humidity in a Vitrobot Mark IV (Thermo Fisher Scientific). The grid was then blotted for 1 s with a blot force of 1 and vitrified by plunging into liquid ethane.

For the PlaMe TPRT initiation complex, micrographs were collected on a Titan Krios microscope (Thermo Fisher Scientific) G3i with a BIO Quantum Energy Filter operated at 300 keV and equipped with a K3 Summit direct electron detector (Gatan). A total of 6425 movies were recorded using the program SerialEM at a nominal magnification of ×105,000 in super-resolution mode (super-resolution pixel size of 0.405 Å/pixel) and with a defocus range of −1.5 to −2.5 μm. The electron exposure was about 50 electrons (e^−^)/Å^2^. Each movie stack contained 50 frames. The same procedure was followed for the TaGu TPRT initiation complex to record 5096 movies. For the PlaMe second-strand–nicked complex, micrographs were collected on a Talos Arctica microscope (Thermo Fisher Scientific) operated at 200 keV and equipped with a K3 Summit direct electron detector (Gatan). A total of 9192 movies were recorded using the program SerialEM at a nominal magnification of ×36,000 in super-resolution mode (super-resolution pixel size of 0.57 Å/pixel) and with a defocus range of −1.5 to −2.5 μm. The electron exposure was about 50 e^−^/Å^2^. Each movie stack contained 50 frames.

### Cryo-EM data processing

Cryo-EM data processing workflows are outlined in figs. S4 and S10. All movie frames were motion corrected using MotionCor2 (*39*) in RELION 3.1.1 (*40*), and the corresponding super-resolution pixels size was binned ×2 during this process. Contrast transfer function (CTF) parameters for each micrograph were estimated using CTFFIND4.1 (*41*). Motion-corrected micrographs were imported into cryoSPARC v.4.5, and particles were picked using Blob Picker. Two-dimensional (2D) classification was performed in cryoSPARC. A total of 400,309 particles for the PlaMe first-strand synthesis complex, 763,427 particles for the TaGu first-strand synthesis complex, and 77,001 particles for the PlaMe second-strand cleavage complex were imported back to RELION; 3D initial models were generated; and 3D classification with alignment was performed for each dataset. The class for the PlaMe second-strand cleavage complex with 32,239 particles was further refined. Due to the limited number of particles, no further processing was carried out. For PlaMe and TaGu first-strand synthesis complexes, the classes with the best features were selected and refined, particles were polished with Bayesian polishing, and these classes were subjected to one round of 3D classification without alignment on the entire complex. The best class with sharpest features was selected and refined. The final reconstruction was obtained at 3.2-Å nominal resolution from 30,692 particles for the PlaMe complex and 3.3-Å nominal resolution from 18,892 particles for the TaGu complex. The cryo-EM maps were sharpened with post-processing in RELION for model building and display in the figures.

### Model building and refinement

Model building was initiated by rigid-body fitting the AlphaFold3 (*31*) model of PlaMe and TaGu proteins engaged with rDNA target into the final cryo-EM density maps using UCSF ChimeraX (*42*). The PlaMe and TaGu proteins were first manually inspected in Coot (*43*) and then subjected to real-space refinement in PHENIX (*44*). Amino acid side chains were manually inspected in Coot and modified when needed before another round of real-space refinement in PHENIX. Ribosomal DNA target and 3′UTR RNA were built starting with the BoMo structure [Protein Data Bank (PDB), 8GH6]. The parts of DNA target, particularly the single-stranded DNA, that did not fit the density were built de novo in Coot. RNA sequence was corrected to reflect the sequence used in experimental structures. Parts of the RNA were manually built de novo in Coot. The model was corrected to include an unincorporated dTTP obtained from PDB 1CR1. Both were docked into the density map using UCSF Chimera and manually rebuilt with the corresponding DNA chain in Coot. Four zinc atoms were manually placed in each structure and refined in Coot. The model was subjected to global refinement using iterative rounds of real-space refinements in PHENIX with rotamer and Ramachandran restraints. The complete model was subjected to a final real-space refinement and validation in PHENIX. Model building and validation statistics are listed in table S2.

### Comparison with *B. mori* R2 RT

*B. mori* R2 RT (PDB, 8GH6) was aligned with the vertebrate R2p chains using the MatchMaker tool in UCSF ChimeraX.
