# Supplementary material for: Structures of vertebrate R2 retrotransposon complexes during target-primed reverse transcription and after second-strand nicking
Source: Sci Adv. 2025 Jun 20;11(25):eadu5533. doi: 10.1126/sciadv.adu5533 (PMC12180492; doi:10.1126/sciadv.adu5533)
Supplement: Supplementary file 1 — Figs. S1 to S11 Tables S1 and S2 [file sciadv.adu5533_sm.pdf]

Science Advances
AAAS

Supplementary Materials for

Structures of vertebrate R2 retrotransposon complexes during target-primed reverse transcription and after second-strand nicking

Akanksha Thawani *et al.*

Corresponding author: Akanksha Thawani, athawani@berkeley.edu; Eva Nogales, enogales@lbl.gov; Kathleen Collins, kcollins@berkeley.edu

Sci. Adv. **11**, eadu5533 (2025)
DOI: 10.1126/sciadv.adu5533

This PDF file includes:

Figs. S1 to S11
Tables S1 and S2

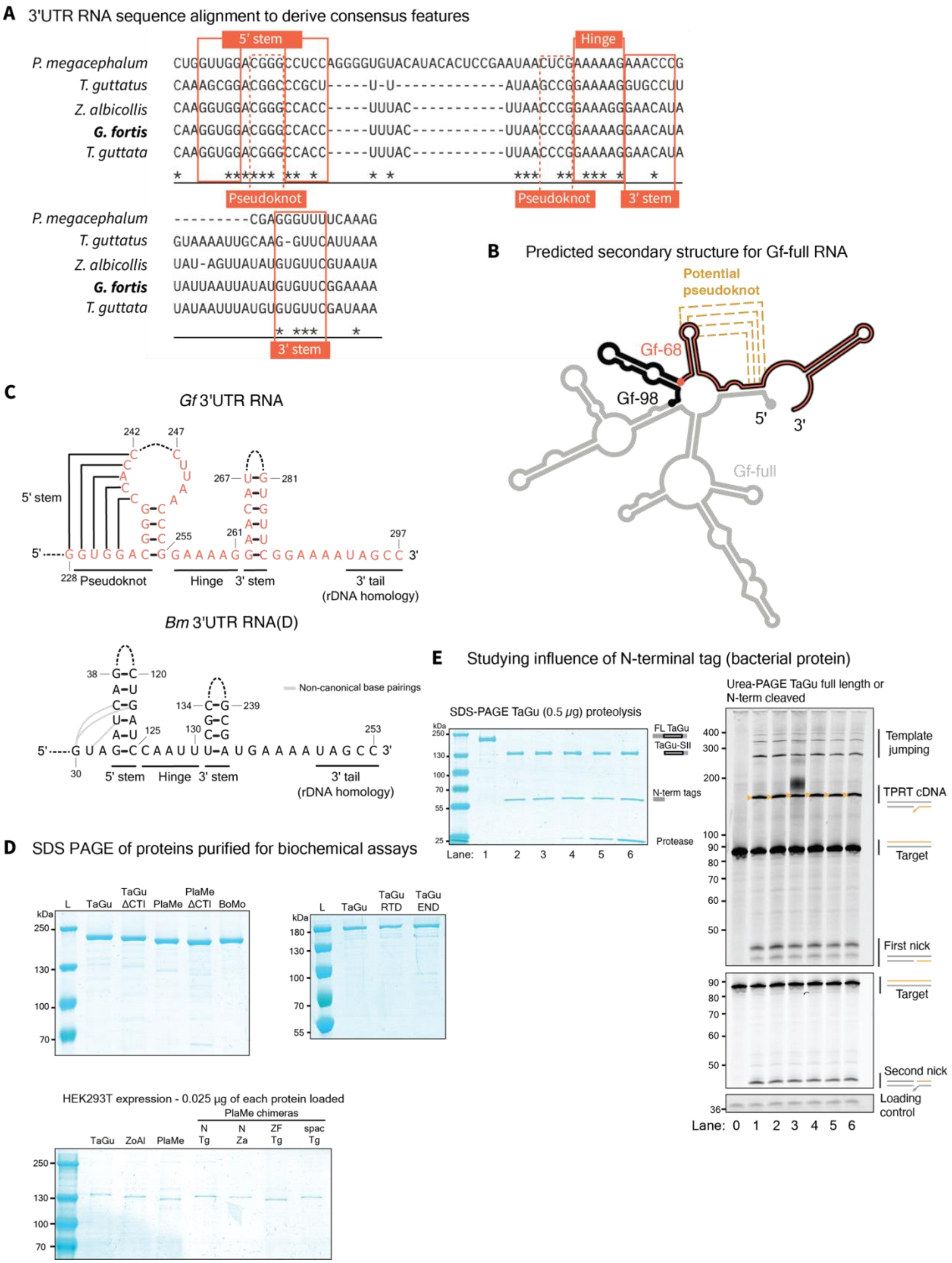

Figure S1. R2 terminal 3′UTR sequence alignment and biochemical assays. (A) Multiple

sequence alignment of the 3′-terminal regions of 3′UTR RNAs from A-clade avian (bottom four species) and testudine (*P. megacephalum)* R2, using species with R2p described in the main text or in a previous work (ref: 18). Numbering is from the start of the aligned region only. Nucleotide identity is indicated with an asterisk, and regions indicated are a set of at first putative features suspected to constitute the RNA cores: 5′ stem, pseudoknot, hinge, 3′ stem and 3′ tail. (B) Secondary structure prediction depicted for Gf-full (gray), generated using RNAfold v2.6.3 and visualized with a modified PseudoViewer structure, RNApdbee v3. Resulting RNA sequences from truncations Gf-98 (black) and Gf-68 (red) are mapped. Each large dot indicates the first 5′ nucleotide of each RNA. Truncations were hypothesized to have potential to sample a pseudoknot conformation (yellow dashed lines) in the regions indicated. (C) Secondary structure of the *Gf* 3′UTR RNA portions resolved in the TPRT initiation complexes for PlaMe and TaGu is compared with the secondary structure of the D-clade *Bm* 3′UTR RNA resolved in PDB 8gh6. The pseudoknot and 5′ stem base-pairing interactions are represented in solid black lines for *Gf* 3′UTR and non-canonical interactions are displayed in gray lines for *Bm* 3′UTR; unresolved portions of the RNA are represented with dashed lines. (D) Coomassie blue stained SDS PAGE gels showing all wild-type and variant versions of R2p used for *in vitro* TPRT assays. All proteins used for TPRT retained their tag fusions. (E) Left: Coomassie blue stained SDS PAGE gels showing products of proteolysis using bdSENP1 at increasing concentrations incubated overnight at 4 ºC with 1.25 µM of TaGu (0.5 µg loaded). Protease dilutions included: no protease (lane 1), 45 nM (lane 2), 90 nM (lane 3), 450 nM (lane 4), 0.92 µM (lane 5), 4.6 µM (lane 6). Proteolysis buffer contained a final concentration of 290 mM sodium chloride, 25 mM HEPES pH 7.7, 5 mM magnesium chloride, 6 mM BME, and 10 % (v/v) glycerol. Right: Denaturing PAGE analysis of TPRT reaction products using proteins incubated with the same buffer with or without protease shown on the left. R2p had a final concentration of 30 nM for TPRT. Gf-98 with R5 (400 nM) and target site DNA (12 nM) were used across reactions. Orange triangles indicate expected TPRT product lengths for copying a single full-length template (TPRT cDNA). Multiple templates may also be copied in series (template jumping products). Different regions of the same gel are shown, with first strand DNAs and second strand DNAs detected separately using different 5′ dyes. Loading control is detected by staining with SYBR Gold.

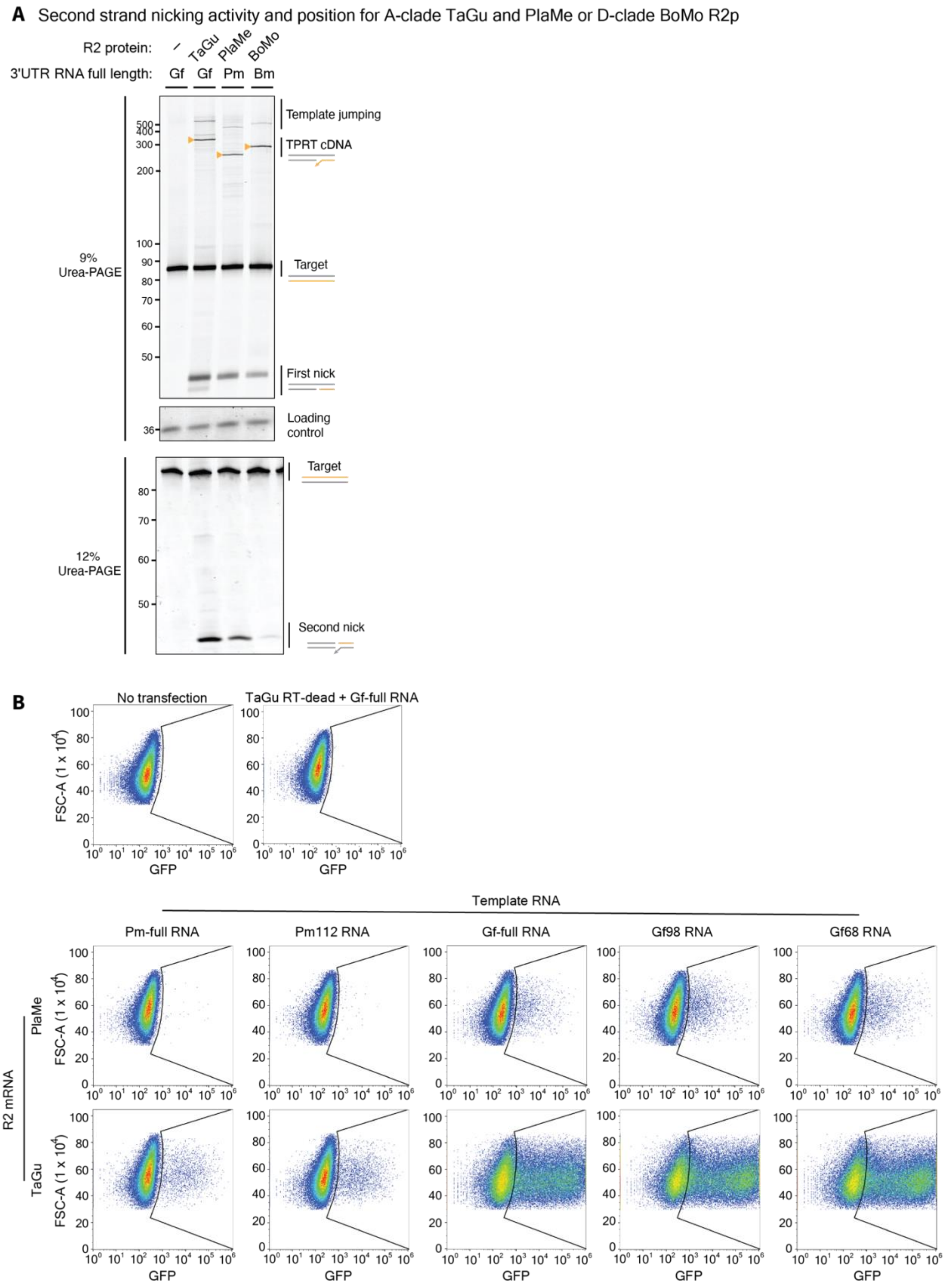

A Second strand nicking activity and position for A-clade TaGu and PlaMe or D-clade BoMo R2p
R2 protein: – TaGu PlaMe BoMo
3'UTR RNA full length: Gf Gf Pm Bm
9% Urea-PAGE
Template jumping
TPRT cDNA
Target
First nick
Loading control
12% Urea-PAGE
Target
Second nick
B
No transfection
TaGu RT-dead + Gf-full RNA
Template RNA
Pm-full RNA
Pm112 RNA
Gf-full RNA
Gf98 RNA
Gf68 RNA
R2 mRNA
PlaMe
TaGu
FSC-A (1 x 10⁴)
GFP

Figure S2. Second nick analysis across A- and D-clade proteins and representative flow cytometry for PRINT experiments. (A) Denaturing PAGE analysis of TPRT reaction products using three R2 proteins (TaGu, PlaMe and BoMo) (30 nM each), each with their associated full length 3′UTR RNA (400 nM) with a 3′ tail terminating with R5. Target site DNA was used at 12 nM. Two separate denaturing PAGE gels were loaded with the same reactions to detect first strand DNAs (9% denaturing PAGE) and second strand DNAs (12% denaturing PAGE) using different 5′ dyes. The higher bis-acrylamide percentage was used to more precisely compare the migration of second nick products. Loading control is detected by staining with SYBR Gold. Orange triangles indicate expected TPRT product lengths for copying a single full-length template (TPRT cDNA). Multiple templates may also be copied in series (template jumping products). (B) Representative flow cytometry data from one replicate of PRINT data in Figure 1e are displayed. The gating of GFP+ cells is demarcated with black lines. The x-axis is GFP intensity, and the y-axis (FSC-A is forward scatter area) approximates cell size. Panels on the top show negative controls for non-transfected cells and cells transfected with the GF-full RNA template and the RT-dead version of TaGu.

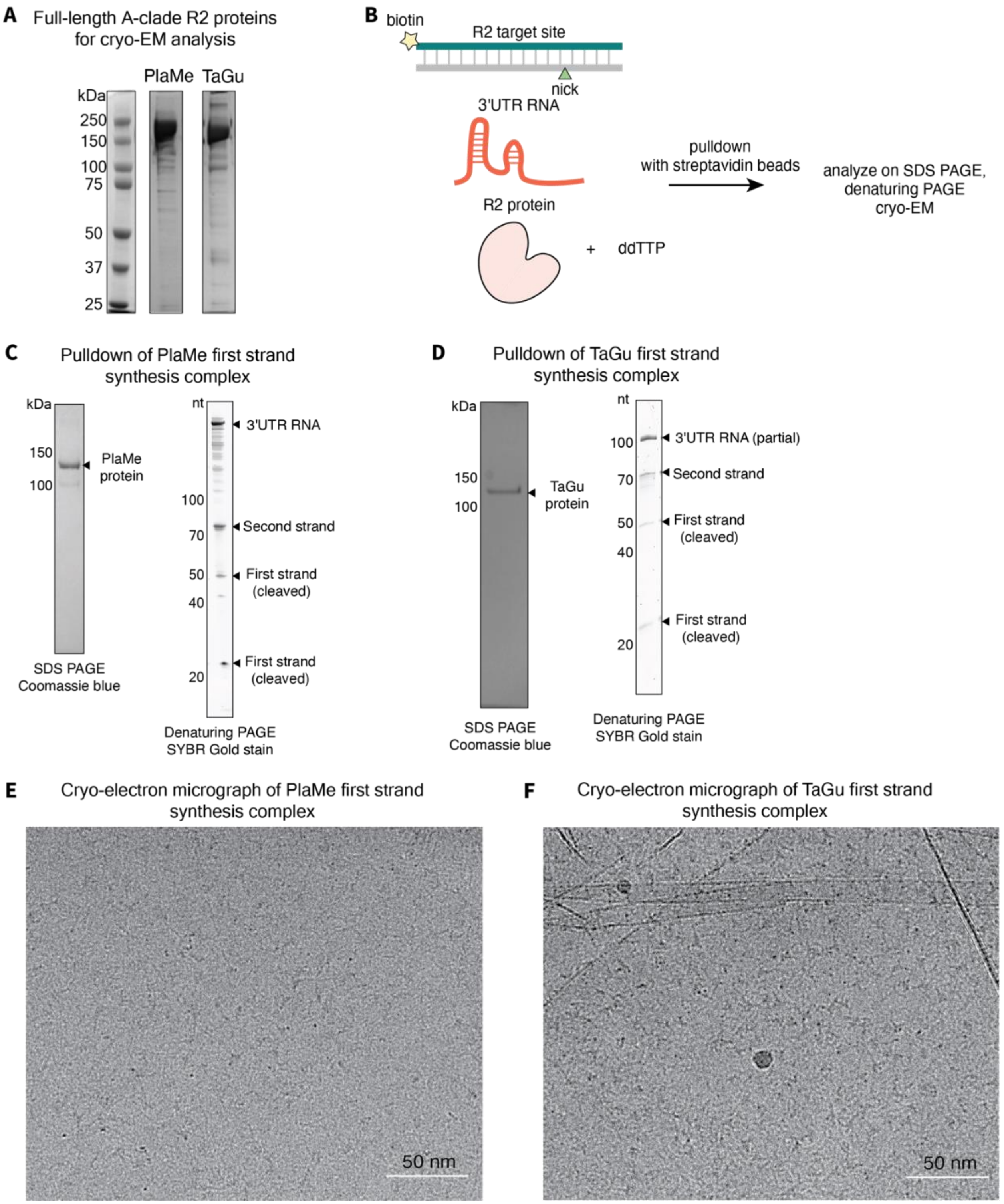

Figure. S3. Assembly of TPRT initiation complexes for cryo-EM analysis. (A) SDS PAGE of purified full-length PlaMe and TaGu proteins after Strep-affinity and Heparin purification for cryo-EM analysis. (B) Schematic of R2 complex assembly during TPRT. R2 proteins were incubated with biotinylated target site DNA, 3′UTR RNA (full-length or truncated) and ddTTP for production of the TPRT initiation state. PlaMe complex was assembled with Gf-full RNA, whereas TaGu complex with Gf-98 RNA. (C) SDS PAGE analysis of protein and denaturing PAGE

analysis of nucleic acids in the pulldown eluate for the PlaMe TPRT initiation complex. Gf-full RNA was used. (D) SDS PAGE analysis of protein and denaturing PAGE analysis of nucleic acids in the pulldown eluate for the TaGu TPRT initiation complex. Gf-98 RNA was used. (E) Representative cryo-EM micrograph of the pulldown eluate for PlaMe captured during TPRT initiation. (F) Representative cryo-EM micrograph of the pulldown eluate for TaGu captured during TPRT initiation.

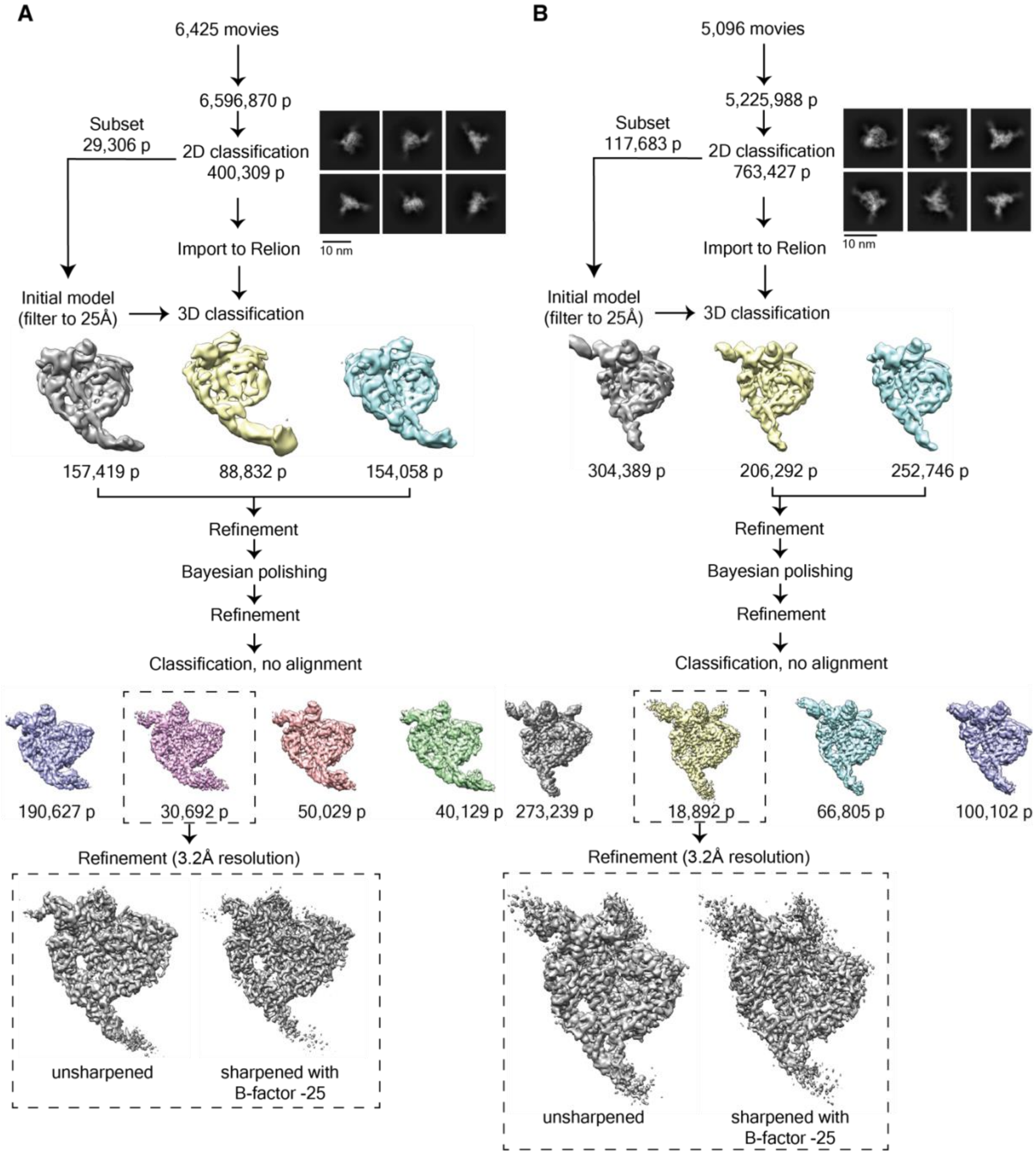

Figure S4. Cryo-EM data processing pipeline used for the PlaMe and TaGu first strand synthesis complexes. Single particle analysis workflow leading to the reconstruction of the (A) PlaMe and (B) TaGu TPRT initiation complexes described in Figures 1-4. Densities for the final structures are shown both before and after sharpening.

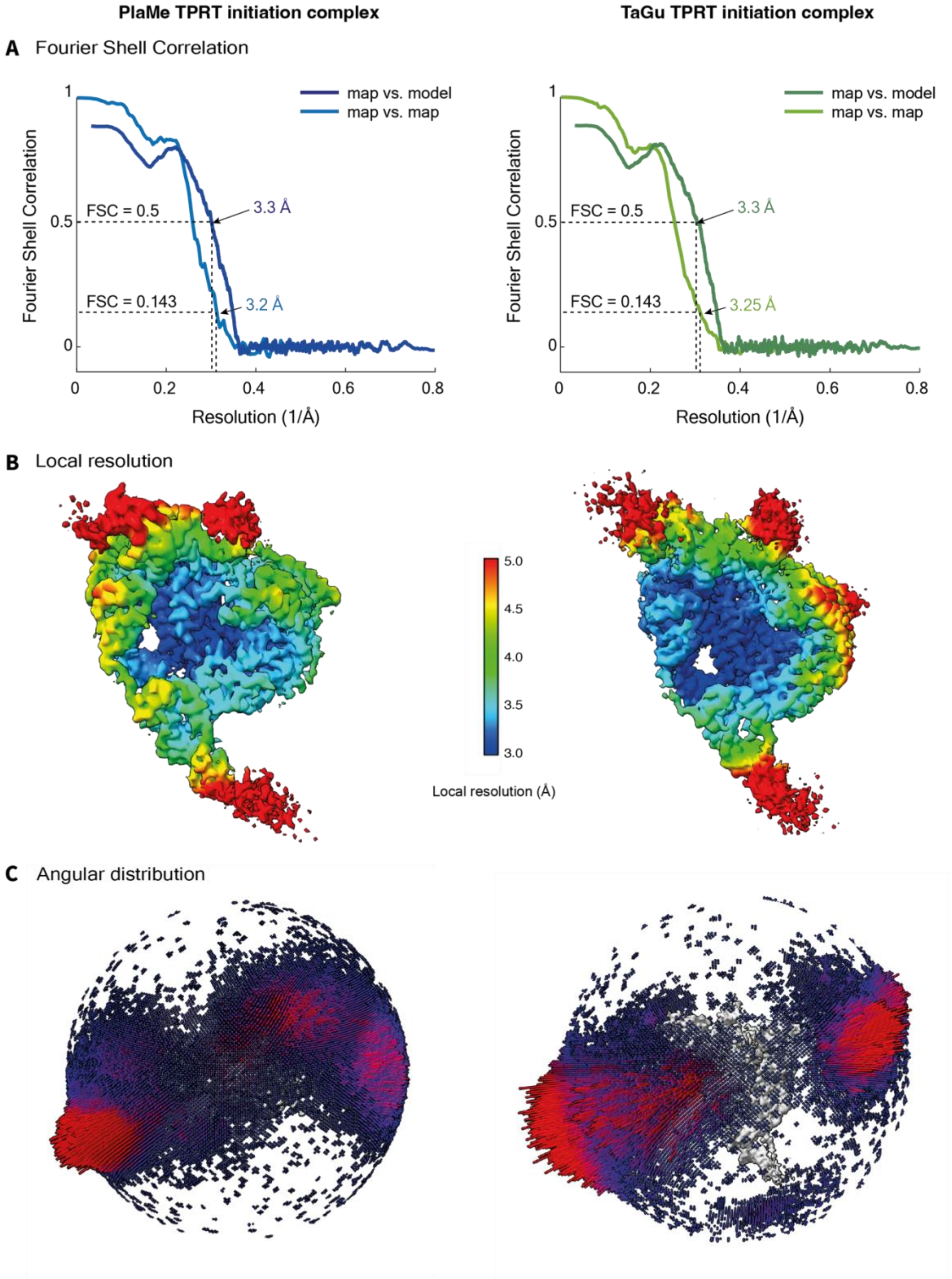

Figure S5. Resolution estimation. (A) Gold-standard Fourier shell Correlation (FSC) curve and map versus model FSC obtained from the final model after validation in Phenix for the PlaMe (left) and TaGu (right) TPRT initiation complexes. (B) Unsharpened density maps obtained from

analysis in Figure S4 were colored by local resolution as estimated using Relion 3.1. (C) Particle orientation distribution in the final reconstructions.

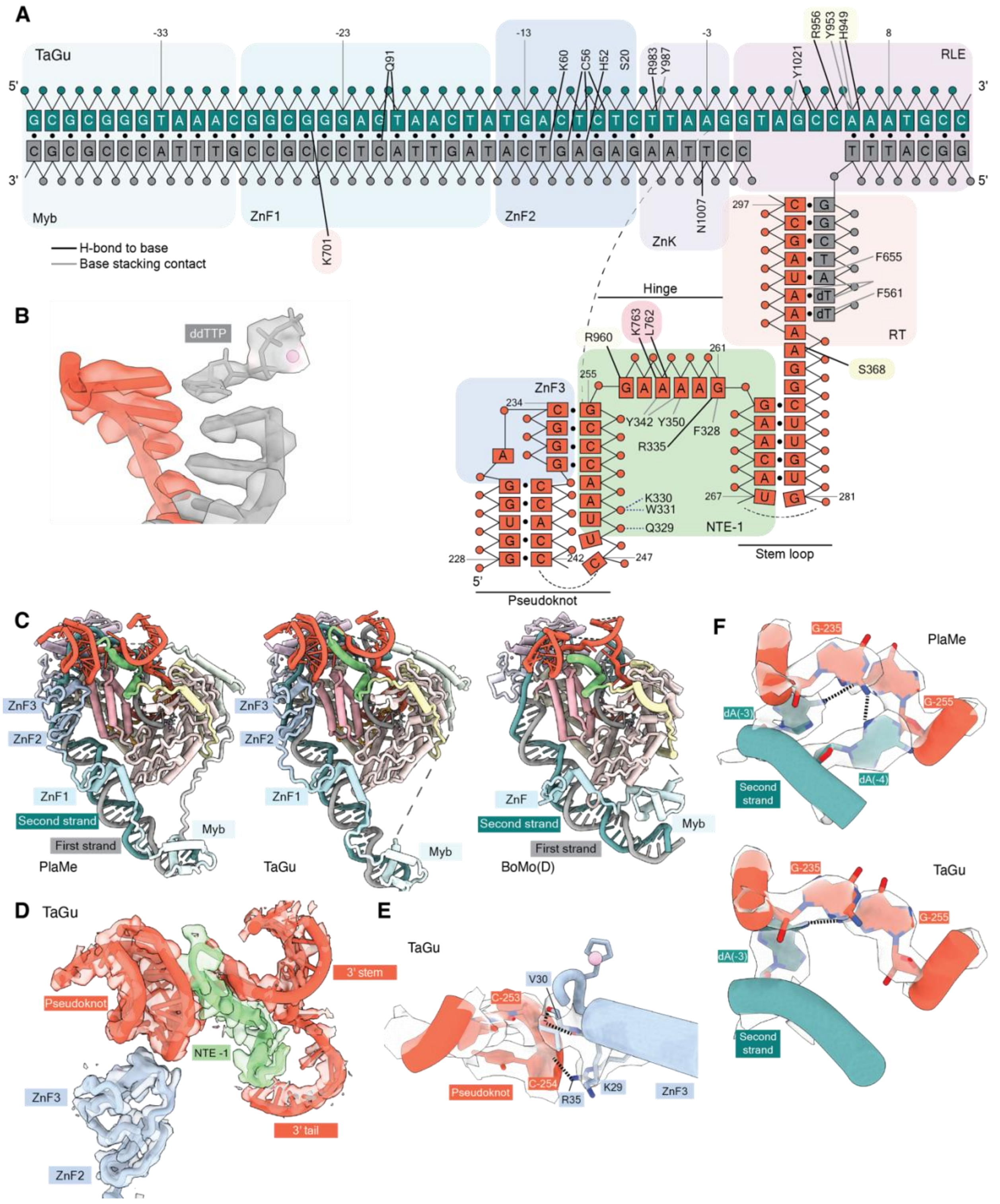

Figure S6. Nucleic acid interactions by the TaGu protein during TPRT initiation. (A) Schematic of direct interactions between TaGu protein, target site DNA, and 3′UTR RNA. Color scheme and labeling are consistent with Figure 1. Solid black lines denote sequence-specific hydrogen bonds between protein residues and nucleobases or ribonucleobases, while the dashed black line represents hydrogen bonding between target site DNA and RNA bases. Solid gray lines denote pi-stacking contacts with the nucleobases or ribonucleobases. Black circles represent base-

pairs. (B) The RT active site harbored an unincorporated ddTTP that was resolved with a coordinated Mg^{2+} ion (sphere). The transparent density represents the cryoEM map. (C) Overall structures of the A-clade PlaMe and TaGu TPRT initiation complexes are compared with the D-clade BoMo TPRT initiation complex from PDB 8gh6. (D) 3′UTR RNA is engaged by the NTE -1 and ZnF3-2 domains of TaGu protein. ZnF3 domain from TaGu contacts the pseudoknot of 3′UTR RNA. The transparent density represents the cryoEM map. (E) Side chains in TaGu ZnF3 make contact with the phosphate backbone of base C-253 and C-254 at the junction of hinge and pseudoknot. The helix segmentation is an artifact of automated secondary structure assignment. The transparent density represents the cryoEM map. (F) Base-specific hydrogen bond(s) between pseudoknot bases and base(s) in a single-stranded region of the second strand DNA. The transparent density represents the cryoEM map.

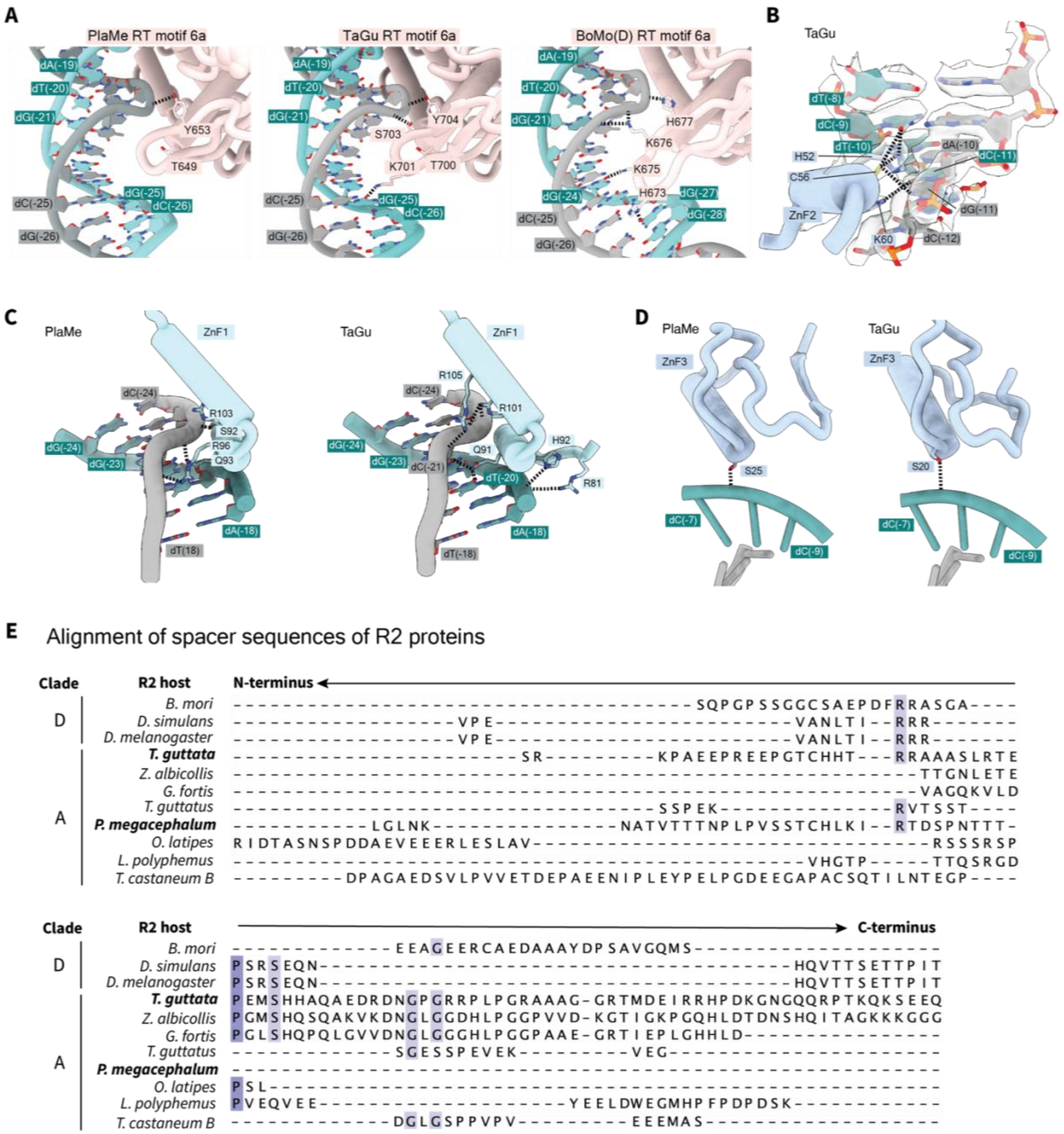

Figure S7. Target DNA engagement by R2 proteins. (A) Hydrogen bonds between RT motif 6a and upstream target site DNA compared across PlaMe, TaGu and BoMo TPRT initiation structures. (B) Base-reading hydrogen bonds between ZnF2 of TaGu and the target site DNA proximal to the nick site. The transparent density represents the cryoEM map. (C) Target site recognition by ZnF1 occurs predominantly by sequence non-specific hydrogen bonds with the DNA backbone, shown for PlaMe (left) and TaGu (right). TaGu Q91 side chain makes two base-specific contacts with dC(-21) and dT(-20), whereas no base-specific contacts are made by any PlaMe ZnF1 side chains. (D) ZnF3 has minimal, sequence non-specific hydrogen bonding with the DNA backbone. (E) The Spacer sequences between Myb domain and NTE -2 were aligned for representative D-clade R2p (rows 1-3) and A-clade R2p (rows 4-11). Spacer boundaries were defined using AlphaFold3 models. Purple shading illustrates relative sequence conservation.

Species not given in main text: *Oryzias latipes*, *Limulus polyphemus*, *Tribolium castaneum B*, and *Drosophila simulans* or *melanogaster* (ref: 14).

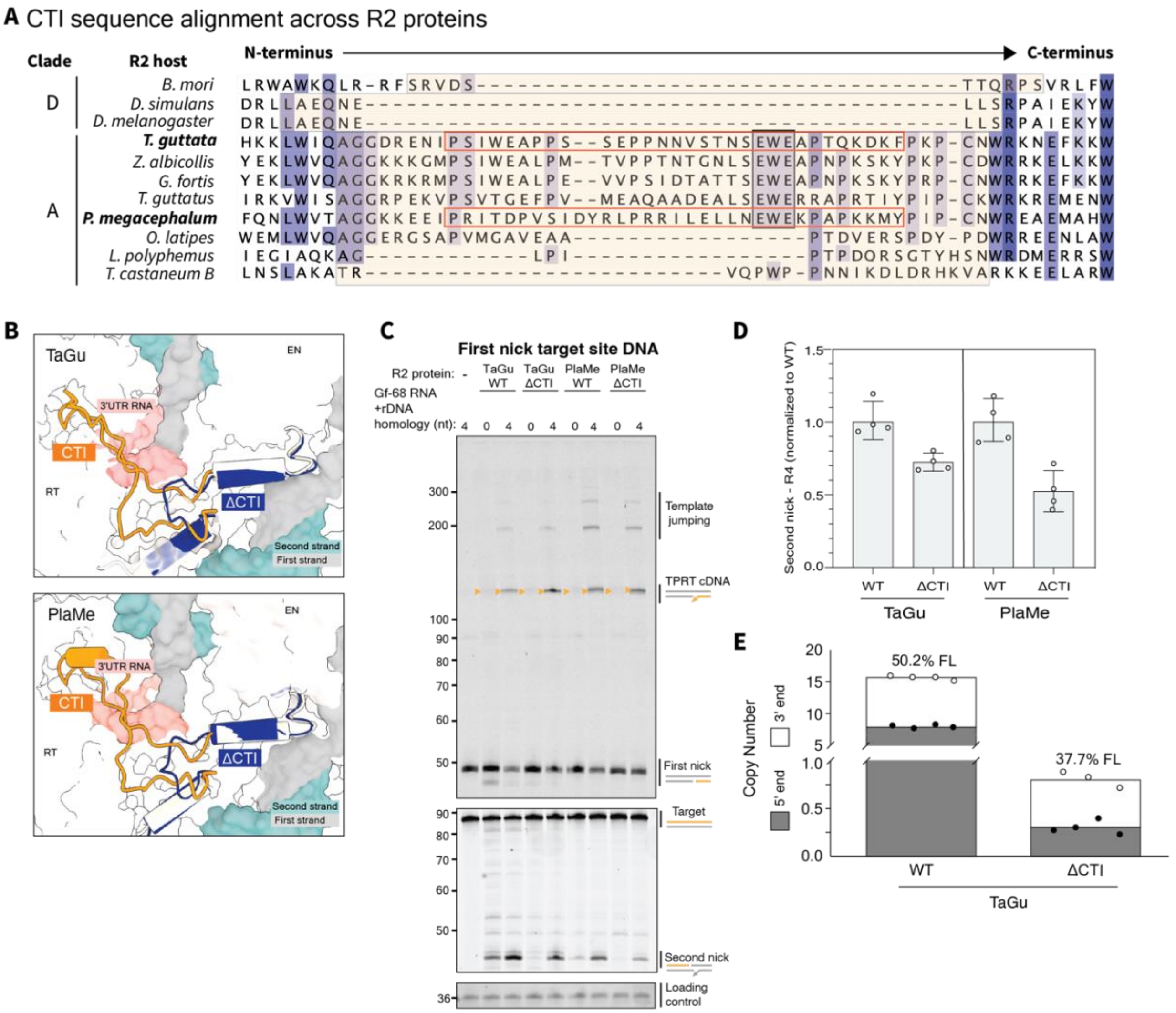

Figure S8. CTI sequence alignment and its influence on TPRT activity and full-length transgene insertion. (A) The CTI (bounded by peach-colored background) and its surrounding sequences were aligned for representative D-clade R2p (rows 1-3) and A-clade R2p (rows 4-11). The CTI boundaries were defined using AlphaFold3 models. The conserved EWE anchor in aligned avian and testudine R2p is highlighted with a black box. Purple shading illustrates relative sequence conservation. Species not given in main text: *Oryzias latipes*, *Limulus polyphemus*, and *Drosophila simulans* or *melongaster* (ref: 14). The red boxes indicate amino acids in PlaMe and TaGu that were truncated in the ΔCTI mutants. (B) AlphaFold3 prediction for the ΔCTI TaGu or PlaMe aligned globally to the structure of the corresponding wild-type R2p, withthe alpha helices that flank the shorted CTI marked with blue. (C) Denaturing PAGE of TPRT reaction products with wild-type TaGu, TaGu ΔCTI mutant, wild-type PlaMe and PlaMe ΔCTI mutant. Target site DNA substrate mimicked the state after first strand nicking (Table S1). Gf-68 RNA used had a variable length of the 3′ tail (0 or 4 nt rDNA), with only R4 supporting template base-pairs to target site primer. Orange triangles indicate expected TPRT product lengths for copying a single full-length template (TPRT cDNA). Multiple templates may also be copied in series (template jumping products). Different regions of the same gel are shown, with first strand DNAs and second strand DNAs imaged separately using different 5′ dyes. Loading control is detected by staining with SYBR Gold. (D) Quantification of ΔCTI mutant R2p total cDNA, the sum of the signal from TPRT

cDNA and template jumps, normalized to each wild-type protein in from TPRT reactions with R4 in (C). (E) Genomic DNA from cells described in Figure 4d, after PRINT with wild-type or ΔCTI TaGu, was assayed by ddPCR for copy number of the inserted transgene 5′ or 3′ end. Copy numbers are graphed as stacked bars each starting from y-axis of zero, and the calculated percentage of full-length insertions is indicated above the bars (ref: 18).

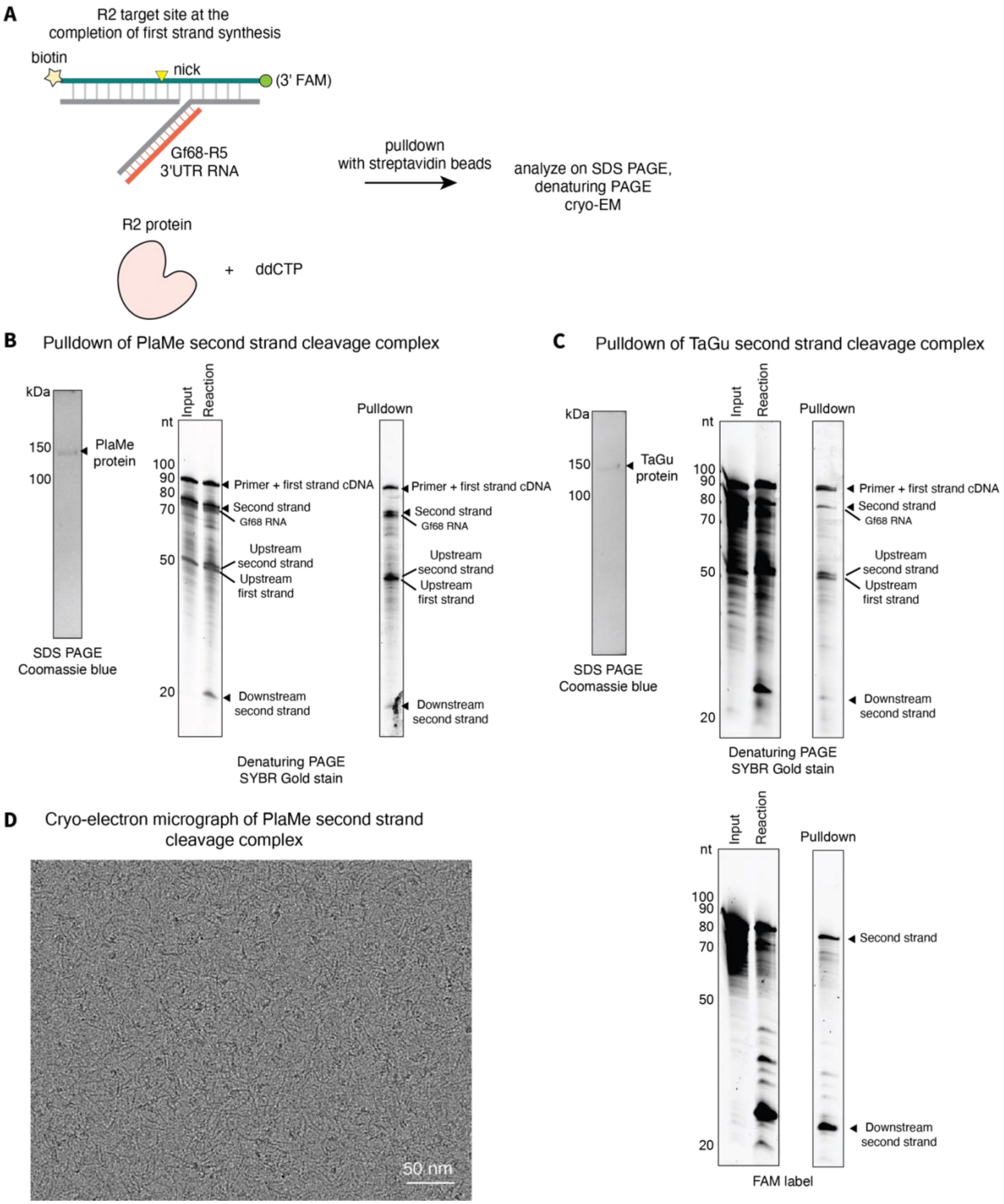

Figure S9. Assembly of second strand nicked complex for cryo-EM analysis. (A) PlaMe was incubated with biotinylated DNA containing the target site and cDNA, with cDNA annealed to template RNA, in a configuration that supports addition of a single ddCTP to complete first strand cDNA synthesis. (b-c) SDS PAGE protein analysis and denaturing PAGE nucleic acid analysis of the pulldown and elution for the second strand nicked complex with PlaMe in (B) and TaGu in (C). The eluate sample appears to be a mixed population of intact and nicked second strand. In the

lower panels in (C), fluorescence imaging of the same gel was performed to track nicking efficiency in TaGu samples with a target DNA with 3′ FAM-labeled second strand. (D) Cryo-EM micrographs of the pulldown eluate for PlaMe captured after second strand nicking.

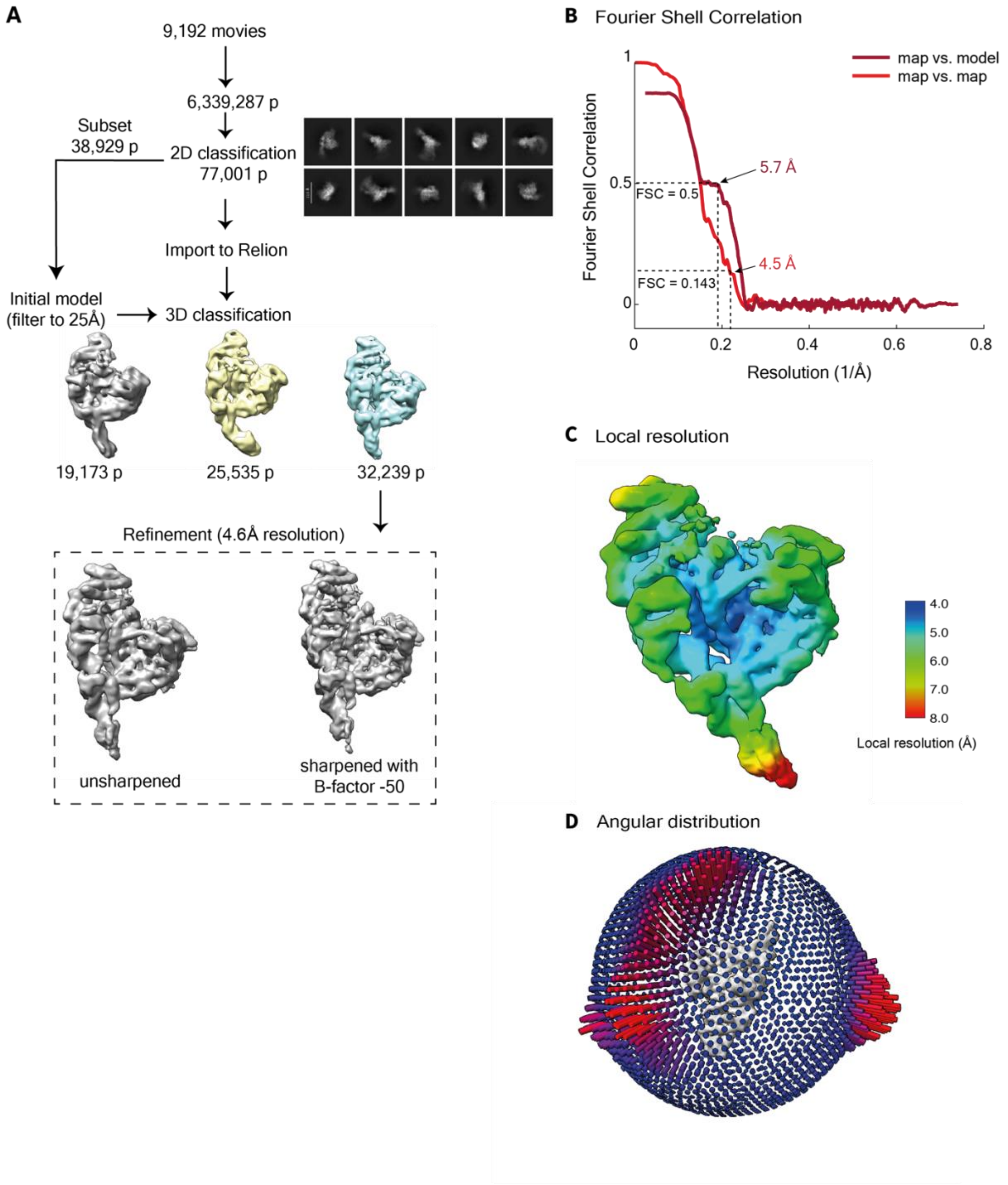

Figure S10. Cryo-EM data processing and resolution estimation for PlaMe second strand nicked complex. (A) Summary of single particle analysis pipeline leading to the reconstruction of the PlaMe second strand nicked complex described in Figure 5. (B) Gold-standard FSC curve and map versus model FSC obtained from the final model after validation in Phenix. (B) Unsharpened density map was colored by local resolution as estimated by Relion 3.1. (C) Particle orientation distribution in the final reconstructions.

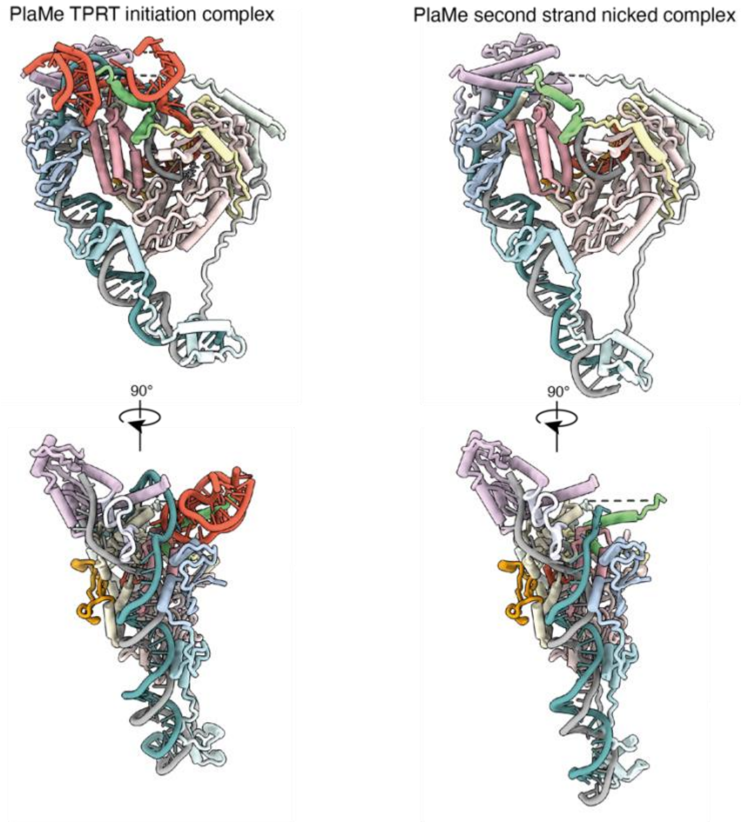

Figure S11. Comparison of PlaMe TPRT initiation and post-second nick structures. The PlaMe structures at the stages of TPRT initiation and after second strand nicking were aligned using the protein chain. Two distinct views of the structures are displayed.

Table S1. Protein and nucleic acid sequences used in this study

Name	Sequence	Purpose and figure
**Proteins**		
PlaMe	QKTIIQLPNDNPACPFCGDHVGKPSALNVHLKRNHGGRE<br>VEFQCSMCNKADPKAHSILCHIPKCKGKVTEEPTGDWAC<br>ETCNKQFNTKSGLSQHKRIAHPAIRNQERIAASQPKPNSQ<br>RGKHNSCWTVEEEQLLAAFNNMFWGKKNINILISDHIHM<br>KTAKQISEKRRLLGLNKNATVTTTNPLPVSSTCHLKIRTD<br>SPNTTTGLKDTYMCKINENIVNQGQIKFDSEVISAWMAG<br>DSNIRSLVESTSLDILSTFLMETPKPRKKGNNKITNKKSGK<br>KKKWMEKRAVKKGFYKRYQHLFETDRCKLASIILDGTE<br>RLQCQIPLTEILETYKSKWETLTPFEGLGQFKSHAVADNT<br>AFEILLSAKEIMKNIKEMNKNSAPGPDKVSLRDLLLADPE<br>CNALEKLFNTWLITGIIPNSIKECRSLLIPKTADPEALKELG<br>NWRPLTIGSIVLRLFSRIITNRLAKACPINARQRGFIATPGC<br>SENLKILHTIVKQAKTSKKSLGVVFVDIAKAFDSVSHDHI<br>MWVLQERGLDQHIVNIIEDSYKKIHTRMEVGTERTPPIEI<br>KVGVKQGDPMSPLLFNLAIDPLITALEKANTGFSYGKNKI<br>TSLAFADDLVMLSDTWEGMNKNIQILETFCNLSGLKVQA<br>KKCYGFFLSPTHDSYTINKCDAWKIDKDSLNMIQPGESE<br>KYLGLKVDPWIGFSKPVLAEKLTIWLKRLTEAPLKPSQK<br>LTMLNIYTIPRIIYLADHTDTKKTLLSSLDDNIRTVVKGW<br>LHLPPDTCNGFIYTKTRDGGLGVTRLASLIPSIQARRLHRI<br>ATSEDETIRNIAMANNIEEEFQNLWVTAGGKKEEIPRITD<br>PVSIDYRLPRRILELLNEWEKPAPKKMYPIPCNWREAEM<br>AHWKNLPCQGSGIEHFDNDTISNDWLQFHRGFSERQFLM<br>GLKIRANVYPTREYQGRGRTNKNVNCRNCTASYESLSHI<br>LGQCPAVQGARIRRHNKLCSMLKREAKELKWVVYEEPH<br>LHTTEKELRKPDLIFVKEEMALVVDVTVRFEYKEKVFED<br>AAAEKVRHYKDLTSQIKELTGAKEIEYFGFPLGARGKWP<br>EINEKVLTALGMPDYQQKRTAKRFSKRTLLYSIDVINTFE<br>NIGKNNKNNVP	All figures
TaGu	MVTVPDKNPPCPCCGTRVNSVLNLIEHLKVSHGKRGVCF<br>RCAKCGKENSNYHSVVCHFPKCRGPETEKAPAGEWICE<br>VCNRDFTTKIGLGQHKRLAHPAVRNQERIVASQPKETSN<br>RGAHKRCWTKEEEELLIRLEAQFEGNKNINKLIAEHITTK<br>TAKQISDKRRLLSRKPAEEPREEPGTCHHTRRAAASLRTE<br>PEMSHHAQAEDRDNGPGRRPLPGRAAAGGRTMDEIRRH<br>PDKGNGQQRPTKQKSEEQLQAYYKKTLEERLSAGALNT<br>FPRAFKQVMEGRDIKLVINQTAQDCFGCLESISQIRTATR<br>DKKDTVTREKHPKKPFQKWMKDRAIKKGNYLRFQRLFY<br>LDRGKLAKIILDDIECLSCDIPLSEIYSVFKTRWETTGSFKS<br>LGDFKTYGKADNTAFRELITAKEIEKNVQEMSKGSAPGP<br>DGITLGDVVKMDPEFSRTMEIFNLWLTTGKIPDMVRGCR<br>TVLIPKSSKPDRLKDINNWRPITIGSILLRLFSRIVTARLSK<br>ACPLNPRQRGFIRAAGCSENLKLLQTIIWSAKREHRPLGV<br>VFVDIAKAFDTVSHQHIIHALQQREVDPHIVGLVSNMYE<br>NISTYITTKRNTHTDKIQIRVGVKQGDPMSPLLFNLAMDP<br>LLCKLEESGKGYHRGQSSITAMAFADDLVLLSDSWENM<br>NTNISILETFCNLTGLKTQGQKCHGFYIKPTKDSYTINDC<br>AAWTINGTPLNMIDPGESEKYLGLQFDPWIGIARSGLSTK<br>LDFWLQRIDQAPLKPLQKTDILKTYTIPRLIYIADHSEVKT<br>ALLETLDQKIRTAVKEWLHLPPCTCDAILYSSTRDGGLGI<br>TKLAGLIPSVQARRLHRIAQSSDDTMKCFMEKEKMEQLH	All figures

	KKLWIQAGGDRENIPSIWEAPPSSEPPNNVSTNSEWEAPT<br>QKDKFPKPCNWRKNEFKKWTKLASQGRGIVNFERDKIS<br>NHWIQYYRRIPHRKLLTALQLRANVYPTREFLARGRQDQ<br>YIKACRHCDADIESCAHIIGNCPVTQDARIKRHNYICELLL<br>EEAKKKDWVVFKEPHIRDSNKELYKPDLIFVKDARALVV<br>DVTVRYEAAKSSLEEAAAEKVRKYKHLETEVRHLTNAK<br>DVTFVGFPLGARGKWHQDNFKLLTELGLSKSRQVKMAE<br>TFSTVALFSSVDIVHMFASRARKSMVM	
ZoAl	MNIVKVTVPDKNPPCPCCGVRLNSVLALIEHLKGSHGRR<br>RVCFRCAKCGRENFNHHSTVCHYAKCKGPQIERPPVGE<br>WICEVCGRDFTTKIGLGQHKRHMHAMVRNQERIDASQP<br>KETSNRGAHKRCWTKEEEELLMKLEVQFENHKNINKLIA<br>EQLTTKTAKQISDKRRMLLKKGRGTTGNLETEPGMSHQS<br>QAKVKDNGLGGDHLPGGPVVDKGTIGKPGQHLDTDNSH<br>QITAGKKKGGGLQARYRRRIMKRLAAGTINIFPKVFKELI<br>NDQEARPLINQTTEDCFGLLDSACQIRTALREKGKSQEER<br>PRKQYQKWMKKRAIKRGDYLRFQRLFHLDRGKLARIIL<br>DNTESLSCDISPSEIYSVFKARWETPGHFNGLGDFEIKGK<br>ANNKAFRDFITAKEIEKNVREMSKGSAPGPDGIALGDIKK<br>MDPGYSRTAELFNLWLTAGDIPDMVRGCRTVLIPKSTTP<br>ERLKDINNWRPITIGSILLRLFSRIITARMTKACPLNPRQR<br>GFISAPGCSENLKLLQSIIRTAKNEHKPLGVIFVDIAKAFD<br>TVSHQHIIHVLQQRRVDPHIVGLVNNMYKDISTYVTTKK<br>NTHTDKIQIRVGVKQGDPLSPLLFNLAMDPLLCKLEESG<br>KGFHRGQSSITAMAFADDLVLLSDSWENMKENIKILETF<br>CNLTGLKTQGQKCHGFYIKPTKDSYTINNCPAWTINGTP<br>LNMINPGESEKYLGLQIDPWTGVAKYDLSTKLKIWLESID<br>RAPLKPLQKLDILKTYTIPRLTYLADHSEMKAGALEALD<br>QQIRTAVKDWLHLPSCTCDAILYVSTRDGGLGVTKLAGL<br>IPSVQARRLHRIAQSPDETMKDFLEKAQMEKMYEKLWV<br>QAGGKKKGMPSIWEALPMTVPPTNTGNLSEWEAPNPKS<br>KYPKPCDWRRKELKKWTKLESQGRGVKNFRNDTISND<br>WIQYYRRIPHRKLLTAIQLRANVYPTREFLARGRGDNYV<br>KFCRHCEADLETCGHIIGFCPVTKDARIKRHNRICDRLCE<br>EAAKREWVVFKEPHLRDATTELFKPDVIFVKEDRALVVD<br>VTVRYESAKTTLEAAAMEKVDKYKHLEAEVKELTNAK<br>DVVFMGFPLGARGKFYKGNFNLLETLGLPKTRQLSVAK<br>TLSTYALMSSVDIVHMFASRSRKPNV	Fig. 1, Fig. 3, Fig. S1, Fig. S7, Fig. S8
BoMo	MKKSNKENRPEASGLPLESERTGDNPTVRGSAGADPVG<br>QDAPGWTCQFCERTFSTNRGLGVHKRRAHPVETNTDAA<br>PMMVKRRWHGEEIDLLARTEARLLAERGQCSGGDLFGA<br>LPGFGRTLEAIKGQRRREPYRALVQAHLARFGSQPGPSSG<br>GCSAEPDFRRASGAEEAGEERCAEDAAAYDPSAVGQMS<br>PDAARVLSELLEGAGRRRACRAMRPKTAGRRNDLHDDR<br>TASAHKTSRQKRRAEYARVQELYKKCRSRAAAEVIDGA<br>CGGVGHSLEEMETYWRPILERVSDAPGPTPEALHALGRA<br>EWHGGNRDYTQLWKPISVEEIKASRFDWRTSPGPDGIRS<br>GQWRAVPVHLKAEMFNAWMARGEIPEILRQCRTVFVPK<br>VERPGGPGEYRPISIASIPLRHFHSILARRLLACCPPDARQ<br>RGFICADGTLENSAVLDAVLGDSRKKLRECHVAVLDFA<br>KAFDTVSHEALVELLRLRGMPEQFCGYIAHLYDTASTTL<br>AVNNEMSSPVKVGRGVRQGDPLSPILFNVVMDLILASLP<br>ERVGYRLEMELVSALAYADDLVLLAGSKVGMQESISAV<br>DCVGRQMGLRLNCRKSAVLSMIPDGHRKKHHYLTERTF<br>NIGGKPLRQVSCVERWRYLGVDFEASGCVTLEHSISSAL<br>NNISRAPLKPQQRLEILRAHLIPRFQHGFVLGNISDDRLR	Fig. 1, Fig. S1, Fig. S2, Fig. S7, Fig. S8

	MLDVQIRKAVGQWLRLPADVPKAYYHAAVQDGGLAIPS<br>VRATIPDLIVRRFGGLDSSPWSVARAAAKSDKIRKKLRW<br>AWKQLRRFSRVDSTTQRPSVRLFWREHLHASVDGRELR<br>ESTRTPTSTKWIRERCAQITGRDFVQFVHTHINALPSRIRG<br>SRGRRGGGESSLTCRAGCKVRETTAHILQQCHRTHGGRI<br>LRHNKIVSFVAKAMEENKWTVELEPRLRTSVGLRKPDII<br>ASRDGVGVIVDVQVVSGQRSLDELHREKRNKYGNHGEL<br>VELVAGRLGLPKAECVRATSCTISWRGVWSLTSYKELRS<br>IIGLREPTLQIVPILALRGSHMNWTRFNQMTSVMGGGVG	
**RNA for *in vitro* experiments**		
Gf-full RNA (G. fortiz 3′UTR RNA) (R5 underlined)	UAGGGUAGAUAAUCUUUGUAUAGUGGGGGGGAUCU<br>CAUGUACCGGGUUUCUUUUAUUUGAUUUUCAAUAAA<br>ACAGACGGUAGCUAGGUUCGCAAGGCAGCCACAAGC<br>CAAAGAUAGGUAGGGUGCUCAUAGUGAGUAGGGACA<br>GUGCCUUUUGAUUCACAACGCGUCAAUACCAUCUGA<br>CACGGAUACCCUUACCGGACUUGUCAUGAUCUCCCA<br>GACUUGUCCAAGGUGGACGGGCCACCUUUACUUAAC<br>CCGGAAAAGGAACAUAUAUUAAUUAUAUGUGUUCGG<br>AAAAUAGCC	Cryo-EM structures, Fig. 1, Fig S1-8
Gf-98 RNA (R5 underlined)	GCCGGACUUGUCAUGAUCUCCCAGACUUGUCCAAGG<br>UGGACGGGCCACCUUUACUUAACCCGGAAAAGGAAC<br>AUAUAUUAAUUAUAUGUGUUCGGAAAAUAGCC	Cryo-EM structures, Fig. 1, Fig. S1-8
Pm-full (*P. megacephalum* 3′UTR RNA) (R5 underlined)	UGAGGUUCUAUCCCUCAUGUGCAGAAUUUCCUUUCU<br>AAACCUAUCUCUUAUCCAAACUAUAUACCCGCCCCCC<br>UUUUUCAUGGGAAACUCGUAAUGAUUACAAUAAUUC<br>AUGACCGCUCACUGGACACGGCAACCCUGGUUGGAC<br>GGGCCUCCAGGGGUGUACAUACACUCCGAAUAACUC<br>GAAAAAGAAACCCGCGAGGGUUUUCAAAGUAGCC	Fig. 1, Fig. S1-S2
Pm-112 RNA (Gf-98-like *P. megacephalum* 3′UTR RNA) (R5 underlined)	GUACAAUAAUUCAUGACCGCUCACUGGACACGGCAA<br>CCCUGGUUGGACGGGCCUCCAGGGGUGUACAUACAC<br>UCCGAAUAACUCGAAAAAGAAACCCGCGAGGGUUUU<br>CAAAGUAGCC	Fig. 1, Fig. S1
Bm-full RNA (*B. mori* 3′UTR RNA) (R5 underlined)	GGCCUUGCACAGUAGUCCAGCGGUAAGGGUGUAGAU<br>CAGGCCCGUCUGUUUCUCCCCCGGAGCUCGCUCCCUU<br>GGCUUCCCUUAUAUAUUUUAACAUCAGAAACAGACA<br>UUAAACAUCUACUGAUCCAAUUUCGCCGGCGUACGG<br>CCACGAUCGGGAGGGUGGGAAUCUCGGGGGUCUUCC<br>GAUCCUAAUCCAUGAUGAUUACGACCUGAGUCACUA<br>AAGACGAUGGCAUGAUGAUCCGGCGAUGAAAAUAGC<br>C	Fig.1, Fig. S1
Gf-68 R5 RNA (R5 underlined)	GCAAGGUGGACGGGCCACCUUUACUUAACCCGGAAA<br>AGGAACAUAUAUUAAUUAUAUGUGUUCGGAAAAUAG<br>CC	Cryo-EM structures, Fig. 1, 3-5, Fig. S1, Fig. S7-8
Gf-68 R0 RNA	GCAAGGUGGACGGGCCACCUUUACUUAACCCGGAAA<br>AGGAACAUAUAUUAAUUAUAUGUGUUCGGAAAA	Fig. 4, Fig S8
Gf-68 R4 RNA (R4 underlined)	GCAAGGUGGACGGGCCACCUUUACUUAACCCGGAAA<br>AGGAACAUAUAUUAAUUAUAUGUGUUCGGAAAAUAG<br>C	Fig. 4, Fig. S8
**DNA**		
76 bp R2 target site	TTAGATGACGAGGCATTTGGCTACCTTAAGAGAGTCAT<br>AGTTACTCCCGCCGTTTACCCGCGCTTCACAGCTGAAA<br>(first strand)	Cryo-EM structures, Fig. 1-4, Fig. S3-8, S11

	5′biotin<br>TTTCAGCTGTGAAGCGCGGGTAAACGGCGGGAGTAAC<br>TATGACTCTCTTAAGGTAGCCAAATGCCTCGTCATCTA<br>A (second strand)	
84 bp R2 target site	5′ infrared dye 800CW-<br>ATTCATGCGCGTCACTAATTAGATGACGAGGCATTTGG<br>CTACCTTAAGAGAGTCATAGTTACTCCCGCCGTTTACC<br>CGCGCTTG<br>3′phosphate<br>(first strand)<br>5′cyanine<br>CAAGCGCGGGTAAACGGCGGGAGTAACTATGACTCTC<br>TTAAGGTAGCCAAATGCCTCGTCATCTAATTAGTGACG<br>CGCATGAAT<br>3′phosphate<br>(second strand)	Fig. 1, Fig. 3-5, Fig. S1-S2
Pre- nicked mimic of first strand	Fragment 1<br>5′ infrared dye 800CW-<br>ATTCATGCGCGTCACTAATTAGATGACGAGGCATTTGG<br>CTA - 3′ hydroxyl<br><br>Fragment 2<br>CCTTAAGAGAGTCATAGTTACTCCCGCCGTTTACCCGC<br>GCTTG<br>3′phosphate	Fig. S8
R2 target site after for second strand nicking	TTAGATGACGAGGCATTTGGCTA-<br>TTTTCCGAACACATATAATTAATATATGTTCCTTTTCCG<br>GGTTAAGTAAAGGTGGCCCGTCCACCTTG<br>(pre-nicked first strand with cDNA)<br><br>CCTTAAGAGAGTCATAGTTACTCCCGCCGTTTACCCGC<br>GCTTCACAGCTGAAA (pre-nicked first strand)<br><br>5′biotin<br>TTTCAGCTGTGAAGCGCGGGTAAACGGCGGGAGTAAC<br>TATGACTCTCTTAAGGTAGCCAAATGCCTCGTCATCTA<br>A (second strand)	Cryo-EM structure, Fig. 5, Fig. S9-11
Loading control	GCCCAGAAGGACAAGTTCCCTAAGCCTTGTAATTGG	Fig.1, Fig. 3-5, Fig. S1-S2
**Plasmid sequence for PRINT R2 mRNA IVT**		
TaGu	AGGGGTTCCGCGCACATTTCCCCGAAAAGTGCCACCT<br>GACGTCCCAATGATTAATACGACTCACTATAAGGAAT<br>AAACTAGTATTCTTCTGGTCCCCACAGACTCAGAGAGA<br>ACCCGCCACCATGGAGAAGGTGATGGTCACCGTTCCG<br>GACAAGAACCCTCCATGTCCATGCTGCGGCACCCGGG<br>TGAACAGCGTGCTGAACCTTATCGAGCACCTGAAGGT<br>CAGCCACGGAAAGCGGGGCGTGTGCTTCCGGTGCGCG<br>AAGTGTGGGAAGGAAAATAGCAACTACCACAGCGTGG<br>TGTGCCATTTCCCAAAATGTAGAGGACCTGAGACAGA<br>GAAAGCTCCAGCCGGCGAGTGGATTTGTGAAGTGTGC<br>AACAGAGACTTCACCACCAAAATCGGCCTGGGCCAGC<br>ATAAGAGATTAGCTCACCCCGCTGTTCGGAATCAGGA	Fig. 1, Fig. 2, Fig. 3, Fig. 4, Fig. S2, Fig. S8

	GAGAATCGTCGCCAGCCAGCCTAAGGAAACTAGCAAC<br>AGAGGCGCTCACAAAAGATGCTGGACCAAAGAGGAG<br>GAGGAACTGCTGATCAGACTGGAAGCCCAGTTTGAGG<br>GCAACAAGAACATCAACAAGCTGATCGCAGAACACAT<br>CACCACAAAGACCGCCAAGCAGATCTCCGATAAGAGG<br>AGACTGCTGAGCCGCAAGCCTGCCGAGGAGCCTAGAG<br>AGGAACCTGGCACATGCCACCACACCAGACGGGCAGC<br>TGCCAGCCTGAGAACAGAGCCCGAGATGTCCCACCAC<br>GCTCAGGCCGAAGATCGGGATAATGGCCCTGGCCGGA<br>GACCCCTGCCTGGTAGAGCCGCCGCCGGCGGCAGAAC<br>AATGGACGAGATCAGGAGACACCCTGACAAGGGCAAT<br>GGACAGCAGCGGCCTACCAAACAGAAGTCTGAGGAGC<br>AGCTGCAAGCCTACTACAAAAAGACACTGGAGGAGCG<br>GCTGAGCGCCGGAGCCCTGAATACCTTCCCCCGCGCCT<br>TCAAGCAAGTGATGGAAGGCAGAGATATCAAGCTGGT<br>GATCAACCAGACAGCCCAGGACTGCTTCGGCTGTCTG<br>GAGAGCATCTCTCAGATCAGAACAGCGACCAGAGATA<br>AGAAAGATACCGTGACCAGAGAGAAGCACCCGAAGA<br>AGCCATTCCAAAAGTGGATGAAGGACCGGGCCATAAA<br>GAAGGGCAACTACCTGAGGTTCCAGAGATTGTTCTAC<br>CTGGACCGAGGCAAACTGGCCAAGATCATCCTGGACG<br>ATATCGAATGCCTGAGCTGCGACATCCCCCTGTCTGAG<br>ATCTACAGCGTGTTTAAGACAAGATGGGAAACCACAG<br>GAAGCTTCAAATCTCTGGGCGATTTTAAAACCTACGGC<br>AAGGCCGACAACACTGCCTTCAGAGAACTGATTACAG<br>CTAAGGAAATCGAGAAAAACGTGCAGGAGATGAGCA<br>AGGGCAGCGCCCCTGGCCCTGACGGTATCACCCTGGG<br>AGATGTGGTGAAAATGGACCCCGAGTTCAGCCGCACA<br>ATGGAGATCTTCAACCTGTGGCTGACCACTGGCAAAA<br>TCCCTGACATGGTGCGGGGATGCCGCACCGTGCTGAT<br>ACCTAAGAGCAGCAAGCCAGATAGACTGAAGGATATC<br>AACAACTGGCGGCCTATCACAATCGGCAGCATCCTGC<br>TCCGGCTGTTTAGCAGAATCGTAACCGCCCGTCTGAGT<br>AAGGCCTGTCCTCTGAACCCCAGACAGCGGGGCTTCA<br>TCCGGGCCGCCGGCTGCAGCGAGAACCTCAAACTGCT<br>GCAGACCATCATCTGGTCGGCCAAACGAGAGCACAGA<br>CCTCTCGGCGTGGTGTTCGTGGACATCGCCAAAGCTTT<br>TGATACCGTCTCCCACCAGCACATCATCCACGCCCTGC<br>AGCAGCGGGAAGTGGACCCTCACATCGTGGGACTGGT<br>GTCCAACATGTACGAGAACATCAGCACTTATATCACC<br>ACCAAGAGAAACACCCACACAGACAAGATCCAGATCA<br>GAGTGGGCGTTAAGCAGGGCGATCCTATGTCTCCTCTG<br>CTGTTCAACCTGGCTATGGACCCTCTGCTCTGTAAACT<br>GGAGGAGTCTGGCAAGGGATACCACAGAGGCCAAAG<br>CTCCATCACCGCTATGGCCTTTGCCGACGACCTGGTGC<br>TGCTGTCTGATTCTTGGGAGAACATGAATACCAACATA<br>AGCATCCTGGAAACATTCTGCAACCTTACAGGCCTGA<br>AAACCCAGGGACAAAAGTGCCACGGCTTTTACATCAA<br>GCCTACCAAAGACAGCTACACCATCAACGACTGCGCC<br>GCCTGGACCATCAACGGCACCCCTTTAAACATGATCG<br>ACCCTGGAGAAAGCGAGAAGTATCTGGGCCTGCAGTT<br>CGACCCCTGGATCGGCATCGCTAGAAGCGGCCTGAGC<br>ACAAAGCTGGACTTTTGGCTGCAGAGAATTGACCAGG<br>CCCCTCTGAAGCCTCTGCAGAAAACCGACATCCTGAA<br>AACCTACACAATCCCTCGGCTGATCTACATTGCCGATC<br>ACAGCGAGGTAAAGACCGCCCTGCTGGAAACCCTGGA	

	CCAAAAGATCAGAACCGCCGTGAAGGAATGGCTCCAC<br>CTGCCCCCCTGCACCTGCGACGCTATCCTGTACAGCAG<br>CACCAGGGACGGCGGCCTGGGCATCACCAAGCTGGCG<br>GGCCTGATCCCCTCCGTCCAGGCTAGACGGCTGCATAG<br>AATCGCCCAGAGCAGCGATGACACAATGAAGTGCTTT<br>ATGGAAAAGGAAAAGATGGAACAGCTGCACAAGAAG<br>CTGTGGATTCAGGCCGGTGGCGACAGAGAGAACATTC<br>CTAGCATCTGGGAGGCGCCGCCTAGTAGCGAGCCTCC<br>CAACAACGTGTCTACCAATTCTGAGTGGGAAGCCCCT<br>ACACAGAAGGACAAGTTCCCTAAGCCTTGTAATTGGA<br>GAAAGAACGAGTTCAAGAAGTGGACAAAGCTGGCCTC<br>TCAGGGCCGGGGAATTGTGAATTTCGAGCGGGACAAG<br>ATCAGCAATCACTGGATTCAGTACTACAGAAGAATCC<br>CACACAGAAAGCTGCTGACGGCCCTTCAGCTGAGAGC<br>CAACGTGTACCCCACGCGGGAGTTCCTGGCCAGAGGT<br>AGACAGGACCAGTACATCAAGGCCTGCAGACATTGTG<br>ATGCTGATATCGAGTCTTGCGCCCACATCATCGGCAAC<br>TGCCCTGTGACACAGGACGCGAGAATCAAAAGACACA<br>ACTACATCTGCGAGCTGCTGCTGGAAGAGGCCAAGAA<br>GAAGGACTGGGTGGTGTTCAAGGAACCCCACATCAGA<br>GACAGCAATAAGGAACTCTATAAACCTGACCTGATCT<br>TCGTGAAGGACGCCCGGGCCCTGGTCGTGGACGTGAC<br>CGTGAGATACGAGGCCGCCAAGTCTAGCCTGGAGGAG<br>GCTGCCGCCGAGAAAGTGCGGAAGTACAAGCACCTTG<br>AAACAGAAGTGCGGCACCTGACCAACGCCAAGGACGT<br>CACATTCGTGGGCTTCCCCCTGGGCGCCAGAGGCAAA<br>TGGCACCAGGATAACTTCAAGCTGCTGACAGAGCTGG<br>GCCTGAGCAAGTCCCGCCAAGTGAAGATGGCCGAGAC<br>CTTCAGCACCGTGGCCCTGTTCTCCTCTGTGGACATCG<br>TGCACATGTTCGCTAGCAGAGCCAGAAAGAGCATGGT<br>GATGCATATGGGTGGAGGTAGCGGGGGCAGTGGAGGG<br>ATGGGGAGCGACTACAAAGACCATGACGGTGATTATA<br>AAGATCATGACATCGATTACAAGGATGACGATGACAA<br>GAAGTGATGACCTCGAGCTGGTACTGCATGCACGCAA<br>TGCTAGCTGCCCCTTTCCCGTCCTGGGTACCCCGAGTC<br>TCCCCCGACCTCGGGTCCCAGGTATGCTCCCACCTCCA<br>CCTGCCCCACTCACCACCTCTGCTAGTTCCAGACACCT<br>CCCAAGCACGCAGCAATGCAGCTCAAAACGCTTAGCC<br>TAGCCACACCCCCACGGGAAACAGCAGTGATTAACCT<br>TTAGCAATAAACGAAAGTTTAACTAAGCTATACTAAC<br>CCCAGGGTTGGTCAATTTCGTGCCAGCCACACCCTGGA<br>GCTAGCAAAAAAAAAAAAAAAAAAAAAAAAAAAAAAAAAA<br>AAGTCTTCATCGGAAAGAACATGTGAGCAAAAGGCCA<br>GCAAAAGGCCAGGAACCGTAAAAAGGCCGCGTTGCTG<br>GCGTTTTTCCATAGGCTCCGCCCCCCTGACGAGCATCA<br>CAAAAATCGACGCTCAAGTCAGAGGTGGCGAAACCCG<br>ACAGGACTATAAAGATACCAGGCGTTTCCCCCTGGAA<br>GCTCCCTCGTGCGCTCTCCTGTTCCGACCCTGCCGCTT<br>ACCGGATACCTGTCCGCCTTTCTCCCTTCGGGAAGCGT<br>GGCGCTTTCTCATAGCTCACGCTGTAGGTATCTCAGTT<br>CGGTGTAGGTCGTTCGCTCCAAGCTGGGCTGTGTGCAC<br>GAACCCCCCGTTCAGCCCGACCGCTGCGCCTTATCCGG<br>TAACTATCGTCTTGAGTCCAACCCGGTAAGACACGACT<br>TATCGCCACTGGCAGCAGCCACTGGTAACAGGATTAG<br>CAGAGCGAGGTATGTAGGCGGTGCTACAGAGTTCTTG<br>AAGTGGTGGCCTAACTACGGCTACACTAGAAGAACAG	

	TATTTGGTATCTGCGCTCTGCTGAAGCCAGTTACCTTC<br>GGAAAAAGAGTTGGTAGCTCTTGATCCGGCAAACAAA<br>CCACCGCTGGTAGCGGTGGTTTTTTTGTTTGCAAGCAG<br>CAGATTACGCGCAGAAAAAAAGGATCTCAAGAAGATC<br>CTTTGATCTTTTCTACGGGGTCTGACGCTCAGTGGAAC<br>GAAAACTCACGTTAAGGGATTTTGGTCATGAGATTATC<br>AAAAAGGATCTTCACCTAGATCCTTTTAAATTAAAAAT<br>GAAGTTTTAAATCAATCTAAAGTATATATGAGTAAACT<br>TGGTCTGACAGTTACCAATGCTTAATCAGTGAGGCACC<br>TATCTCAGCGATCTGTCTATTTCGTTCATCCATAGTTGC<br>CTGACTCCCCGTCGTGTAGATAACTACGATACGGGAG<br>GGCTTACCATCTGGCCCCAGTGCTGCAATGATACCGCG<br>AGACCCACGCTCACCGGCTCCAGATTTATCAGCAATA<br>AACCAGCCAGCCGGAAGGGCCGAGCGCAGAAGTGGTC<br>CTGCAACTTTATCCGCCTCCATCCAGTCTATTAATTGTT<br>GCCGGGAAGCTAGAGTAAGTAGTTCGCCAGTTAATAG<br>TTTGCGCAACGTTGTTGCCATTGCTACAGGCATCGTGG<br>TGTCACGCTCGTCGTTTGGTATGGCTTCATTCAGCTCC<br>GGTTCCCAACGATCAAGGCGAGTTACATGATCCCCCAT<br>GTTGTGCAAAAAAGCGGTTAGCTCCTTCGGTCCTCCGA<br>TCGTTGTCAGAAGTAAGTTGGCCGCAGTGTTATCACTC<br>ATGGTTATGGCAGCACTGCATAATTCTCTTACTGTCAT<br>GCCATCCGTAAGATGCTTTTCTGTGACTGGTGAGTACT<br>CAACCAAGTCATTCTGAGAATAGTGTATGCGGCGACC<br>GAGTTGCTCTTGCCCGGCGTCAATACGGGATAATACCG<br>CGCCACATAGCAGAACTTTAAAAGTGCTCATCATTGG<br>AAAACGTTCTTCGGGGCGAAAACTCTCAAGGATCTTA<br>CCGCTGTTGAGATCCAGTTCGATGTAACCCACTCGTGC<br>ACCCAACTGATCTTCAGCATCTTTTACTTTCACCAGCG<br>TTTCTGGGTGAGCAAAAACAGGAAGGCAAAATGCCGC<br>AAAAAAGGGAATAAGGGCGACACGGAAATGTTGAAT<br>ACTCATACTCTTCCTTTTTCAATATTATTGAAGCATTTA<br>TCAGGGTTATTGTCTCATGAGCGGATACATATTTGAAT<br>GTATTTAGAAAAATAAACAAAT	
PlaMe	AGGGGTTCCGCGCACATTTCCCCGAAAAGTGCCACCT<br>GACGTCCCAATGATTAATACGACTCACTATAAGGAAT<br>AAACTAGTATTCTTCTGGTCCCCACAGACTCAGAGAGA<br>ACCCGCCACCATGCAGAAAACCATCATCCAGCTGCCG<br>AATGATAACCCTGCCTGTCCTTTCTGCGGCGATCACGT<br>GGGCAAGCCTTCCGCTCTGAACGTGCACCTGAAGCGC<br>AACCACGGAGGCCGTGAGGTGGAATTCCAGTGTTCTA<br>TGTGCAACAAGGCCGACCCCAAGGCCCACAGCATCCT<br>GTGCCACATCCCTAAGTGTAAAGGAAAGGTGACCGAG<br>GAACCCACCGGCGATTGGGCCTGCGAGACATGTAACA<br>AGCAGTTCAACACCAAGAGCGGCCTGTCCCAGCACAA<br>GAGAATCGCCCATCCCGCTATCCGGAACCAGGAGAGA<br>ATCGCCGCCAGCCAGCCTAAGCCTAACTCTCAAAGAG<br>GAAAGCACAACAGCTGCTGGACGGTGGAAGAAGAAC<br>AGCTGCTGGCCGCTTTCAACAACATGTTCTGGGGCAAG<br>AAAAATATCAATATCCTGATCTCTGATCACATCCACAT<br>GAAAACAGCCAAGCAGATCAGCGAGAAGAGACGGCT<br>GCTGGGACTGAACAAGAACGCGACAGTGACAACCACA<br>AACCCCCTGCCTGTATCCAGCACCTGTCACCTGAAGAT<br>CCGGACCGACTCCCCTAATACCACCACCGGCCTGAAG<br>GATACCTACATGTGCAAGATCAACGAGAACATCGTGA<br>ACCAGGGCCAGATCAAGTTCGATTCTGAGGTTATCAG	Fig. 1, Fig. 3, Fig. 4, Fig. S2

	CGCCTGGATGGCAGGCGACTCTAATATCCGGAGCCTG GTTGAGAGCACTAGCCTGGACATCCTGAGCACATTCCT GATGGAAACACCTAAGCCTAGAAAGAAAGGCAACAA CAAAATCACAAATAAAAAGAGCGGCAAGAAGAAGAA ATGGATGGAAAAGAGAGCCGTGAAAAAAGGATTCTAC AAAAGATACCAACATCTGTTCGAGACAGATAGATGCA AACTGGCAAGCATCATTCTGGATGGCACCGAGCGACT CCAGTGCCAGATTCCTCTGACAGAGATCCTGGAAACA TATAAGTCTAAGTGGGAGACTCTGACTCCATTCGAGG GCCTCGGCCAATTTAAGAGCCACGCCGTGGCCGACAA CACCGCCTTCGAGATTCTGCTGAGCGCCAAGGAAATC ATGAAGAACATCAAGGAGATGAACAAGAACAGCGCC CCAGGCCCTGATAAGGTGAGCCTGAGAGATCTGCTTCT GGCCGACCCCGAATGCAACGCCCTGGAAAAGCTGTTC AACACCTGGCTGATCACCGGAATCATTCCAAACAGCA TAAAAGAATGTAGAAGCCTGCTGATCCCTAAGACGGC GGACCCCGAGGCCCTGAAGGAACTGGGAAATTGGCGG CCTCTGACCATCGGCAGCATCGTGCTGCGGCTGTTTAG CAGAATCATCACCAACAGACTGGCTAAGGCCTGCCCC ATCAACGCCCGGCAGAGAGGCTTCATCGCCACCCCTG GTTGTAGCGAGAACCTGAAGATTCTTCACACAATCGTT AAACAAGCCAAGACCTCCAAAAAGAGCCTGGGAGTGG TGTTCGTGGACATCGCCAAGGCCTTCGACTCAGTGAGC CACGACCACATTATGTGGGTGCTGCAGGAGCGGGGAC TCGACCAGCACATCGTGAACATTATCGAGGACTCTTAT AAGAAGATCCACACCAGAATGGAGGTGGGCACCGAG AGAACCCCCCCTATCGAGATCAAGGTGGGCGTGAAAC AAGGGGACCCCATGAGCCCTCTGCTGTTTAACCTGGCC ATCGACCCTCTGATCACAGCCCTGGAGAAAGCTAATA CCGGCTTTAGCTACGGCAAGAATAAGATCACCTCTCTG GCCTTCGCGGATGACCTGGTGATGCTGTCCGACACCTG GGAGGGCATGAACAAGAATATCCAGATCCTGGAAACA TTTTGCAATCTGAGTGGCCTGAAGGTCCAGGCTAAGA AATGCTACGGCTTCTTCCTGAGCCCTACCCACGATTCA TATACTATCAACAAATGCGACGCCTGGAAGATCGACA AGGACAGCCTGAACATGATCCAGCCTGGAGAATCTGA GAAGTACCTGGGCCTGAAGGTGGACCCTTGGATCGGC TTCAGCAAGCCCGTGCTGGCCGAAAAGCTTACAATCT GGCTGAAGCGGCTGACCGAAGCCCCTCTGAAGCCTAG CCAGAAACTGACAATGCTAAACATCTACACCATCCCG AGAATCATATACCTGGCCGATCACACCGACACCAAGA AAACCCTGCTGAGCAGCCTTGACGACAACATCAGGAC GGTGGTGAAGGGCTGGCTGCACCTGCCTCCTGACACA TGCAACGGCTTCATCTACACCAAAACTCGGGACGGCG GCCTGGGCGTGACCAGACTGGCTTCTCTTATCCCCAGC ATCCAGGCCAGACGGCTGCACCGGATCGCCACCAGCG AGGACGAGACAATCCGGAACATTGCTATGGCCAACAA TATCGAGGAAGAGTTCCAAAACCTGTGGGTGACCGCC GGCGGCAAGAAGGAAGAGATCCCCAGAATCACCGACC CGGTGTCCATCGACTACAGACTGCCAAGACGGATTCT GGAACTGCTGAATGAGTGGGAGAAGCCAGCTCCAAAG AAGATGTACCCCATCCCTTGCAACTGGCGGGAAGCCG AGATGGCCCACTGGAAGAACCTGCCTTGCCAGGGATC AGGCATCGAGCACTTCGACAATGACACCATCTCCAAC GACTGGCTGCAGTTTCACCGGGGCTTCTCCGAGCGACA GTTCCTGATGGGCCTTAAGATCAGAGCCAACGTGTACC	

	CTACCCGGGAGTACCAGGGCAGAGGCAGAACAAACA<br>AGAACGTGAATTGTAGAAATTGCACCGCCTCTTACGA<br>GAGCCTGTCTCATATCCTGGGCCAGTGCCCTGCCGTGC<br>AGGGCGCTAGAATCCGGCGGCACAACAAGCTGTGCAG<br>CATGCTGAAGCGGGAGGCCAAGGAACTGAAGTGGGTC<br>GTGTACGAGGAACCTCACCTACATACAACAGAAAAAG<br>AGCTGAGAAAGCCTGACCTGATCTTCGTGAAGGAGGA<br>AATGGCCCTGGTGGTCGATGTGACAGTGCGGTTTGAGT<br>ACAAGGAAAAGGTGTTCGAGGATGCCGCTGCTGAGAA<br>AGTGCGGCACTACAAGGACCTGACCAGCCAGATCAAG<br>GAGCTGACCGGCGCCAAAGAGATCGAGTACTTCGGCT<br>TCCCCCTGGGCGCCAGAGGAAAGTGGCCTGAGATCAA<br>CGAGAAGGTGCTGACAGCCCTCGGCATGCCTGATTAC<br>CAGCAGAAGCGCACCGCCAAACGGTTCAGCAAGAGAA<br>CCCTGCTGTACAGCATCGACGTGATCAACACCTTTGAG<br>AACATCGGCAAGAACAACAAGAACAACGTCCCCCATA<br>TGGGTGGAGGTAGCGGGGGCAGTGGAGGGATGGGGA<br>GCGACTACAAAGACCATGACGGTGATTATAAAGATCA<br>TGACATCGATTACAAGGATGACGATGACAAGAAGTGA<br>TGACCTCGAGCTGGTACTGCATGCACGCAATGCTAGCT<br>GCCCCTTTCCCGTCCTGGGTACCCCGAGTCTCCCCCGA<br>CCTCGGGTCCCAGGTATGCTCCCACCTCCACCTGCCCC<br>ACTCACCACCTCTGCTAGTTCCAGACACCTCCCAAGCA<br>CGCAGCAATGCAGCTCAAAACGCTTAGCCTAGCCACA<br>CCCCCACGGGAAACAGCAGTGATTAACCTTTAGCAAT<br>AAACGAAAGTTTAACTAAGCTATACTAACCCCAGGGT<br>TGGTCAATTTCGTGCCAGCCACACCCTGGAGCTAGCAA<br>AAAAAAAAAAAAAAAAAAAAAAAAAAAAAAGTCTTC<br>ATCGGAAAGAACATGTGAGCAAAAGGCCAGCAAAAG<br>GCCAGGAACCGTAAAAAGGCCGCGTTGCTGGCGTTTT<br>TCCATAGGCTCCGCCCCCCTGACGAGCATCACAAAAA<br>TCGACGCTCAAGTCAGAGGTGGCGAAACCCGACAGGA<br>CTATAAAGATACCAGGCGTTTCCCCCTGGAAGCTCCCT<br>CGTGCGCTCTCCTGTTCCGACCCTGCCGCTTACCGGAT<br>ACCTGTCCGCCTTTCTCCCTTCGGGAAGCGTGGCGCTT<br>TCTCATAGCTCACGCTGTAGGTATCTCAGTTCGGTGTA<br>GGTCGTTCGCTCCAAGCTGGGCTGTGTGCACGAACCCC<br>CCGTTCAGCCCGACCGCTGCGCCTTATCCGGTAACTAT<br>CGTCTTGAGTCCAACCCGGTAAGACACGACTTATCGCC<br>ACTGGCAGCAGCCACTGGTAACAGGATTAGCAGAGCG<br>AGGTATGTAGGCGGTGCTACAGAGTTCTTGAAGTGGT<br>GGCCTAACTACGGCTACACTAGAAGAACAGTATTTGG<br>TATCTGCGCTCTGCTGAAGCCAGTTACCTTCGGAAAAA<br>GAGTTGGTAGCTCTTGATCCGGCAAACAAACCACCGC<br>TGGTAGCGGTGGTTTTTTTGTTTGCAAGCAGCAGATTA<br>CGCGCAGAAAAAAAGGATCTCAAGAAGATCCTTTGAT<br>CTTTTCTACGGGGTCTGACGCTCAGTGGAACGAAAACT<br>CACGTTAAGGGATTTTGGTCATGAGATTATCAAAAAG<br>GATCTTCACCTAGATCCTTTTAAATTAAAAATGAAGTT<br>TTAAATCAATCTAAAGTATATATGAGTAAACTTGGTCT<br>GACAGTTACCAATGCTTAATCAGTGAGGCACCTATCTC<br>AGCGATCTGTCTATTTCGTTCATCCATAGTTGCCTGAC<br>TCCCCGTCGTGTAGATAACTACGATACGGGAGGGCTT<br>ACCATCTGGCCCCAGTGCTGCAATGATACCGCGAGAC<br>CCACGCTCACCGGCTCCAGATTTATCAGCAATAAACCA<br>GCCAGCCGGAAGGGCCGAGCGCAGAAGTGGTCCTGCA	

	ACTTTATCCGCCTCCATCCAGTCTATTAATTGTTGCCG<br>GGAAGCTAGAGTAAGTAGTTCGCCAGTTAATAGTTTG<br>CGCAACGTTGTTGCCATTGCTACAGGCATCGTGGTGTC<br>ACGCTCGTCGTTTGGTATGGCTTCATTCAGCTCCGGTT<br>CCCAACGATCAAGGCGAGTTACATGATCCCCCATGTTG<br>TGCAAAAAAGCGGTTAGCTCCTTCGGTCCTCCGATCGT<br>TGTCAGAAGTAAGTTGGCCGCAGTGTTATCACTCATGG<br>TTATGGCAGCACTGCATAATTCTCTTACTGTCATGCCA<br>TCCGTAAGATGCTTTTCTGTGACTGGTGAGTACTCAAC<br>CAAGTCATTCTGAGAATAGTGTATGCGGCGACCGAGT<br>TGCTCTTGCCCGGCGTCAATACGGGATAATACCGCGCC<br>ACATAGCAGAACTTTAAAAGTGCTCATCATTGGAAAA<br>CGTTCTTCGGGGCGAAAACTCTCAAGGATCTTACCGCT<br>GTTGAGATCCAGTTCGATGTAACCCACTCGTGCACCCA<br>ACTGATCTTCAGCATCTTTTACTTTCACCAGCGTTTCTG<br>GGTGAGCAAAAACAGGAAGGCAAAATGCCGCAAAAA<br>AGGGAATAAGGGCGACACGGAAATGTTGAATACTCAT<br>ACTCTTCCTTTTTCAATATTATTGAAGCATTTATCAGGG<br>TTATTGTCTCATGAGCGGATACATATTTGAATGTATTT<br>AGAAAAATAAACAAAT	
TaGu RT-dead	AGGGGTTCCGCGCACATTTCCCCGAAAAGTGCCACCT<br>GACGTCCCAATGATTAATACGACTCACTATAAGGAAT<br>AAACTAGTATTCTTCTGGTCCCCACAGACTCAGAGAGA<br>ACCCGCCACCATGGAGAAGGTGATGGTCACCGTTCCG<br>GACAAGAACCCTCCATGTCCATGCTGCGGCACCCGGG<br>TGAACAGCGTGCTGAACCTTATCGAGCACCTGAAGGT<br>CAGCCACGGAAAGCGGGGCGTGTGCTTCCGGTGCGCG<br>AAGTGTGGGAAGGAAAATAGCAACTACCACAGCGTGG<br>TGTGCCATTTCCCAAAATGTAGAGGACCTGAGACAGA<br>GAAAGCTCCAGCCGGCGAGTGGATTTGTGAAGTGTGC<br>AACAGAGACTTCACCACCAAAATCGGCCTGGGCCAGC<br>ATAAGAGATTAGCTCACCCCGCTGTTCGGAATCAGGA<br>GAGAATCGTCGCCAGCCAGCCTAAGGAAACTAGCAAC<br>AGAGGCGCTCACAAAAGATGCTGGACCAAAGAGGAG<br>GAGGAACTGCTGATCAGACTGGAAGCCCAGTTTGAGG<br>GCAACAAGAACATCAACAAGCTGATCGCAGAACACAT<br>CACCACAAAGACCGCCAAGCAGATCTCCGATAAGAGG<br>AGACTGCTGAGCCGCAAGCCTGCCGAGGAGCCTAGAG<br>AGGAACCTGGCACATGCCACCACACCAGACGGGCAGC<br>TGCCAGCCTGAGAACAGAGCCCGAGATGTCCCACCAC<br>GCTCAGGCCGAAGATCGGGATAATGGCCCTGGCCGGA<br>GACCCCTGCCTGGTAGAGCCGCCGCCGGCGGCAGAAC<br>AATGGACGAGATCAGGAGACACCCTGACAAGGGCAAT<br>GGACAGCAGCGGCCTACCAAACAGAAGTCTGAGGAGC<br>AGCTGCAAGCCTACTACAAAAAGACACTGGAGGAGCG<br>GCTGAGCGCCGGAGCCCTGAATACCTTCCCCCGCGCCT<br>TCAAGCAAGTGATGGAAGGCAGAGATATCAAGCTGGT<br>GATCAACCAGACAGCCCAGGACTGCTTCGGCTGTCTG<br>GAGAGCATCTCTCAGATCAGAACAGCGACCAGAGATA<br>AGAAAGATACCGTGACCAGAGAGAAGCACCCGAAGA<br>AGCCATTCCAAAAGTGGATGAAGGACCGGGCCATAAA<br>GAAGGGCAACTACCTGAGGTTCCAGAGATTGTTCTAC<br>CTGGACCGAGGCAAACTGGCCAAGATCATCCTGGACG<br>ATATCGAATGCCTGAGCTGCGACATCCCCCTGTCTGAG<br>ATCTACAGCGTGTTTAAGACAAGATGGGAAACCACAG<br>GAAGCTTCAAATCTCTGGGCGATTTTAAAACCTACGGC	Fig. S2

	AAGGCCGACAACACTGCCTTCAGAGAACTGATTACAG<br>CTAAGGAAATCGAGAAAAACGTGCAGGAGATGAGCA<br>AGGGCAGCGCCCCTGGCCCTGACGGTATCACCCTGGG<br>AGATGTGGTGAAAATGGACCCCGAGTTCAGCCGCACA<br>ATGGAGATCTTCAACCTGTGGCTGACCACTGGCAAAA<br>TCCCTGACATGGTGCGGGGATGCCGCACCGTGCTGAT<br>ACCTAAGAGCAGCAAGCCAGATAGACTGAAGGATATC<br>AACAACTGGCGGCCTATCACAATCGGCAGCATCCTGC<br>TCCGGCTGTTTAGCAGAATCGTAACCGCCCGTCTGAGT<br>AAGGCCTGTCCTCTGAACCCCAGACAGCGGGGCTTCA<br>TCCGGGCCGCCGGCTGCAGCGAGAACCTCAAACTGCT<br>GCAGACCATCATCTGGTCGGCCAAACGAGAGCACAGA<br>CCTCTCGGCGTGGTGTTCGTGGACATCGCCAAAGCTTT<br>TGATACCGTCTCCCACCAGCACATCATCCACGCCCTGC<br>AGCAGCGGGAAGTGGACCCTCACATCGTGGGACTGGT<br>GTCCAACATGTACGAGAACATCAGCACTTATATCACC<br>ACCAAGAGAAACACCCACACAGACAAGATCCAGATCA<br>GAGTGGGCGTTAAGCAGGGCGATCCTATGTCTCCTCTG<br>CTGTTCAACCTGGCTATGGACCCTCTGCTCTGTAAACT<br>GGAGGAGTCTGGCAAGGGATACCACAGAGGCCAAAG<br>CTCCATCACCGCTATGGCCTTTGCCGCCGCACTGGTGC<br>TGCTGTCTGATTCTTGGGAGAACATGAATACCAACATA<br>AGCATCCTGGAAACATTCTGCAACCTTACAGGCCTGA<br>AAACCCAGGGACAAAAGTGCCACGGCTTTTACATCAA<br>GCCTACCAAAGACAGCTACACCATCAACGACTGCGCC<br>GCCTGGACCATCAACGGCACCCCTTTAAACATGATCG<br>ACCCTGGAGAAAGCGAGAAGTATCTGGGCCTGCAGTT<br>CGACCCCTGGATCGGCATCGCTAGAAGCGGCCTGAGC<br>ACAAAGCTGGACTTTTGGCTGCAGAGAATTGACCAGG<br>CCCCTCTGAAGCCTCTGCAGAAAACCGACATCCTGAA<br>AACCTACACAATCCCTCGGCTGATCTACATTGCCGATC<br>ACAGCGAGGTAAAGACCGCCCTGCTGGAAACCCTGGA<br>CCAAAAGATCAGAACCGCCGTGAAGGAATGGCTCCAC<br>CTGCCCCCCTGCACCTGCGACGCTATCCTGTACAGCAG<br>CACCAGGGACGGCGGCCTGGGCATCACCAAGCTGGCG<br>GGCCTGATCCCCTCCGTCCAGGCTAGACGGCTGCATAG<br>AATCGCCCAGAGCAGCGATGACACAATGAAGTGCTTT<br>ATGGAAAAGGAAAAGATGGAACAGCTGCACAAGAAG<br>CTGTGGATTCAGGCCGGTGGCGACAGAGAGAACATTC<br>CTAGCATCTGGGAGGCGCCGCCTAGTAGCGAGCCTCC<br>CAACAACGTGTCTACCAATTCTGAGTGGGAAGCCCCT<br>ACACAGAAGGACAAGTTCCCTAAGCCTTGTAATTGGA<br>GAAAGAACGAGTTCAAGAAGTGGACAAAGCTGGCCTC<br>TCAGGGCCGGGGAATTGTGAATTTCGAGCGGGACAAG<br>ATCAGCAATCACTGGATTCAGTACTACAGAAGAATCC<br>CACACAGAAAGCTGCTGACGGCCCTTCAGCTGAGAGC<br>CAACGTGTACCCCACGCGGGAGTTCCTGGCCAGAGGT<br>AGACAGGACCAGTACATCAAGGCCTGCAGACATTGTG<br>ATGCTGATATCGAGTCTTGCGCCCACATCATCGGCAAC<br>TGCCCTGTGACACAGGACGCGAGAATCAAAAGACACA<br>ACTACATCTGCGAGCTGCTGCTGGAAGAGGCCAAGAA<br>GAAGGACTGGGTGGTGTTCAAGGAACCCCACATCAGA<br>GACAGCAATAAGGAACTCTATAAACCTGACCTGATCT<br>TCGTGAAGGACGCCCGGGCCCTGGTCGTGGACGTGAC<br>CGTGAGATACGAGGCCGCCAAGTCTAGCCTGGAGGAG<br>GCTGCCGCCGAGAAAGTGCGGAAGTACAAGCACCTTG	

	AAACAGAAGTGCGGCACCTGACCAACGCCAAGGACGT<br>CACATTCGTGGGCTTCCCCCTGGGCGCCAGAGGCAAA<br>TGGCACCAGGATAACTTCAAGCTGCTGACAGAGCTGG<br>GCCTGAGCAAGTCCCGCCAAGTGAAGATGGCCGAGAC<br>CTTCAGCACCGTGGCCCTGTTCTCCTCTGTGGACATCG<br>TGCACATGTTCGCTAGCAGAGCCAGAAAGAGCATGGT<br>GATGCATATGGGTGGAGGTAGCGGGGGCAGTGGAGGG<br>ATGGGGAGCGACTACAAAGACCATGACGGTGATTATA<br>AAGATCATGACATCGATTACAAGGATGACGATGACAA<br>GAAGTGATGACCTCGAGCTGGTACTGCATGCACGCAA<br>TGCTAGCTGCCCCTTTCCCGTCCTGGGTACCCCGAGTC<br>TCCCCCGACCTCGGGTCCCAGGTATGCTCCCACCTCCA<br>CCTGCCCCACTCACCACCTCTGCTAGTTCCAGACACCT<br>CCCAAGCACGCAGCAATGCAGCTCAAAACGCTTAGCC<br>TAGCCACACCCCCACGGGAAACAGCAGTGATTAACCT<br>TTAGCAATAAACGAAAGTTTAACTAAGCTATACTAAC<br>CCCAGGGTTGGTCAATTTCGTGCCAGCCACACCCTGGA<br>GCTAGCAAAAAAAAAAAAAAAAAAAAAAAAAAAAAAA<br>AAGTCTTCATCGGAAAGAACATGTGAGCAAAAGGCCA<br>GCAAAAGGCCAGGAACCGTAAAAAGGCCGCGTTGCTG<br>GCGTTTTTCCATAGGCTCCGCCCCCCTGACGAGCATCA<br>CAAAAATCGACGCTCAAGTCAGAGGTGGCGAAACCCG<br>ACAGGACTATAAAGATACCAGGCGTTTCCCCCTGGAA<br>GCTCCCTCGTGCGCTCTCCTGTTCCGACCCTGCCGCTT<br>ACCGGATACCTGTCCGCCTTTCTCCCTTCGGGAAGCGT<br>GGCGCTTTCTCATAGCTCACGCTGTAGGTATCTCAGTT<br>CGGTGTAGGTCGTTCGCTCCAAGCTGGGCTGTGTGCAC<br>GAACCCCCCGTTCAGCCCGACCGCTGCGCCTTATCCGG<br>TAACTATCGTCTTGAGTCCAACCCGGTAAGACACGACT<br>TATCGCCACTGGCAGCAGCCACTGGTAACAGGATTAG<br>CAGAGCGAGGTATGTAGGCGGTGCTACAGAGTTCTTG<br>AAGTGGTGGCCTAACTACGGCTACACTAGAAGAACAG<br>TATTTGGTATCTGCGCTCTGCTGAAGCCAGTTACCTTC<br>GGAAAAAGAGTTGGTAGCTCTTGATCCGGCAAACAAA<br>CCACCGCTGGTAGCGGTGGTTTTTTTGTTTGCAAGCAG<br>CAGATTACGCGCAGAAAAAAAGGATCTCAAGAAGATC<br>CTTTGATCTTTTCTACGGGGTCTGACGCTCAGTGGAAC<br>GAAAACTCACGTTAAGGGATTTTGGTCATGAGATTATC<br>AAAAAGGATCTTCACCTAGATCCTTTTAAATTAAAAAT<br>GAAGTTTTAAATCAATCTAAAGTATATATGAGTAAACT<br>TGGTCTGACAGTTACCAATGCTTAATCAGTGAGGCACC<br>TATCTCAGCGATCTGTCTATTTCGTTCATCCATAGTTGC<br>CTGACTCCCCGTCGTGTAGATAACTACGATACGGGAG<br>GGCTTACCATCTGGCCCCAGTGCTGCAATGATACCGCG<br>AGACCCACGCTCACCGGCTCCAGATTTATCAGCAATA<br>AACCAGCCAGCCGGAAGGGCCGAGCGCAGAAGTGGTC<br>CTGCAACTTTATCCGCCTCCATCCAGTCTATTAATTGTT<br>GCCGGGAAGCTAGAGTAAGTAGTTCGCCAGTTAATAG<br>TTTGCGCAACGTTGTTGCCATTGCTACAGGCATCGTGG<br>TGTCACGCTCGTCGTTTGGTATGGCTTCATTCAGCTCC<br>GGTTCCCAACGATCAAGGCGAGTTACATGATCCCCCAT<br>GTTGTGCAAAAAAGCGGTTAGCTCCTTCGGTCCTCCGA<br>TCGTTGTCAGAAGTAAGTTGGCCGCAGTGTTATCACTC<br>ATGGTTATGGCAGCACTGCATAATTCTCTTACTGTCAT<br>GCCATCCGTAAGATGCTTTTCTGTGACTGGTGAGTACT<br>CAACCAAGTCATTCTGAGAATAGTGTATGCGGCGACC	

	GAGTTGCTCTTGCCCGGCGTCAATACGGGATAATACCG<br>CGCCACATAGCAGAACTTTAAAAGTGCTCATCATTGG<br>AAAACGTTCTTCGGGGCGAAAACTCTCAAGGATCTTA<br>CCGCTGTTGAGATCCAGTTCGATGTAACCCACTCGTGC<br>ACCCAACTGATCTTCAGCATCTTTTACTTTCACCAGCG<br>TTTCTGGGTGAGCAAAAACAGGAAGGCAAAATGCCGC<br>AAAAAAGGGAATAAGGGCGACACGGAAATGTTGAAT<br>ACTCATACTCTTCCTTTTTCAATATTATTGAAGCATTTA<br>TCAGGGTTATTGTCTCATGAGCGGATACATATTTGAAT<br>GTATTTAGAAAAATAAACAAAT	
PlaMe NZa	AGGGGTTCCGCGCACATTTCCCCGAAAAGTGCCACCT<br>GACGTCCCAATGATTAATACGACTCACTATAAGGAAT<br>AAACTAGTATTCTTCTGGTCCCCACAGACTCAGAGAGA<br>ACCCGCCACCATGGATAACATCGTGAAGGTGACCGTG<br>CCCGACAAGAATCCCCCTTGTCCTTGCTGTGGCGTGCG<br>CCTGAACAGCGTGCTGGCACTGATCGAGCACCTGAAG<br>GGCTCCCACGGAAGGAGAAGGGTGTGCTTCCGCTGTG<br>CCAAGTGCGGCCGGGAGAACTTTAATCACCACAGCAC<br>CGTGTGTCACTACGCCAAGTGCAAGGGCCCACAGATC<br>GAGAGACCACCCGTGGGCGAGTGGATCTGTGAGGTGT<br>GCGGCAGGGATTTCACCACAAAGATCGGCCTGGGCCA<br>GCACAAGCGCCACATGCACGCCATGGTGCGCAACCAG<br>GAGCGGATCGACGCCAGCCAGCCCAAGGAGACCTCCA<br>ATCGCGGCGCCCACAAGCGGTGCTGGACAAAGGAGGA<br>GGAGGAGCTGCTGATGAAGCTGGAGGTGCAGTTTGAG<br>AACCACAAGAACATCAATAAGCTGATCGCCGAGCAGC<br>TGACCACAAAGACCGCCAAGCAGATCTCCGATAAGCG<br>CCGGATGCTGCTGAAGAAGGGCAGAGGCACCACAGGC<br>AACCTGGAGACAGAGCCTGGCATGAGCCACCAGTCCC<br>AGGCCAAGGTGAAGGACAATGGACTGGGAGGCGATC<br>ACCTGCCAGGAGGACCTGTGGTGGACAAGGGCACCAT<br>CGGCAAGCCAGGCCAGCACCTGGACACCGATAATTCT<br>CACCAGATCACAGCAGGCAAGAAGAAGGGAGGAGGA<br>CTGAAGGATACCTACATGTGCAAGATCAACGAGAACA<br>TCGTGAACCAGGGCCAGATCAAGTTCGATTCTGAGGTT<br>ATCAGCGCCTGGATGGCAGGCGACTCTAATATCCGGA<br>GCCTGGTTGAGAGCACTAGCCTGGACATCCTGAGCAC<br>ATTCCTGATGGAAACACCTAAGCCTAGAAAGAAAGGC<br>AACAACAAAATCACAAATAAAAAGAGCGGCAAGAAG<br>AAGAAATGGATGGAAAAGAGAGCCGTGAAAAAAGGA<br>TTCTACAAAAGATACCAACATCTGTTCGAGACAGATA<br>GATGCAAACTGGCAAGCATCATTCTGGATGGCACCGA<br>GCGACTCCAGTGCCAGATTCCTCTGACAGAGATCCTGG<br>AAACATATAAGTCTAAGTGGGAGACTCTGACTCCATTC<br>GAGGGCCTCGGCCAATTTAAGAGCCACGCCGTGGCCG<br>ACAACACCGCCTTCGAGATTCTGCTGAGCGCCAAGGA<br>AATCATGAAGAACATCAAGGAGATGAACAAGAACAG<br>CGCCCCAGGCCCTGATAAGGTGAGCCTGAGAGATCTG<br>CTTCTGGCCGACCCCGAATGCAACGCCCTGGAAAAGC<br>TGTTCAACACCTGGCTGATCACCGGAATCATTCCAAAC<br>AGCATAAAAGAATGTAGAAGCCTGCTGATCCCTAAGA<br>CGGCGGACCCCGAGGCCCTGAAGGAACTGGGAAATTG<br>GCGGCCTCTGACCATCGGCAGCATCGTGCTGCGGCTGT<br>TTAGCAGAATCATCACCAACAGACTGGCTAAGGCCTG<br>CCCCATCAACGCCCGGCAGAGAGGCTTCATCGCCACC<br>CCTGGTTGTAGCGAGAACCTGAAGATTCTTCACACAAT	Fig. 3

	CGTTAAACAAGCCAAGACCTCCAAAAAGAGCCTGGGA<br>GTGGTGTTCGTGGACATCGCCAAGGCCTTCGACTCAGT<br>GAGCCACGACCACATTATGTGGGTGCTGCAGGAGCGG<br>GGACTCGACCAGCACATCGTGAACATTATCGAGGACT<br>CTTATAAGAAGATCCACACCAGAATGGAGGTGGGCAC<br>CGAGAGAACCCCCCCTATCGAGATCAAGGTGGGCGTG<br>AAACAAGGGGACCCCATGAGCCCTCTGCTGTTTAACCT<br>GGCCATCGACCCTCTGATCACAGCCCTGGAGAAAGCT<br>AATACCGGCTTTAGCTACGGCAAGAATAAGATCACCT<br>CTCTGGCCTTCGCGGATGACCTGGTGATGCTGTCCGAC<br>ACCTGGGAGGGCATGAACAAGAATATCCAGATCCTGG<br>AAACATTTTGCAATCTGAGTGGCCTGAAGGTCCAGGCT<br>AAGAAATGCTACGGCTTCTTCCTGAGCCCTACCCACGA<br>TTCATATACTATCAACAAATGCGACGCCTGGAAGATC<br>GACAAGGACAGCCTGAACATGATCCAGCCTGGAGAAT<br>CTGAGAAGTACCTGGGCCTGAAGGTGGACCCTTGGAT<br>CGGCTTCAGCAAGCCCGTGCTGGCCGAAAAGCTTACA<br>ATCTGGCTGAAGCGGCTGACCGAAGCCCCTCTGAAGC<br>CTAGCCAGAAACTGACAATGCTAAACATCTACACCAT<br>CCCGAGAATCATATACCTGGCCGATCACACCGACACC<br>AAGAAAACCCTGCTGAGCAGCCTTGACGACAACATCA<br>GGACGGTGGTGAAGGGCTGGCTGCACCTGCCTCCTGA<br>CACATGCAACGGCTTCATCTACACCAAAACTCGGGAC<br>GGCGGCCTGGGCGTGACCAGACTGGCTTCTCTTATCCC<br>CAGCATCCAGGCCAGACGGCTGCACCGGATCGCCACC<br>AGCGAGGACGAGACAATCCGGAACATTGCTATGGCCA<br>ACAATATCGAGGAAGAGTTCCAAAACCTGTGGGTGAC<br>CGCCGGCGGCAAGAAGGAAGAGATCCCCAGAATCACC<br>GACCCGGTGTCCATCGACTACAGACTGCCAAGACGGA<br>TTCTGGAACTGCTGAATGAGTGGGAGAAGCCAGCTCC<br>AAAGAAGATGTACCCCATCCCTTGCAACTGGCGGGAA<br>GCCGAGATGGCCCACTGGAAGAACCTGCCTTGCCAGG<br>GATCAGGCATCGAGCACTTCGACAATGACACCATCTC<br>CAACGACTGGCTGCAGTTTCACCGGGGCTTCTCCGAGC<br>GACAGTTCCTGATGGGCCTTAAGATCAGAGCCAACGT<br>GTACCCTACCCGGGAGTACCAGGGCAGAGGCAGAACA<br>AACAAGAACGTGAATTGTAGAAATTGCACCGCCTCTT<br>ACGAGAGCCTGTCTCATATCCTGGGCCAGTGCCCTGCC<br>GTGCAGGGCGCTAGAATCCGGCGGCACAACAAGCTGT<br>GCAGCATGCTGAAGCGGGAGGCCAAGGAACTGAAGTG<br>GGTCGTGTACGAGGAACCTCACCTACATACAACAGAA<br>AAAGAGCTGAGAAAGCCTGACCTGATCTTCGTGAAGG<br>AGGAAATGGCCCTGGTGGTCGATGTGACAGTGCGGTT<br>TGAGTACAAGGAAAAGGTGTTCGAGGATGCCGCTGCT<br>GAGAAAGTGCGGCACTACAAGGACCTGACCAGCCAGA<br>TCAAGGAGCTGACCGGCGCCAAAGAGATCGAGTACTT<br>CGGCTTCCCCCTGGGCGCCAGAGGAAAGTGGCCTGAG<br>ATCAACGAGAAGGTGCTGACAGCCCTCGGCATGCCTG<br>ATTACCAGCAGAAGCGCACCGCCAAACGGTTCAGCAA<br>GAGAACCCTGCTGTACAGCATCGACGTGATCAACACC<br>TTTGAGAACATCGGCAAGAACAACAAGAACAACGTCC<br>CCCATATGGGTGGAGGTAGCGGGGGCAGTGGAGGGAT<br>GGGGAGCGACTACAAAGACCATGACGGTGATTATAAA<br>GATCATGACATCGATTACAAGGATGACGATGACAAGA<br>AGTGATGACCTCGAGCTGGTACTGCATGCACGCAATG<br>CTAGCTGCCCCTTTCCCGTCCTGGGTACCCCGAGTCTC	

	CCCCGACCTCGGGTCCCAGGTATGCTCCCACCTCCACC<br>TGCCCCACTCACCACCTCTGCTAGTTCCAGACACCTCC<br>CAAGCACGCAGCAATGCAGCTCAAAACGCTTAGCCTA<br>GCCACACCCCCACGGGAAACAGCAGTGATTAACCTTT<br>AGCAATAAACGAAAGTTTAACTAAGCTATACTAACCC<br>CAGGGTTGGTCAATTTCGTGCCAGCCACACCCTGGAGC<br>TAGCAAAAAAAAAAAAAAAAAAAAAAAAAAAAAAAA<br>GTCTTCATCGGAAAGAACATGTGAGCAAAAGGCCAGC<br>AAAAGGCCAGGAACCGTAAAAAGGCCGCGTTGCTGGC<br>GTTTTTCCATAGGCTCCGCCCCCCTGACGAGCATCACA<br>AAAATCGACGCTCAAGTCAGAGGTGGCGAAACCCGAC<br>AGGACTATAAAGATACCAGGCGTTTCCCCCTGGAAGC<br>TCCCTCGTGCGCTCTCCTGTTCCGACCCTGCCGCTTACC<br>GGATACCTGTCCGCCTTTCTCCCTTCGGGAAGCGTGGC<br>GCTTTCTCATAGCTCACGCTGTAGGTATCTCAGTTCGG<br>TGTAGGTCGTTCGCTCCAAGCTGGGCTGTGTGCACGAA<br>CCCCCCGTTCAGCCCGACCGCTGCGCCTTATCCGGTAA<br>CTATCGTCTTGAGTCCAACCCGGTAAGACACGACTTAT<br>CGCCACTGGCAGCAGCCACTGGTAACAGGATTAGCAG<br>AGCGAGGTATGTAGGCGGTGCTACAGAGTTCTTGAAG<br>TGGTGGCCTAACTACGGCTACACTAGAAGAACAGTAT<br>TTGGTATCTGCGCTCTGCTGAAGCCAGTTACCTTCGGA<br>AAAAGAGTTGGTAGCTCTTGATCCGGCAAACAAACCA<br>CCGCTGGTAGCGGTGGTTTTTTTGTTTGCAAGCAGCAG<br>ATTACGCGCAGAAAAAAAGGATCTCAAGAAGATCCTT<br>TGATCTTTTCTACGGGGTCTGACGCTCAGTGGAACGAA<br>AACTCACGTTAAGGGATTTTGGTCATGAGATTATCAAA<br>AAGGATCTTCACCTAGATCCTTTTAAATTAAAAATGAA<br>GTTTTAAATCAATCTAAAGTATATATGAGTAAACTTGG<br>TCTGACAGTTACCAATGCTTAATCAGTGAGGCACCTAT<br>CTCAGCGATCTGTCTATTTCGTTCATCCATAGTTGCCTG<br>ACTCCCCGTCGTGTAGATAACTACGATACGGGAGGGC<br>TTACCATCTGGCCCCAGTGCTGCAATGATACCGCGAGA<br>CCCACGCTCACCGGCTCCAGATTTATCAGCAATAAACC<br>AGCCAGCCGGAAGGGCCGAGCGCAGAAGTGGTCCTGC<br>AACTTTATCCGCCTCCATCCAGTCTATTAATTGTTGCC<br>GGGAAGCTAGAGTAAGTAGTTCGCCAGTTAATAGTTT<br>GCGCAACGTTGTTGCCATTGCTACAGGCATCGTGGTGT<br>CACGCTCGTCGTTTGGTATGGCTTCATTCAGCTCCGGT<br>TCCCAACGATCAAGGCGAGTTACATGATCCCCCATGTT<br>GTGCAAAAAAGCGGTTAGCTCCTTCGGTCCTCCGATCG<br>TTGTCAGAAGTAAGTTGGCCGCAGTGTTATCACTCATG<br>GTTATGGCAGCACTGCATAATTCTCTTACTGTCATGCC<br>ATCCGTAAGATGCTTTTCTGTGACTGGTGAGTACTCAA<br>CCAAGTCATTCTGAGAATAGTGTATGCGGCGACCGAG<br>TTGCTCTTGCCCGGCGTCAATACGGGATAATACCGCGC<br>CACATAGCAGAACTTTAAAAGTGCTCATCATTGGAAA<br>ACGTTCTTCGGGGCGAAAACTCTCAAGGATCTTACCGC<br>TGTTGAGATCCAGTTCGATGTAACCCACTCGTGCACCC<br>AACTGATCTTCAGCATCTTTTACTTTCACCAGCGTTTCT<br>GGGTGAGCAAAAACAGGAAGGCAAAATGCCGCAAAA<br>AAGGGAATAAGGGCGACACGGAAATGTTGAATACTCA<br>TACTCTTCCTTTTTCAATATTATTGAAGCATTTATCAGG<br>GTTATTGTCTCATGAGCGGATACATATTTGAATGTATT<br>TAGAAAAATAAACAAAT	

PlaMe NTg	AGGGGTTCCGCGCACATTTCCCCGAAAAGTGCCACCT<br>GACGTCCCAATGATTAATACGACTCACTATAAGGAAT<br>AAACTAGTATTCTTCTGGTCCCCACAGACTCAGAGAGA<br>ACCCGCCACCATGGAGAAGGTGATGGTCACCGTTCCG<br>GACAAGAACCCTCCATGTCCATGCTGCGGCACCCGGG<br>TGAACAGCGTGCTGAACCTTATCGAGCACCTGAAGGT<br>CAGCCACGGAAAGCGGGGCGTGTGCTTCCGGTGCGCG<br>AAGTGTGGGAAGGAAAATAGCAACTACCACAGCGTGG<br>TGTGCCATTTCCCAAAATGTAGAGGACCTGAGACAGA<br>GAAAGCTCCAGCCGGCGAGTGGATTTGTGAAGTGTGC<br>AACAGAGACTTCACCACCAAAATCGGCCTGGGCCAGC<br>ATAAGAGATTAGCTCACCCCGCTGTTCGGAATCAGGA<br>GAGAATCGTCGCCAGCCAGCCTAAGGAAACTAGCAAC<br>AGAGGCGCTCACAAAAGATGCTGGACCAAAGAGGAG<br>GAGGAACTGCTGATCAGACTGGAAGCCCAGTTTGAGG<br>GCAACAAGAACATCAACAAGCTGATCGCAGAACACAT<br>CACCACAAAGACCGCCAAGCAGATCTCCGATAAGAGG<br>AGACTGCTGAGCCGCAAGCCTGCCGAGGAGCCTAGAG<br>AGGAACCTGGCACATGCCACCACACCAGACGGGCAGC<br>TGCCAGCCTGAGAACAGAGCCCGAGATGTCCCACCAC<br>GCTCAGGCCGAAGATCGGGATAATGGCCCTGGCCGGA<br>GACCCCTGCCTGGTAGAGCCGCCGCCGGCGGCAGAAC<br>AATGGACGAGATCAGGAGACACCCTGACAAGGGCAAT<br>GGACAGCAGCGGCCTACCAAACAGAAGTCTGAGGAGC<br>AGCTGAAGGATACCTACATGTGCAAGATCAACGAGAA<br>CATCGTGAACCAGGGCCAGATCAAGTTCGATTCTGAG<br>GTTATCAGCGCCTGGATGGCAGGCGACTCTAATATCCG<br>GAGCCTGGTTGAGAGCACTAGCCTGGACATCCTGAGC<br>ACATTCCTGATGGAAACACCTAAGCCTAGAAAGAAAG<br>GCAACAACAAAATCACAAATAAAAAGAGCGGCAAGA<br>AGAAGAAATGGATGGAAAAGAGAGCCGTGAAAAAAG<br>GATTCTACAAAAGATACCAACATCTGTTCGAGACAGA<br>TAGATGCAAACTGGCAAGCATCATTCTGGATGGCACC<br>GAGCGACTCCAGTGCCAGATTCCTCTGACAGAGATCCT<br>GGAAACATATAAGTCTAAGTGGGAGACTCTGACTCCA<br>TTCGAGGGCCTCGGCCAATTTAAGAGCCACGCCGTGG<br>CCGACAACACCGCCTTCGAGATTCTGCTGAGCGCCAA<br>GGAAATCATGAAGAACATCAAGGAGATGAACAAGAA<br>CAGCGCCCCAGGCCCTGATAAGGTGAGCCTGAGAGAT<br>CTGCTTCTGGCCGACCCCGAATGCAACGCCCTGGAAA<br>AGCTGTTCAACACCTGGCTGATCACCGGAATCATTCCA<br>AACAGCATAAAAGAATGTAGAAGCCTGCTGATCCCTA<br>AGACGGCGGACCCCGAGGCCCTGAAGGAACTGGGAA<br>ATTGGCGGCCTCTGACCATCGGCAGCATCGTGCTGCGG<br>CTGTTTAGCAGAATCATCACCAACAGACTGGCTAAGG<br>CCTGCCCCATCAACGCCCGGCAGAGAGGCTTCATCGC<br>CACCCCTGGTTGTAGCGAGAACCTGAAGATTCTTCACA<br>CAATCGTTAAACAAGCCAAGACCTCCAAAAAGAGCCT<br>GGGAGTGGTGTTCGTGGACATCGCCAAGGCCTTCGAC<br>TCAGTGAGCCACGACCACATTATGTGGGTGCTGCAGG<br>AGCGGGGACTCGACCAGCACATCGTGAACATTATCGA<br>GGACTCTTATAAGAAGATCCACACCAGAATGGAGGTG<br>GGCACCGAGAGAACCCCCCCTATCGAGATCAAGGTGG<br>GCGTGAAACAAGGGGACCCCATGAGCCCTCTGCTGTT<br>TAACCTGGCCATCGACCCTCTGATCACAGCCCTGGAGA<br>AAGCTAATACCGGCTTTAGCTACGGCAAGAATAAGAT	Fig. 3

	CACCTCTCTGGCCTTCGCGGATGACCTGGTGATGCTGT CCGACACCTGGGAGGGCATGAACAAGAATATCCAGAT CCTGGAAACATTTTGCAATCTGAGTGGCCTGAAGGTCC AGGCTAAGAAATGCTACGGCTTCTTCCTGAGCCCTACC CACGATTCATATACTATCAACAAATGCGACGCCTGGA AGATCGACAAGGACAGCCTGAACATGATCCAGCCTGG AGAATCTGAGAAGTACCTGGGCCTGAAGGTGGACCCT TGGATCGGCTTCAGCAAGCCCGTGCTGGCCGAAAAGC TTACAATCTGGCTGAAGCGGCTGACCGAAGCCCCTCTG AAGCCTAGCCAGAAACTGACAATGCTAAACATCTACA CCATCCCGAGAATCATATACCTGGCCGATCACACCGA CACCAAGAAAACCCTGCTGAGCAGCCTTGACGACAAC ATCAGGACGGTGGTGAAGGGCTGGCTGCACCTGCCTC CTGACACATGCAACGGCTTCATCTACACCAAAACTCG GGACGGCGGCCTGGGCGTGACCAGACTGGCTTCTCTT ATCCCCAGCATCCAGGCCAGACGGCTGCACCGGATCG CCACCAGCGAGGACGAGACAATCCGGAACATTGCTAT GGCCAACAATATCGAGGAAGAGTTCCAAAACCTGTGG GTGACCGCCGGCGGCAAGAAGGAAGAGATCCCCAGA ATCACCGACCCGGTGTCCATCGACTACAGACTGCCAA GACGGATTCTGGAACTGCTGAATGAGTGGGAGAAGCC AGCTCCAAAGAAGATGTACCCCATCCCTTGCAACTGG CGGGAAGCCGAGATGGCCCACTGGAAGAACCTGCCTT GCCAGGGATCAGGCATCGAGCACTTCGACAATGACAC CATCTCCAACGACTGGCTGCAGTTTCACCGGGGCTTCT CCGAGCGACAGTTCCTGATGGGCCTTAAGATCAGAGC CAACGTGTACCCTACCCGGGAGTACCAGGGCAGAGGC AGAACAAACAAGAACGTGAATTGTAGAAATTGCACCG CCTCTTACGAGAGCCTGTCTCATATCCTGGGCCAGTGC CCTGCCGTGCAGGGCGCTAGAATCCGGCGGCACAACA AGCTGTGCAGCATGCTGAAGCGGGAGGCCAAGGAACT GAAGTGGGTCGTGTACGAGGAACCTCACCTACATACA ACAGAAAAAGAGCTGAGAAAGCCTGACCTGATCTTCG TGAAGGAGGAAATGGCCCTGGTGGTCGATGTGACAGT GCGGTTTGAGTACAAGGAAAAGGTGTTCGAGGATGCC GCTGCTGAGAAAGTGCGGCACTACAAGGACCTGACCA GCCAGATCAAGGAGCTGACCGGCGCCAAAGAGATCGA GTACTTCGGCTTCCCCCTGGGCGCCAGAGGAAAGTGG CCTGAGATCAACGAGAAGGTGCTGACAGCCCTCGGCA TGCCTGATTACCAGCAGAAGCGCACCGCCAAACGGTT CAGCAAGAGAACCCTGCTGTACAGCATCGACGTGATC AACACCTTTGAGAACATCGGCAAGAACAACAAGAACA ACGTCCCCCATATGGGTGGAGGTAGCGGGGGCAGTGG AGGGATGGGGAGCGACTACAAAGACCATGACGGTGAT TATAAAGATCATGACATCGATTACAAGGATGACGATG ACAAGAAGTGATGACCTCGAGCTGGTACTGCATGCAC GCAATGCTAGCTGCCCCTTTCCCGTCCTGGGTACCCCG AGTCTCCCCCGACCTCGGGTCCCAGGTATGCTCCCACC TCCACCTGCCCCACTCACCACCTCTGCTAGTTCCAGAC ACCTCCCAAGCACGCAGCAATGCAGCTCAAAACGCTT AGCCTAGCCACACCCCCACGGGAAACAGCAGTGATTA ACCTTTAGCAATAAACGAAAGTTTAACTAAGCTATACT AACCCCAGGGTTGGTCAATTTCGTGCCAGCCACACCCT GGAGCTAGCAAAAAAAAAAAAAAAAAAAAAAAAAAAA AAAAAGTCTTCATCGGAAAGAACATGTGAGCAAAAGG CCAGCAAAAGGCCAGGAACCGTAAAAAGGCCGCGTTG	

	CTGGCGTTTTTCCATAGGCTCCGCCCCCCTGACGAGCA<br>TCACAAAAATCGACGCTCAAGTCAGAGGTGGCGAAAC<br>CCGACAGGACTATAAAGATACCAGGCGTTTCCCCCTG<br>GAAGCTCCCTCGTGCGCTCTCCTGTTCCGACCCTGCCG<br>CTTACCGGATACCTGTCCGCCTTTCTCCCTTCGGGAAG<br>CGTGGCGCTTTCTCATAGCTCACGCTGTAGGTATCTCA<br>GTTCGGTGTAGGTCGTTCGCTCCAAGCTGGGCTGTGTG<br>CACGAACCCCCCGTTCAGCCCGACCGCTGCGCCTTATC<br>CGGTAACTATCGTCTTGAGTCCAACCCGGTAAGACAC<br>GACTTATCGCCACTGGCAGCAGCCACTGGTAACAGGA<br>TTAGCAGAGCGAGGTATGTAGGCGGTGCTACAGAGTT<br>CTTGAAGTGGTGGCCTAACTACGGCTACACTAGAAGA<br>ACAGTATTTGGTATCTGCGCTCTGCTGAAGCCAGTTAC<br>CTTCGGAAAAAGAGTTGGTAGCTCTTGATCCGGCAAA<br>CAAACCACCGCTGGTAGCGGTGGTTTTTTTGTTTGCAA<br>GCAGCAGATTACGCGCAGAAAAAAAGGATCTCAAGAA<br>GATCCTTTGATCTTTTCTACGGGGTCTGACGCTCAGTG<br>GAACGAAAACTCACGTTAAGGGATTTTGGTCATGAGA<br>TTATCAAAAAGGATCTTCACCTAGATCCTTTTAAATTA<br>AAAATGAAGTTTTAAATCAATCTAAAGTATATATGAGT<br>AAACTTGGTCTGACAGTTACCAATGCTTAATCAGTGAG<br>GCACCTATCTCAGCGATCTGTCTATTTCGTTCATCCAT<br>AGTTGCCTGACTCCCCGTCGTGTAGATAACTACGATAC<br>GGGAGGGCTTACCATCTGGCCCCAGTGCTGCAATGAT<br>ACCGCGAGACCCACGCTCACCGGCTCCAGATTTATCA<br>GCAATAAACCAGCCAGCCGGAAGGGCCGAGCGCAGA<br>AGTGGTCCTGCAACTTTATCCGCCTCCATCCAGTCTAT<br>TAATTGTTGCCGGGAAGCTAGAGTAAGTAGTTCGCCA<br>GTTAATAGTTTGCGCAACGTTGTTGCCATTGCTACAGG<br>CATCGTGGTGTCACGCTCGTCGTTTGGTATGGCTTCAT<br>TCAGCTCCGGTTCCCAACGATCAAGGCGAGTTACATG<br>ATCCCCCATGTTGTGCAAAAAAGCGGTTAGCTCCTTCG<br>GTCCTCCGATCGTTGTCAGAAGTAAGTTGGCCGCAGTG<br>TTATCACTCATGGTTATGGCAGCACTGCATAATTCTCT<br>TACTGTCATGCCATCCGTAAGATGCTTTTCTGTGACTG<br>GTGAGTACTCAACCAAGTCATTCTGAGAATAGTGTATG<br>CGGCGACCGAGTTGCTCTTGCCCGGCGTCAATACGGG<br>ATAATACCGCGCCACATAGCAGAACTTTAAAAGTGCT<br>CATCATTGGAAAACGTTCTTCGGGGCGAAAACTCTCA<br>AGGATCTTACCGCTGTTGAGATCCAGTTCGATGTAACC<br>CACTCGTGCACCCAACTGATCTTCAGCATCTTTTACTTT<br>CACCAGCGTTTCTGGGTGAGCAAAAACAGGAAGGCAA<br>AATGCCGCAAAAAAGGGAATAAGGGCGACACGGAAA<br>TGTTGAATACTCATACTCTTCCTTTTTCAATATTATTGA<br>AGCATTTATCAGGGTTATTGTCTCATGAGCGGATACAT<br>ATTTGAATGTATTTAGAAAAATAAACAAAT	
PlaMe ZFTg	AGGGGTTCCGCGCACATTTCCCCGAAAAGTGCCACCT<br>GACGTCCCAATGATTAATACGACTCACTATAAGGAAT<br>AAACTAGTATTCTTCTGGTCCCCACAGACTCAGAGAGA<br>ACCCGCCACCATGCAGAAAGTGATGGTCACCGTTCCG<br>GACAAGAACCCTCCATGTCCATGCTGCGGCACCCGGG<br>TGAACAGCGTGCTGAACCTTATCGAGCACCTGAAGGT<br>CAGCCACGGAAAGCGGGGCGTGTGCTTCCGGTGCGCG<br>AAGTGTGGGAAGGAAAATAGCAACTACCACAGCGTGG<br>TGTGCCATTTCCCAAAATGTAGAGGACCTGAGACAGA<br>GAAAGCTCCAACCGGCGATTGGGCCTGCGAGACATGT	Fig. 3

	AACAAGCAGTTCAACACCAAGAGCGGCCTGTCCCAGC ACAAGAGAATCGCCCATCCCGCTATCCGGAACCAGGA GAGAATCGCCGCCAGCCAGCCTAAGCCTAACTCTCAA AGAGGAAAGCACAACAGCTGCTGGACGGTGGAAGAA GAACAGCTGCTGGCCGCTTTCAACAACATGTTCTGGGG CAAGAAAAATATCAATATCCTGATCTCTGATCACATCC ACATGAAAACAGCCAAGCAGATCAGCGAGAAGAGAC GGCTGCTGGGACTGAACAAGAACGCGACAGTGACAAC CACAAACCCCCTGCCTGTATCCAGCACCTGTCACCTGA AGATCCGGACCGACTCCCCTAATACCACCACCGGCCT GAAGGATACCTACATGTGCAAGATCAACGAGAACATC GTGAACCAGGGCCAGATCAAGTTCGATTCTGAGGTTA TCAGCGCCTGGATGGCAGGCGACTCTAATATCCGGAG CCTGGTTGAGAGCACTAGCCTGGACATCCTGAGCACA TTCCTGATGGAAACACCTAAGCCTAGAAAGAAAGGCA ACAACAAAATCACAAATAAAAAGAGCGGCAAGAAGA AGAAATGGATGGAAAAGAGAGCCGTGAAAAAAGGAT TCTACAAAAGATACCAACATCTGTTCGAGACAGATAG ATGCAAACTGGCAAGCATCATTCTGGATGGCACCGAG CGACTCCAGTGCCAGATTCCTCTGACAGAGATCCTGGA AACATATAAGTCTAAGTGGGAGACTCTGACTCCATTCG AGGGCCTCGGCCAATTTAAGAGCCACGCCGTGGCCGA CAACACCGCCTTCGAGATTCTGCTGAGCGCCAAGGAA ATCATGAAGAACATCAAGGAGATGAACAAGAACAGC GCCCCAGGCCCTGATAAGGTGAGCCTGAGAGATCTGC TTCTGGCCGACCCCGAATGCAACGCCCTGGAAAAGCT GTTCAACACCTGGCTGATCACCGGAATCATTCCAAACA GCATAAAAGAATGTAGAAGCCTGCTGATCCCTAAGAC GGCGGACCCCGAGGCCCTGAAGGAACTGGGAAATTGG CGGCCTCTGACCATCGGCAGCATCGTGCTGCGGCTGTT TAGCAGAATCATCACCAACAGACTGGCTAAGGCCTGC CCCATCAACGCCCGGCAGAGAGGCTTCATCGCCACCC CTGGTTGTAGCGAGAACCTGAAGATTCTTCACACAATC GTTAAACAAGCCAAGACCTCCAAAAAGAGCCTGGGAG TGGTGTTCGTGGACATCGCCAAGGCCTTCGACTCAGTG AGCCACGACCACATTATGTGGGTGCTGCAGGAGCGGG GACTCGACCAGCACATCGTGAACATTATCGAGGACTC TTATAAGAAGATCCACACCAGAATGGAGGTGGGCACC GAGAGAACCCCCCCTATCGAGATCAAGGTGGGCGTGA AACAAGGGGACCCCATGAGCCCTCTGCTGTTTAACCTG GCCATCGACCCTCTGATCACAGCCCTGGAGAAAGCTA ATACCGGCTTTAGCTACGGCAAGAATAAGATCACCTCT CTGGCCTTCGCGGATGACCTGGTGATGCTGTCCGACAC CTGGGAGGGCATGAACAAGAATATCCAGATCCTGGAA ACATTTTGCAATCTGAGTGGCCTGAAGGTCCAGGCTAA GAAATGCTACGGCTTCTTCCTGAGCCCTACCCACGATT CATATACTATCAACAAATGCGACGCCTGGAAGATCGA CAAGGACAGCCTGAACATGATCCAGCCTGGAGAATCT GAGAAGTACCTGGGCCTGAAGGTGGACCCTTGGATCG GCTTCAGCAAGCCCGTGCTGGCCGAAAAGCTTACAAT CTGGCTGAAGCGGCTGACCGAAGCCCCTCTGAAGCCT AGCCAGAAACTGACAATGCTAAACATCTACACCATCC CGAGAATCATATACCTGGCCGATCACACCGACACCAA GAAAACCCTGCTGAGCAGCCTTGACGACAACATCAGG ACGGTGGTGAAGGGCTGGCTGCACCTGCCTCCTGACA CATGCAACGGCTTCATCTACACCAAAACTCGGGACGG	

	CGGCCTGGGCGTGACCAGACTGGCTTCTCTTATCCCCA<br>GCATCCAGGCCAGACGGCTGCACCGGATCGCCACCAG<br>CGAGGACGAGACAATCCGGAACATTGCTATGGCCAAC<br>AATATCGAGGAAGAGTTCCAAAACCTGTGGGTGACCG<br>CCGGCGGCAAGAAGGAAGAGATCCCCAGAATCACCGA<br>CCCGGTGTCCATCGACTACAGACTGCCAAGACGGATT<br>CTGGAACTGCTGAATGAGTGGGAGAAGCCAGCTCCAA<br>AGAAGATGTACCCCATCCCTTGCAACTGGCGGGAAGC<br>CGAGATGGCCCACTGGAAGAACCTGCCTTGCCAGGGA<br>TCAGGCATCGAGCACTTCGACAATGACACCATCTCCA<br>ACGACTGGCTGCAGTTTCACCGGGGCTTCTCCGAGCGA<br>CAGTTCCTGATGGGCCTTAAGATCAGAGCCAACGTGT<br>ACCCTACCCGGGAGTACCAGGGCAGAGGCAGAACAAA<br>CAAGAACGTGAATTGTAGAAATTGCACCGCCTCTTAC<br>GAGAGCCTGTCTCATATCCTGGGCCAGTGCCCTGCCGT<br>GCAGGGCGCTAGAATCCGGCGGCACAACAAGCTGTGC<br>AGCATGCTGAAGCGGGAGGCCAAGGAACTGAAGTGG<br>GTCGTGTACGAGGAACCTCACCTACATACAACAGAAA<br>AAGAGCTGAGAAAGCCTGACCTGATCTTCGTGAAGGA<br>GGAAATGGCCCTGGTGGTCGATGTGACAGTGCGGTTT<br>GAGTACAAGGAAAAGGTGTTCGAGGATGCCGCTGCTG<br>AGAAAGTGCGGCACTACAAGGACCTGACCAGCCAGAT<br>CAAGGAGCTGACCGGCGCCAAAGAGATCGAGTACTTC<br>GGCTTCCCCCTGGGCGCCAGAGGAAAGTGGCCTGAGA<br>TCAACGAGAAGGTGCTGACAGCCCTCGGCATGCCTGA<br>TTACCAGCAGAAGCGCACCGCCAAACGGTTCAGCAAG<br>AGAACCCTGCTGTACAGCATCGACGTGATCAACACCTT<br>TGAGAACATCGGCAAGAACAACAAGAACAACGTCCCC<br>CATATGGGTGGAGGTAGCGGGGGCAGTGGAGGGATGG<br>GGAGCGACTACAAAGACCATGACGGTGATTATAAAGA<br>TCATGACATCGATTACAAGGATGACGATGACAAGAAG<br>TGATGACCTCGAGCTGGTACTGCATGCACGCAATGCTA<br>GCTGCCCCTTTCCCGTCCTGGGTACCCCGAGTCTCCCC<br>CGACCTCGGGTCCCAGGTATGCTCCCACCTCCACCTGC<br>CCCACTCACCACCTCTGCTAGTTCCAGACACCTCCCAA<br>GCACGCAGCAATGCAGCTCAAAACGCTTAGCCTAGCC<br>ACACCCCCACGGGAAACAGCAGTGATTAACCTTTAGC<br>AATAAACGAAAGTTTAACTAAGCTATACTAACCCCAG<br>GGTTGGTCAATTTCGTGCCAGCCACACCCTGGAGCTAG<br>CAAAAAAAAAAAAAAAAAAAAAAAAAAAAAAAAAAGTC<br>TTCATCGGAAAGAACATGTGAGCAAAAGGCCAGCAAA<br>AGGCCAGGAACCGTAAAAAGGCCGCGTTGCTGGCGTT<br>TTTCCATAGGCTCCGCCCCCCTGACGAGCATCACAAAA<br>ATCGACGCTCAAGTCAGAGGTGGCGAAACCCGACAGG<br>ACTATAAAGATACCAGGCGTTTCCCCCTGGAAGCTCCC<br>TCGTGCGCTCTCCTGTTCCGACCCTGCCGCTTACCGGA<br>TACCTGTCCGCCTTTCTCCCTTCGGGAAGCGTGGCGCT<br>TTCTCATAGCTCACGCTGTAGGTATCTCAGTTCGGTGT<br>AGGTCGTTCGCTCCAAGCTGGGCTGTGTGCACGAACCC<br>CCCGTTCAGCCCGACCGCTGCGCCTTATCCGGTAACTA<br>TCGTCTTGAGTCCAACCCGGTAAGACACGACTTATCGC<br>CACTGGCAGCAGCCACTGGTAACAGGATTAGCAGAGC<br>GAGGTATGTAGGCGGTGCTACAGAGTTCTTGAAGTGG<br>TGGCCTAACTACGGCTACACTAGAAGAACAGTATTTG<br>GTATCTGCGCTCTGCTGAAGCCAGTTACCTTCGGAAAA<br>AGAGTTGGTAGCTCTTGATCCGGCAAACAAACCACCG	

	CTGGTAGCGGTGGTTTTTTTGTTTGCAAGCAGCAGATT ACGCGCAGAAAAAAAGGATCTCAAGAAGATCCTTTGA TCTTTTCTACGGGGTCTGACGCTCAGTGGAACGAAAAC TCACGTTAAGGGATTTTGGTCATGAGATTATCAAAAAG GATCTTCACCTAGATCCTTTTAAATTAAAAATGAAGTT TTAAATCAATCTAAAGTATATATGAGTAAACTTGGTCT GACAGTTACCAATGCTTAATCAGTGAGGCACCTATCTC AGCGATCTGTCTATTTCGTTCATCCATAGTTGCCTGAC TCCCCGTCGTGTAGATAACTACGATACGGGAGGGCTT ACCATCTGGCCCCAGTGCTGCAATGATACCGCGAGAC CCACGCTCACCGGCTCCAGATTTATCAGCAATAAACCA GCCAGCCGGAAGGGCCGAGCGCAGAAGTGGTCCTGCA ACTTTATCCGCCTCCATCCAGTCTATTAATTGTTGCCG GGAAGCTAGAGTAAGTAGTTCGCCAGTTAATAGTTTG CGCAACGTTGTTGCCATTGCTACAGGCATCGTGGTGTC ACGCTCGTCGTTTGGTATGGCTTCATTCAGCTCCGGTT CCCAACGATCAAGGCGAGTTACATGATCCCCCATGTTG TGCAAAAAAGCGGTTAGCTCCTTCGGTCCTCCGATCGT TGTCAGAAGTAAGTTGGCCGCAGTGTTATCACTCATGG TTATGGCAGCACTGCATAATTCTCTTACTGTCATGCCA TCCGTAAGATGCTTTTCTGTGACTGGTGAGTACTCAAC CAAGTCATTCTGAGAATAGTGTATGCGGCGACCGAGT TGCTCTTGCCCGGCGTCAATACGGGATAATACCGCGCC ACATAGCAGAACTTTAAAAGTGCTCATCATTGGAAAA CGTTCTTCGGGGCGAAAACTCTCAAGGATCTTACCGCT GTTGAGATCCAGTTCGATGTAACCCACTCGTGCACCCA ACTGATCTTCAGCATCTTTTACTTTCACCAGCGTTTCTG GGTGAGCAAAAACAGGAAGGCAAAATGCCGCAAAAA AGGGAATAAGGGCGACACGGAAATGTTGAATACTCAT ACTCTTCCTTTTTCAATATTATTGAAGCATTTATCAGGG TTATTGTCTCATGAGCGGATACATATTTGAATGTATTT AGAAAAATAAACAAAT	
PlaMe spacTg	AGGGGTTCCGCGCACATTTCCCCGAAAAGTGCCACCT GACGTCCCAATGATTAATACGACTCACTATAAGGAAT AAACTAGTATTCTTCTGGTCCCCACAGACTCAGAGAGA ACCCGCCACCATGCAGAAAACCATCATCCAGCTGCCG AATGATAACCCTGCCTGTCCTTTCTGCGGCGATCACGT GGGCAAGCCTTCCGCTCTGAACGTGCACCTGAAGCGC AACCACGGAGGCCGTGAGGTGGAATTCCAGTGTTCTA TGTGCAACAAGGCCGACCCCAAGGCCCACAGCATCCT GTGCCACATCCCTAAGTGTAAAGGAAAGGTGACCGAG GAACCCACCGGCGATTGGGCCTGCGAGACATGTAACA AGCAGTTCAACACCAAGAGCGGCCTGTCCCAGCACAA GAGAATCGCCCATCCCGCTATCCGGAACCAGGAGAGA ATCGCCGCCAGCCAGCCTAAGCCTAACTCTCAAAGAG GAAAGCACAACAGCTGCTGGACGGTGGAAGAAGAAC AGCTGCTGGCCGCTTTCAACAACATGTTCTGGGGCAAG AAAAATATCAATATCCTGATCTCTGATCACATCCACAT GAAAACAGCCAAGCAGATCAGCGAGAAGAGACGGCT GAAGCCTGCCGAGGAGCCTAGAGAGGAACCTGGCACA TGCCACCACACCAGACGGGCAGCTGCCAGCCTGAGAA CAGAGCCCGAGATGTCCCACCACGCTCAGGCCGAAGA TCGGGATAATGGCCCTGGCCGGAGACCCCTGCCTGGT AGAGCCGCCGCCGGCGGCAGAACAATGGACGAGATCA GGAGACACCCTGACAAGGGCAATGGACAGCAGCGGCC TACCAAACAGAAGTCTGAGGAGCAGCTGAAGGATACC	Fig. 3

	TACATGTGCAAGATCAACGAGAACATCGTGAACCAGG<br>GCCAGATCAAGTTCGATTCTGAGGTTATCAGCGCCTGG<br>ATGGCAGGCGACTCTAATATCCGGAGCCTGGTTGAGA<br>GCACTAGCCTGGACATCCTGAGCACATTCCTGATGGA<br>AACACCTAAGCCTAGAAAGAAAGGCAACAACAAAATC<br>ACAAATAAAAAGAGCGGCAAGAAGAAGAAATGGATG<br>GAAAAGAGAGCCGTGAAAAAAGGATTCTACAAAAGA<br>TACCAACATCTGTTCGAGACAGATAGATGCAAACTGG<br>CAAGCATCATTCTGGATGGCACCGAGCGACTCCAGTG<br>CCAGATTCCTCTGACAGAGATCCTGGAAACATATAAG<br>TCTAAGTGGGAGACTCTGACTCCATTCGAGGGCCTCGG<br>CCAATTTAAGAGCCACGCCGTGGCCGACAACACCGCC<br>TTCGAGATTCTGCTGAGCGCCAAGGAAATCATGAAGA<br>ACATCAAGGAGATGAACAAGAACAGCGCCCCAGGCCC<br>TGATAAGGTGAGCCTGAGAGATCTGCTTCTGGCCGAC<br>CCCGAATGCAACGCCCTGGAAAAGCTGTTCAACACCT<br>GGCTGATCACCGGAATCATTCCAAACAGCATAAAAGA<br>ATGTAGAAGCCTGCTGATCCCTAAGACGGCGGACCCC<br>GAGGCCCTGAAGGAACTGGGAAATTGGCGGCCTCTGA<br>CCATCGGCAGCATCGTGCTGCGGCTGTTTAGCAGAATC<br>ATCACCAACAGACTGGCTAAGGCCTGCCCCATCAACG<br>CCCGGCAGAGAGGCTTCATCGCCACCCCTGGTTGTAGC<br>GAGAACCTGAAGATTCTTCACACAATCGTTAAACAAG<br>CCAAGACCTCCAAAAAGAGCCTGGGAGTGGTGTTCGT<br>GGACATCGCCAAGGCCTTCGACTCAGTGAGCCACGAC<br>CACATTATGTGGGTGCTGCAGGAGCGGGGACTCGACC<br>AGCACATCGTGAACATTATCGAGGACTCTTATAAGAA<br>GATCCACACCAGAATGGAGGTGGGCACCGAGAGAACC<br>CCCCCTATCGAGATCAAGGTGGGCGTGAAACAAGGGG<br>ACCCCATGAGCCCTCTGCTGTTTAACCTGGCCATCGAC<br>CCTCTGATCACAGCCCTGGAGAAAGCTAATACCGGCTT<br>TAGCTACGGCAAGAATAAGATCACCTCTCTGGCCTTCG<br>CGGATGACCTGGTGATGCTGTCCGACACCTGGGAGGG<br>CATGAACAAGAATATCCAGATCCTGGAAACATTTTGC<br>AATCTGAGTGGCCTGAAGGTCCAGGCTAAGAAATGCT<br>ACGGCTTCTTCCTGAGCCCTACCCACGATTCATATACT<br>ATCAACAAATGCGACGCCTGGAAGATCGACAAGGACA<br>GCCTGAACATGATCCAGCCTGGAGAATCTGAGAAGTA<br>CCTGGGCCTGAAGGTGGACCCTTGGATCGGCTTCAGC<br>AAGCCCGTGCTGGCCGAAAAGCTTACAATCTGGCTGA<br>AGCGGCTGACCGAAGCCCCTCTGAAGCCTAGCCAGAA<br>ACTGACAATGCTAAACATCTACACCATCCCGAGAATC<br>ATATACCTGGCCGATCACACCGACACCAAGAAAACCC<br>TGCTGAGCAGCCTTGACGACAACATCAGGACGGTGGT<br>GAAGGGCTGGCTGCACCTGCCTCCTGACACATGCAAC<br>GGCTTCATCTACACCAAAACTCGGGACGGCGGCCTGG<br>GCGTGACCAGACTGGCTTCTCTTATCCCCAGCATCCAG<br>GCCAGACGGCTGCACCGGATCGCCACCAGCGAGGACG<br>AGACAATCCGGAACATTGCTATGGCCAACAATATCGA<br>GGAAGAGTTCCAAAACCTGTGGGTGACCGCCGGCGGC<br>AAGAAGGAAGAGATCCCCAGAATCACCGACCCGGTGT<br>CCATCGACTACAGACTGCCAAGACGGATTCTGGAACT<br>GCTGAATGAGTGGGAGAAGCCAGCTCCAAAGAAGATG<br>TACCCCATCCCTTGCAACTGGCGGGAAGCCGAGATGG<br>CCCACTGGAAGAACCTGCCTTGCCAGGGATCAGGCAT<br>CGAGCACTTCGACAATGACACCATCTCCAACGACTGG	

	CTGCAGTTTCACCGGGGCTTCTCCGAGCGACAGTTCCT GATGGGCCTTAAGATCAGAGCCAACGTGTACCCTACC CGGGAGTACCAGGGCAGAGGCAGAACAAACAAGAAC GTGAATTGTAGAAATTGCACCGCCTCTTACGAGAGCCT GTCTCATATCCTGGGCCAGTGCCCTGCCGTGCAGGGCG CTAGAATCCGGCGGCACAACAAGCTGTGCAGCATGCT GAAGCGGGAGGCCAAGGAACTGAAGTGGGTCGTGTAC GAGGAACCTCACCTACATACAACAGAAAAAGAGCTGA GAAAGCCTGACCTGATCTTCGTGAAGGAGGAAATGGC CCTGGTGGTCGATGTGACAGTGCGGTTTGAGTACAAG GAAAAGGTGTTCGAGGATGCCGCTGCTGAGAAAGTGC GGCACTACAAGGACCTGACCAGCCAGATCAAGGAGCT GACCGGCGCCAAAGAGATCGAGTACTTCGGCTTCCCC CTGGGCGCCAGAGGAAAGTGGCCTGAGATCAACGAGA AGGTGCTGACAGCCCTCGGCATGCCTGATTACCAGCA GAAGCGCACCGCCAAACGGTTCAGCAAGAGAACCCTG CTGTACAGCATCGACGTGATCAACACCTTTGAGAACAT CGGCAAGAACAACAAGAACAACGTCCCCCATATGGGT GGAGGTAGCGGGGGCAGTGGAGGGATGGGGAGCGAC TACAAAGACCATGACGGTGATTATAAAGATCATGACA TCGATTACAAGGATGACGATGACAAGAAGTGATGACC TCGAGCTGGTACTGCATGCACGCAATGCTAGCTGCCCC TTTCCCGTCCTGGGTACCCCGAGTCTCCCCCGACCTCG GGTCCCAGGTATGCTCCCACCTCCACCTGCCCCACTCA CCACCTCTGCTAGTTCCAGACACCTCCCAAGCACGCAG CAATGCAGCTCAAAACGCTTAGCCTAGCCACACCCCC ACGGGAAACAGCAGTGATTAACCTTTAGCAATAAACG AAAGTTTAACTAAGCTATACTAACCCCAGGGTTGGTCA ATTTCGTGCCAGCCACACCCTGGAGCTAGCAAAAAAA AAAAAAAAAAAAAAAAAAAAAAAAAGTCTTCATCGG AAAGAACATGTGAGCAAAAGGCCAGCAAAAGGCCAG GAACCGTAAAAAGGCCGCGTTGCTGGCGTTTTTCCATA GGCTCCGCCCCCCTGACGAGCATCACAAAAATCGACG CTCAAGTCAGAGGTGGCGAAACCCGACAGGACTATAA AGATACCAGGCGTTTCCCCCTGGAAGCTCCCTCGTGCG CTCTCCTGTTCCGACCCTGCCGCTTACCGGATACCTGT CCGCCTTTCTCCCTTCGGGAAGCGTGGCGCTTTCTCAT AGCTCACGCTGTAGGTATCTCAGTTCGGTGTAGGTCGT TCGCTCCAAGCTGGGCTGTGTGCACGAACCCCCCGTTC AGCCCGACCGCTGCGCCTTATCCGGTAACTATCGTCTT GAGTCCAACCCGGTAAGACACGACTTATCGCCACTGG CAGCAGCCACTGGTAACAGGATTAGCAGAGCGAGGTA TGTAGGCGGTGCTACAGAGTTCTTGAAGTGGTGGCCTA ACTACGGCTACACTAGAAGAACAGTATTTGGTATCTGC GCTCTGCTGAAGCCAGTTACCTTCGGAAAAAGAGTTG GTAGCTCTTGATCCGGCAAACAAACCACCGCTGGTAG CGGTGGTTTTTTTGTTTGCAAGCAGCAGATTACGCGCA GAAAAAAAGGATCTCAAGAAGATCCTTTGATCTTTTCT ACGGGGTCTGACGCTCAGTGGAACGAAAACTCACGTT AAGGGATTTTGGTCATGAGATTATCAAAAAGGATCTTC ACCTAGATCCTTTTAAATTAAAAATGAAGTTTTAAATC AATCTAAAGTATATATGAGTAAACTTGGTCTGACAGTT ACCAATGCTTAATCAGTGAGGCACCTATCTCAGCGATC TGTCTATTTCGTTCATCCATAGTTGCCTGACTCCCCGTC GTGTAGATAACTACGATACGGGAGGGCTTACCATCTG GCCCCAGTGCTGCAATGATACCGCGAGACCCACGCTC	

	ACCGGCTCCAGATTTATCAGCAATAAACCAGCCAGCC GGAAGGGCCGAGCGCAGAAGTGGTCCTGCAACTTTAT CCGCCTCCATCCAGTCTATTAATTGTTGCCGGGAAGCT AGAGTAAGTAGTTCGCCAGTTAATAGTTTGCGCAACGT TGTTGCCATTGCTACAGGCATCGTGGTGTCACGCTCGT CGTTTGGTATGGCTTCATTCAGCTCCGGTTCCCAACGA TCAAGGCGAGTTACATGATCCCCCATGTTGTGCAAAA AAGCGGTTAGCTCCTTCGGTCCTCCGATCGTTGTCAGA AGTAAGTTGGCCGCAGTGTTATCACTCATGGTTATGGC AGCACTGCATAATTCTCTTACTGTCATGCCATCCGTAA GATGCTTTTCTGTGACTGGTGAGTACTCAACCAAGTCA TTCTGAGAATAGTGTATGCGGCGACCGAGTTGCTCTTG CCCGGCGTCAATACGGGATAATACCGCGCCACATAGC AGAACTTTAAAAGTGCTCATCATTGGAAAACGTTCTTC GGGGCGAAAACTCTCAAGGATCTTACCGCTGTTGAGA TCCAGTTCGATGTAACCCACTCGTGCACCCAACTGATC TTCAGCATCTTTTACTTTCACCAGCGTTTCTGGGTGAG CAAAAACAGGAAGGCAAAATGCCGCAAAAAAGGGAA TAAGGGCGACACGGAAATGTTGAATACTCATACTCTTC CTTTTTCAATATTATTGAAGCATTTATCAGGGTTATTGT CTCATGAGCGGATACATATTTGAATGTATTTAGAAAAA TAAACAAAT	
TaGu NPm	AGGGGTTCCGCGCACATTTCCCCGAAAAGTGCCACCT GACGTCCCAATGATTAATACGACTCACTATAAGGAAT AAACTAGTATTCTTCTGGTCCCCACAGACTCAGAGAGA ACCCGCCACCATGCAGAAAACCATCATCCAGCTGCCG AATGATAACCCTGCCTGTCCTTTCTGCGGCGATCACGT GGGCAAGCCTTCCGCTCTGAACGTGCACCTGAAGCGC AACCACGGAGGCCGTGAGGTGGAATTCCAGTGTTCTA TGTGCAACAAGGCCGACCCCAAGGCCCACAGCATCCT GTGCCACATCCCTAAGTGTAAAGGAAAGGTGACCGAG GAACCCACCGGCGATTGGGCCTGCGAGACATGTAACA AGCAGTTCAACACCAAGAGCGGCCTGTCCCAGCACAA GAGAATCGCCCATCCCGCTATCCGGAACCAGGAGAGA ATCGCCGCCAGCCAGCCTAAGCCTAACTCTCAAAGAG GAAAGCACAACAGCTGCTGGACGGTGGAAGAAGAAC AGCTGCTGGCCGCTTTCAACAACATGTTCTGGGGCAAG AAAAATATCAATATCCTGATCTCTGATCACATCCACAT GAAAACAGCCAAGCAGATCAGCGAGAAGAGACGGCT GCTGGGACTGAACAAGAACGCGACAGTGACAACCACA AACCCCCTGCCTGTATCCAGCACCTGTCACCTGAAGAT CCGGACCGACTCCCCTAATACCACCACCGGCCTGCAA GCCTACTACAAAAAGACACTGGAGGAGCGGCTGAGCG CCGGAGCCCTGAATACCTTCCCCCGCGCCTTCAAGCAA GTGATGGAAGGCAGAGATATCAAGCTGGTGATCAACC AGACAGCCCAGGACTGCTTCGGCTGTCTGGAGAGCAT CTCTCAGATCAGAACAGCGACCAGAGATAAGAAAGAT ACCGTGACCAGAGAGAAGCACCCGAAGAAGCCATTCC AAAAGTGGATGAAGGACCGGGCCATAAAGAAGGGCA ACTACCTGAGGTTCCAGAGATTGTTCTACCTGGACCGA GGCAAACTGGCCAAGATCATCCTGGACGATATCGAAT GCCTGAGCTGCGACATCCCCCTGTCTGAGATCTACAGC GTGTTTAAGACAAGATGGGAAACCACAGGAAGCTTCA AATCTCTGGGCGATTTTAAAACCTACGGCAAGGCCGA CAACACTGCCTTCAGAGAACTGATTACAGCTAAGGAA ATCGAGAAAAACGTGCAGGAGATGAGCAAGGGCAGC	Fig. 3

	GCCCCTGGCCCTGACGGTATCACCCTGGGAGATGTGGT GAAAATGGACCCCGAGTTCAGCCGCACAATGGAGATC TTCAACCTGTGGCTGACCACTGGCAAAATCCCTGACAT GGTGCGGGGATGCCGCACCGTGCTGATACCTAAGAGC AGCAAGCCAGATAGACTGAAGGATATCAACAACTGGC GGCCTATCACAATCGGCAGCATCCTGCTCCGGCTGTTT AGCAGAATCGTAACCGCCCGTCTGAGTAAGGCCTGTC CTCTGAACCCCAGACAGCGGGGCTTCATCCGGGCCGC CGGCTGCAGCGAGAACCTCAAACTGCTGCAGACCATC ATCTGGTCGGCCAAACGAGAGCACAGACCTCTCGGCG TGGTGTTCGTGGACATCGCCAAAGCTTTTGATACCGTC TCCCACCAGCACATCATCCACGCCCTGCAGCAGCGGG AAGTGGACCCTCACATCGTGGGACTGGTGTCCAACAT GTACGAGAACATCAGCACTTATATCACCACCAAGAGA AACACCCACACAGACAAGATCCAGATCAGAGTGGGCG TTAAGCAGGGCGATCCTATGTCTCCTCTGCTGTTCAAC CTGGCTATGGACCCTCTGCTCTGTAAACTGGAGGAGTC TGGCAAGGGATACCACAGAGGCCAAAGCTCCATCACC GCTATGGCCTTTGCCGACGACCTGGTGCTGCTGTCTGA TTCTTGGGAGAACATGAATACCAACATAAGCATCCTG GAAACATTCTGCAACCTTACAGGCCTGAAAACCCAGG GACAAAAGTGCCACGGCTTTTACATCAAGCCTACCAA AGACAGCTACACCATCAACGACTGCGCCGCCTGGACC ATCAACGGCACCCCTTTAAACATGATCGACCCTGGAG AAAGCGAGAAGTATCTGGGCCTGCAGTTCGACCCCTG GATCGGCATCGCTAGAAGCGGCCTGAGCACAAAGCTG GACTTTTGGCTGCAGAGAATTGACCAGGCCCCTCTGAA GCCTCTGCAGAAAACCGACATCCTGAAAACCTACACA ATCCCTCGGCTGATCTACATTGCCGATCACAGCGAGGT AAAGACCGCCCTGCTGGAAACCCTGGACCAAAAGATC AGAACCGCCGTGAAGGAATGGCTCCACCTGCCCCCCT GCACCTGCGACGCTATCCTGTACAGCAGCACCAGGGA CGGCGGCCTGGGCATCACCAAGCTGGCGGGCCTGATC CCCTCCGTCCAGGCTAGACGGCTGCATAGAATCGCCC AGAGCAGCGATGACACAATGAAGTGCTTTATGGAAAA GGAAAAGATGGAACAGCTGCACAAGAAGCTGTGGATT CAGGCCGGTGGCGACAGAGAGAACATTCCTAGCATCT GGGAGGCGCCGCCTAGTAGCGAGCCTCCCAACAACGT GTCTACCAATTCTGAGTGGGAAGCCCCTACACAGAAG GACAAGTTCCCTAAGCCTTGTAATTGGAGAAAGAACG AGTTCAAGAAGTGGACAAAGCTGGCCTCTCAGGGCCG GGGAATTGTGAATTTCGAGCGGGACAAGATCAGCAAT CACTGGATTCAGTACTACAGAAGAATCCCACACAGAA AGCTGCTGACGGCCCTTCAGCTGAGAGCCAACGTGTA CCCCACGCGGGAGTTCCTGGCCAGAGGTAGACAGGAC CAGTACATCAAGGCCTGCAGACATTGTGATGCTGATAT CGAGTCTTGCGCCCACATCATCGGCAACTGCCCTGTGA CACAGGACGCGAGAATCAAAAGACACAACTACATCTG CGAGCTGCTGCTGGAAGAGGCCAAGAAGAAGGACTGG GTGGTGTTCAAGGAACCCCACATCAGAGACAGCAATA AGGAACTCTATAAACCTGACCTGATCTTCGTGAAGGA CGCCCGGGCCCTGGTCGTGGACGTGACCGTGAGATAC GAGGCCGCCAAGTCTAGCCTGGAGGAGGCTGCCGCCG AGAAAGTGCGGAAGTACAAGCACCTTGAAACAGAAGT GCGGCACCTGACCAACGCCAAGGACGTCACATTCGTG GGCTTCCCCCTGGGCGCCAGAGGCAAATGGCACCAGG	

	ATAACTTCAAGCTGCTGACAGAGCTGGGCCTGAGCAA<br>GTCCCGCCAAGTGAAGATGGCCGAGACCTTCAGCACC<br>GTGGCCCTGTTCTCCTCTGTGGACATCGTGCACATGTT<br>CGCTAGCAGAGCCAGAAAGAGCATGGTGATGCATATG<br>GGTGGAGGTAGCGGGGGCAGTGGAGGGATGGGGAGC<br>GACTACAAAGACCATGACGGTGATTATAAAGATCATG<br>ACATCGATTACAAGGATGACGATGACAAGAAGTGATG<br>ACCTCGAGCTGGTACTGCATGCACGCAATGCTAGCTGC<br>CCCTTTCCCGTCCTGGGTACCCCGAGTCTCCCCCGACC<br>TCGGGTCCCAGGTATGCTCCCACCTCCACCTGCCCCAC<br>TCACCACCTCTGCTAGTTCCAGACACCTCCCAAGCACG<br>CAGCAATGCAGCTCAAAACGCTTAGCCTAGCCACACC<br>CCCACGGGAAACAGCAGTGATTAACCTTTAGCAATAA<br>ACGAAAGTTTAACTAAGCTATACTAACCCCAGGGTTG<br>GTCAATTTCGTGCCAGCCACACCCTGGAGCTAGCAAA<br>AAAAAAAAAAAAAAAAAAAAAAAAAAAAAGTCTTCA<br>TCGGAAAGAACATGTGAGCAAAAGGCCAGCAAAAGG<br>CCAGGAACCGTAAAAAGGCCGCGTTGCTGGCGTTTTTC<br>CATAGGCTCCGCCCCCCTGACGAGCATCACAAAAATC<br>GACGCTCAAGTCAGAGGTGGCGAAACCCGACAGGACT<br>ATAAAGATACCAGGCGTTTCCCCCTGGAAGCTCCCTCG<br>TGCGCTCTCCTGTTCCGACCCTGCCGCTTACCGGATAC<br>CTGTCCGCCTTTCTCCCTTCGGGAAGCGTGGCGCTTTC<br>TCATAGCTCACGCTGTAGGTATCTCAGTTCGGTGTAGG<br>TCGTTCGCTCCAAGCTGGGCTGTGTGCACGAACCCCCC<br>GTTCAGCCCGACCGCTGCGCCTTATCCGGTAACTATCG<br>TCTTGAGTCCAACCCGGTAAGACACGACTTATCGCCAC<br>TGGCAGCAGCCACTGGTAACAGGATTAGCAGAGCGAG<br>GTATGTAGGCGGTGCTACAGAGTTCTTGAAGTGGTGG<br>CCTAACTACGGCTACACTAGAAGAACAGTATTTGGTAT<br>CTGCGCTCTGCTGAAGCCAGTTACCTTCGGAAAAAGA<br>GTTGGTAGCTCTTGATCCGGCAAACAAACCACCGCTG<br>GTAGCGGTGGTTTTTTTGTTTGCAAGCAGCAGATTACG<br>CGCAGAAAAAAAGGATCTCAAGAAGATCCTTTGATCT<br>TTTCTACGGGGTCTGACGCTCAGTGGAACGAAAACTC<br>ACGTTAAGGGATTTTGGTCATGAGATTATCAAAAAGG<br>ATCTTCACCTAGATCCTTTTAAATTAAAAATGAAGTTT<br>TAAATCAATCTAAAGTATATATGAGTAAACTTGGTCTG<br>ACAGTTACCAATGCTTAATCAGTGAGGCACCTATCTCA<br>GCGATCTGTCTATTTCGTTCATCCATAGTTGCCTGACTC<br>CCCGTCGTGTAGATAACTACGATACGGGAGGGCTTAC<br>CATCTGGCCCCAGTGCTGCAATGATACCGCGAGACCC<br>ACGCTCACCGGCTCCAGATTTATCAGCAATAAACCAG<br>CCAGCCGGAAGGGCCGAGCGCAGAAGTGGTCCTGCAA<br>CTTTATCCGCCTCCATCCAGTCTATTAATTGTTGCCGG<br>GAAGCTAGAGTAAGTAGTTCGCCAGTTAATAGTTTGC<br>GCAACGTTGTTGCCATTGCTACAGGCATCGTGGTGTCA<br>CGCTCGTCGTTTGGTATGGCTTCATTCAGCTCCGGTTC<br>CCAACGATCAAGGCGAGTTACATGATCCCCCATGTTGT<br>GCAAAAAAGCGGTTAGCTCCTTCGGTCCTCCGATCGTT<br>GTCAGAAGTAAGTTGGCCGCAGTGTTATCACTCATGGT<br>TATGGCAGCACTGCATAATTCTCTTACTGTCATGCCAT<br>CCGTAAGATGCTTTTCTGTGACTGGTGAGTACTCAACC<br>AAGTCATTCTGAGAATAGTGTATGCGGCGACCGAGTT<br>GCTCTTGCCCGGCGTCAATACGGGATAATACCGCGCC<br>ACATAGCAGAACTTTAAAAGTGCTCATCATTGGAAAA	

	CGTTCTTCGGGGCGAAAACTCTCAAGGATCTTACCGCT GTTGAGATCCAGTTCGATGTAACCCACTCGTGCACCCA ACTGATCTTCAGCATCTTTTACTTTCACCAGCGTTTCTG GGTGAGCAAAAACAGGAAGGCAAAATGCCGCAAAAA AGGGAATAAGGGCGACACGGAAATGTTGAATACTCAT ACTCTTCCTTTTTCAATATTATTGAAGCATTTATCAGGG TTATTGTCTCATGAGCGGATACATATTTGAATGTATTT AGAAAAATAAACAAAT	
TaGu ZFPm	AGGGGTTCCGCGCACATTTCCCCGAAAAGTGCCACCT GACGTCCCAATGATTAATACGACTCACTATAAGGAAT AAACTAGTATTCTTCTGGTCCCCACAGACTCAGAGAGA ACCCGCCACCATGCAGAAACCATCATCCAGCTGCCG AATGATAACCCTGCCTGTCCTTTCTGCGGCGATCACGT GGGCAAGCCTTCCGCTCTGAACGTGCACCTGAAGCGC AACCACGGAGGCCGTGAGGTGGAATTCCAGTGTTCTA TGTGCAACAAGGCCGACCCCAAGGCCCACAGCATCCT GTGCCACATCCCTAAGTGTAAAGGAAAGGTGACCGAG GAACCCGCCGGCGAGTGGATTTGTGAAGTGTGCAACA GAGACTTCACCACCAAAATCGGCCTGGGCCAGCATAA GAGATTAGCTCACCCCGCTGTTCGGAATCAGGAGAGA ATCGTCGCCAGCCAGCCTAAGGAAACTAGCAACAGAG GCGCTCACAAAAGATGCTGGACCAAAGAGGAGGAGG AACTGCTGATCAGACTGGAAGCCCAGTTTGAGGGCAA CAAGAACATCAACAAGCTGATCGCAGAACACATCACC ACAAAGACCGCCAAGCAGATCTCCGATAAGAGGAGAC TGCTGAGCCGCAAGCCTGCCGAGGAGCCTAGAGAGGA ACCTGGCACATGCCACCACACCAGACGGGCAGCTGCC AGCCTGAGAACAGAGCCCGAGATGTCCCACCACGCTC AGGCCGAAGATCGGGATAATGGCCCTGGCCGGAGACC CCTGCCTGGTAGAGCCGCCGCCGGCGGCAGAACAATG GACGAGATCAGGAGACACCCTGACAAGGGCAATGGAC AGCAGCGGCCTACCAAACAGAAGTCTGAGGAGCAGCT GCAAGCCTACTACAAAAAGACACTGGAGGAGCGGCTG AGCGCCGGAGCCCTGAATACCTTCCCCCGCGCCTTCAA GCAAGTGATGGAAGGCAGAGATATCAAGCTGGTGATC AACCAGACAGCCCAGGACTGCTTCGGCTGTCTGGAGA GCATCTCTCAGATCAGAACAGCGACCAGAGATAAGAA AGATACCGTGACCAGAGAGAAGCACCCGAAGAAGCC ATTCCAAAAGTGGATGAAGGACCGGGCCATAAAGAAG GGCAACTACCTGAGGTTCCAGAGATTGTTCTACCTGGA CCGAGGCAAACTGGCCAAGATCATCCTGGACGATATC GAATGCCTGAGCTGCGACATCCCCCTGTCTGAGATCTA CAGCGTGTTTAAGACAAGATGGGAAACCACAGGAAGC TTCAAATCTCTGGGCGATTTTAAAACCTACGGCAAGGC CGACAACACTGCCTTCAGAGAACTGATTACAGCTAAG GAAATCGAGAAAAACGTGCAGGAGATGAGCAAGGGC AGCGCCCCTGGCCCTGACGGTATCACCCTGGGAGATG TGGTGAAAATGGACCCCGAGTTCAGCCGCACAATGGA GATCTTCAACCTGTGGCTGACCACTGGCAAAATCCCTG ACATGGTGCGGGGATGCCGCACCGTGCTGATACCTAA GAGCAGCAAGCCAGATAGACTGAAGGATATCAACAAC TGGCGGCCTATCACAATCGGCAGCATCCTGCTCCGGCT GTTTAGCAGAATCGTAACCGCCCGTCTGAGTAAGGCCT GTCCTCTGAACCCCAGACAGCGGGGCTTCATCCGGGC CGCCGGCTGCAGCGAGAACCTCAAACTGCTGCAGACC ATCATCTGGTCGGCCAAACGAGAGCACAGACCTCTCG	Fig. 3

	GCGTGGTGTTCGTGGACATCGCCAAAGCTTTTGATACC<br>GTCTCCCACCAGCACATCATCCACGCCCTGCAGCAGCG<br>GGAAGTGGACCCTCACATCGTGGGACTGGTGTCCAAC<br>ATGTACGAGAACATCAGCACTTATATCACCACCAAGA<br>GAAACACCCACACAGACAAGATCCAGATCAGAGTGGG<br>CGTTAAGCAGGGCGATCCTATGTCTCCTCTGCTGTTCA<br>ACCTGGCTATGGACCCTCTGCTCTGTAAACTGGAGGAG<br>TCTGGCAAGGGATACCACAGAGGCCAAAGCTCCATCA<br>CCGCTATGGCCTTTGCCGACGACCTGGTGCTGCTGTCT<br>GATTCTTGGGAGAACATGAATACCAACATAAGCATCC<br>TGGAAACATTCTGCAACCTTACAGGCCTGAAAACCCA<br>GGGACAAAAGTGCCACGGCTTTTACATCAAGCCTACC<br>AAAGACAGCTACACCATCAACGACTGCGCCGCCTGGA<br>CCATCAACGGCACCCCTTTAAACATGATCGACCCTGGA<br>GAAAGCGAGAAGTATCTGGGCCTGCAGTTCGACCCCT<br>GGATCGGCATCGCTAGAAGCGGCCTGAGCACAAAGCT<br>GGACTTTTGGCTGCAGAGAATTGACCAGGCCCCTCTGA<br>AGCCTCTGCAGAAAACCGACATCCTGAAAACCTACAC<br>AATCCCTCGGCTGATCTACATTGCCGATCACAGCGAGG<br>TAAAGACCGCCCTGCTGGAAACCCTGGACCAAAAGAT<br>CAGAACCGCCGTGAAGGAATGGCTCCACCTGCCCCCC<br>TGCACCTGCGACGCTATCCTGTACAGCAGCACCAGGG<br>ACGGCGGCCTGGGCATCACCAAGCTGGCGGGCCTGAT<br>CCCCTCCGTCCAGGCTAGACGGCTGCATAGAATCGCCC<br>AGAGCAGCGATGACACAATGAAGTGCTTTATGGAAAA<br>GGAAAAGATGGAACAGCTGCACAAGAAGCTGTGGATT<br>CAGGCCGGTGGCGACAGAGAGAACATTCCTAGCATCT<br>GGGAGGCGCCGCCTAGTAGCGAGCCTCCCAACAACGT<br>GTCTACCAATTCTGAGTGGGAAGCCCCTACACAGAAG<br>GACAAGTTCCCTAAGCCTTGTAATTGGAGAAAGAACG<br>AGTTCAAGAAGTGGACAAAGCTGGCCTCTCAGGGCCG<br>GGGAATTGTGAATTTCGAGCGGGACAAGATCAGCAAT<br>CACTGGATTCAGTACTACAGAAGAATCCCACACAGAA<br>AGCTGCTGACGGCCCTTCAGCTGAGAGCCAACGTGTA<br>CCCCACGCGGGAGTTCCTGGCCAGAGGTAGACAGGAC<br>CAGTACATCAAGGCCTGCAGACATTGTGATGCTGATAT<br>CGAGTCTTGCGCCCACATCATCGGCAACTGCCCTGTGA<br>CACAGGACGCGAGAATCAAAAGACACAACTACATCTG<br>CGAGCTGCTGCTGGAAGAGGCCAAGAAGAAGGACTGG<br>GTGGTGTTCAAGGAACCCCACATCAGAGACAGCAATA<br>AGGAACTCTATAAACCTGACCTGATCTTCGTGAAGGA<br>CGCCCGGGCCCTGGTCGTGGACGTGACCGTGAGATAC<br>GAGGCCGCCAAGTCTAGCCTGGAGGAGGCTGCCGCCG<br>AGAAAGTGCGGAAGTACAAGCACCTTGAAACAGAAGT<br>GCGGCACCTGACCAACGCCAAGGACGTCACATTCGTG<br>GGCTTCCCCCTGGGCGCCAGAGGCAAATGGCACCAGG<br>ATAACTTCAAGCTGCTGACAGAGCTGGGCCTGAGCAA<br>GTCCCGCCAAGTGAAGATGGCCGAGACCTTCAGCACC<br>GTGGCCCTGTTCTCCTCTGTGGACATCGTGCACATGTT<br>CGCTAGCAGAGCCAGAAAGAGCATGGTGATGCATATG<br>GGTGGAGGTAGCGGGGGCAGTGGAGGGATGGGGAGC<br>GACTACAAAGACCATGACGGTGATTATAAAGATCATG<br>ACATCGATTACAAGGATGACGATGACAAGAAGTGATG<br>ACCTCGAGCTGGTACTGCATGCACGCAATGCTAGCTGC<br>CCCTTTCCCGTCCTGGGTACCCCGAGTCTCCCCCGACC<br>TCGGGTCCCAGGTATGCTCCCACCTCCACCTGCCCCAC	

	TCACCACCTCTGCTAGTTCCAGACACCTCCCAAGCACG<br>CAGCAATGCAGCTCAAAACGCTTAGCCTAGCCACACC<br>CCCACGGGAAACAGCAGTGATTAACCTTTAGCAATAA<br>ACGAAAGTTTAACTAAGCTATACTAACCCCAGGGTTG<br>GTCAATTTCGTGCCAGCCACACCCTGGAGCTAGCAAA<br>AAAAAAAAAAAAAAAAAAAAAAAAAAAAAAGTCTTCA<br>TCGGAAAGAACATGTGAGCAAAAGGCCAGCAAAAGG<br>CCAGGAACCGTAAAAAGGCCGCGTTGCTGGCGTTTTTC<br>CATAGGCTCCGCCCCCCTGACGAGCATCACAAAAATC<br>GACGCTCAAGTCAGAGGTGGCGAAACCCGACAGGACT<br>ATAAAGATACCAGGCGTTTCCCCCTGGAAGCTCCCTCG<br>TGCGCTCTCCTGTTCCGACCCTGCCGCTTACCGGATAC<br>CTGTCCGCCTTTCTCCCTTCGGGAAGCGTGGCGCTTTC<br>TCATAGCTCACGCTGTAGGTATCTCAGTTCGGTGTAGG<br>TCGTTCGCTCCAAGCTGGGCTGTGTGCACGAACCCCCC<br>GTTCAGCCCGACCGCTGCGCCTTATCCGGTAACTATCG<br>TCTTGAGTCCAACCCGGTAAGACACGACTTATCGCCAC<br>TGGCAGCAGCCACTGGTAACAGGATTAGCAGAGCGAG<br>GTATGTAGGCGGTGCTACAGAGTTCTTGAAGTGGTGG<br>CCTAACTACGGCTACACTAGAAGAACAGTATTTGGTAT<br>CTGCGCTCTGCTGAAGCCAGTTACCTTCGGAAAAAGA<br>GTTGGTAGCTCTTGATCCGGCAAACAAACCACCGCTG<br>GTAGCGGTGGTTTTTTTGTTTGCAAGCAGCAGATTACG<br>CGCAGAAAAAAAGGATCTCAAGAAGATCCTTTGATCT<br>TTTCTACGGGGTCTGACGCTCAGTGGAACGAAAACTC<br>ACGTTAAGGGATTTTGGTCATGAGATTATCAAAAAGG<br>ATCTTCACCTAGATCCTTTTAAATTAAAAATGAAGTTT<br>TAAATCAATCTAAAGTATATATGAGTAAACTTGGTCTG<br>ACAGTTACCAATGCTTAATCAGTGAGGCACCTATCTCA<br>GCGATCTGTCTATTTCGTTCATCCATAGTTGCCTGACTC<br>CCCGTCGTGTAGATAACTACGATACGGGAGGGCTTAC<br>CATCTGGCCCCAGTGCTGCAATGATACCGCGAGACCC<br>ACGCTCACCGGCTCCAGATTTATCAGCAATAAACCAG<br>CCAGCCGGAAGGGCCGAGCGCAGAAGTGGTCCTGCAA<br>CTTTATCCGCCTCCATCCAGTCTATTAATTGTTGCCGG<br>GAAGCTAGAGTAAGTAGTTCGCCAGTTAATAGTTTGC<br>GCAACGTTGTTGCCATTGCTACAGGCATCGTGGTGTCA<br>CGCTCGTCGTTTGGTATGGCTTCATTCAGCTCCGGTTC<br>CCAACGATCAAGGCGAGTTACATGATCCCCCATGTTGT<br>GCAAAAAAGCGGTTAGCTCCTTCGGTCCTCCGATCGTT<br>GTCAGAAGTAAGTTGGCCGCAGTGTTATCACTCATGGT<br>TATGGCAGCACTGCATAATTCTCTTACTGTCATGCCAT<br>CCGTAAGATGCTTTTCTGTGACTGGTGAGTACTCAACC<br>AAGTCATTCTGAGAATAGTGTATGCGGCGACCGAGTT<br>GCTCTTGCCCGGCGTCAATACGGGATAATACCGCGCC<br>ACATAGCAGAACTTTAAAAGTGCTCATCATTGGAAAA<br>CGTTCTTCGGGGCGAAAACTCTCAAGGATCTTACCGCT<br>GTTGAGATCCAGTTCGATGTAACCCACTCGTGCACCCA<br>ACTGATCTTCAGCATCTTTTACTTTCACCAGCGTTTCTG<br>GGTGAGCAAAAACAGGAAGGCAAAATGCCGCAAAAA<br>AGGGAATAAGGGCGACACGGAAATGTTGAATACTCAT<br>ACTCTTCCTTTTTCAATATTATTGAAGCATTTATCAGGG<br>TTATTGTCTCATGAGCGGATACATATTTGAATGTATTT<br>AGAAAAATAAACAAAT	
TaGu ΔCTI	AGGGGTTCCGCGCACATTTCCCCGAAAAGTGCCACCT<br>GACGTCCCAATGATTAATACGACTCACTATAAGGAAT	Fig. 4, Fig. S8

	AAACTAGTATTCTTCTGGTCCCCACAGACTCAGAGAGA<br>ACCCGCCACCATGGAGAAGGTGATGGTCACCGTTCCG<br>GACAAGAACCCTCCATGTCCATGCTGCGGCACCCGGG<br>TGAACAGCGTGCTGAACCTTATCGAGCACCTGAAGGT<br>CAGCCACGGAAAGCGGGGCGTGTGCTTCCGGTGCGCG<br>AAGTGTGGGAAGGAAAATAGCAACTACCACAGCGTGG<br>TGTGCCATTTCCCAAAATGTAGAGGACCTGAGACAGA<br>GAAAGCTCCAGCCGGCGAGTGGATTTGTGAAGTGTGC<br>AACAGAGACTTCACCACCAAAATCGGCCTGGGCCAGC<br>ATAAGAGATTAGCTCACCCCGCTGTTCGGAATCAGGA<br>GAGAATCGTCGCCAGCCAGCCTAAGGAAACTAGCAAC<br>AGAGGCGCTCACAAAAGATGCTGGACCAAAGAGGAG<br>GAGGAACTGCTGATCAGACTGGAAGCCCAGTTTGAGG<br>GCAACAAGAACATCAACAAGCTGATCGCAGAACACAT<br>CACCACAAAGACCGCCAAGCAGATCTCCGATAAGAGG<br>AGACTGCTGAGCCGCAAGCCTGCCGAGGAGCCTAGAG<br>AGGAACCTGGCACATGCCACCACACCAGACGGGCAGC<br>TGCCAGCCTGAGAACAGAGCCCGAGATGTCCCACCAC<br>GCTCAGGCCGAAGATCGGGATAATGGCCCTGGCCGGA<br>GACCCCTGCCTGGTAGAGCCGCCGCCGGCGGCAGAAC<br>AATGGACGAGATCAGGAGACACCCTGACAAGGGCAAT<br>GGACAGCAGCGGCCTACCAAACAGAAGTCTGAGGAGC<br>AGCTGCAAGCCTACTACAAAAAGACACTGGAGGAGCG<br>GCTGAGCGCCGGAGCCCTGAATACCTTCCCCCGCGCCT<br>TCAAGCAAGTGATGGAAGGCAGAGATATCAAGCTGGT<br>GATCAACCAGACAGCCCAGGACTGCTTCGGCTGTCTG<br>GAGAGCATCTCTCAGATCAGAACAGCGACCAGAGATA<br>AGAAAGATACCGTGACCAGAGAGAAGCACCCGAAGA<br>AGCCATTCCAAAAGTGGATGAAGGACCGGGCCATAAA<br>GAAGGGCAACTACCTGAGGTTCCAGAGATTGTTCTAC<br>CTGGACCGAGGCAAACTGGCCAAGATCATCCTGGACG<br>ATATCGAATGCCTGAGCTGCGACATCCCCCTGTCTGAG<br>ATCTACAGCGTGTTTAAGACAAGATGGGAAACCACAG<br>GAAGCTTCAAATCTCTGGGCGATTTTAAAACCTACGGC<br>AAGGCCGACAACACTGCCTTCAGAGAACTGATTACAG<br>CTAAGGAAATCGAGAAAAACGTGCAGGAGATGAGCA<br>AGGGCAGCGCCCCTGGCCCTGACGGTATCACCCTGGG<br>AGATGTGGTGAAAATGGACCCCGAGTTCAGCCGCACA<br>ATGGAGATCTTCAACCTGTGGCTGACCACTGGCAAAA<br>TCCCTGACATGGTGCGGGGATGCCGCACCGTGCTGAT<br>ACCTAAGAGCAGCAAGCCAGATAGACTGAAGGATATC<br>AACAACTGGCGGCCTATCACAATCGGCAGCATCCTGC<br>TCCGGCTGTTTAGCAGAATCGTAACCGCCCGTCTGAGT<br>AAGGCCTGTCCTCTGAACCCCAGACAGCGGGGCTTCA<br>TCCGGGCCGCCGGCTGCAGCGAGAACCTCAAACTGCT<br>GCAGACCATCATCTGGTCGGCCAAACGAGAGCACAGA<br>CCTCTCGGCGTGGTGTTCGTGGACATCGCCAAAGCTTT<br>TGATACCGTCTCCCACCAGCACATCATCCACGCCCTGC<br>AGCAGCGGGAAGTGGACCCTCACATCGTGGGACTGGT<br>GTCCAACATGTACGAGAACATCAGCACTTATATCACC<br>ACCAAGAGAAACACCCACACAGACAAGATCCAGATCA<br>GAGTGGGCGTTAAGCAGGGCGATCCTATGTCTCCTCTG<br>CTGTTCAACCTGGCTATGGACCCTCTGCTCTGTAAACT<br>GGAGGAGTCTGGCAAGGGATACCACAGAGGCCAAAG<br>CTCCATCACCGCTATGGCCTTTGCCGACGACCTGGTGC<br>TGCTGTCTGATTCTTGGGAGAACATGAATACCAACATA	

	AGCATCCTGGAAACATTCTGCAACCTTACAGGCCTGA<br>AAACCCAGGGACAAAAGTGCCACGGCTTTTACATCAA<br>GCCTACCAAAGACAGCTACACCATCAACGACTGCGCC<br>GCCTGGACCATCAACGGCACCCCTTTAAACATGATCG<br>ACCCTGGAGAAAGCGAGAAGTATCTGGGCCTGCAGTT<br>CGACCCCTGGATCGGCATCGCTAGAAGCGGCCTGAGC<br>ACAAAGCTGGACTTTTGGCTGCAGAGAATTGACCAGG<br>CCCCTCTGAAGCCTCTGCAGAAAACCGACATCCTGAA<br>AACCTACACAATCCCTCGGCTGATCTACATTGCCGATC<br>ACAGCGAGGTAAAGACCGCCCTGCTGGAAACCCTGGA<br>CCAAAAGATCAGAACCGCCGTGAAGGAATGGCTCCAC<br>CTGCCCCCCTGCACCTGCGACGCTATCCTGTACAGCAG<br>CACCAGGGACGGCGGCCTGGGCATCACCAAGCTGGCG<br>GGCCTGATCCCCTCCGTCCAGGCTAGACGGCTGCATAG<br>AATCGCCCAGAGCAGCGATGACACAATGAAGTGCTTT<br>ATGGAAAAGGAAAAGATGGAACAGCTGCACAAGAAG<br>CTGTGGATTCAGGCCGGTGGCGACAGAGAGAACATTC<br>CTAAGCCTTGTAATTGGAGAAAGAACGAGTTCAAGAA<br>GTGGACAAAGCTGGCCTCTCAGGGCCGGGGAATTGTG<br>AATTTCGAGCGGGACAAGATCAGCAATCACTGGATTC<br>AGTACTACAGAAGAATCCCACACAGAAAGCTGCTGAC<br>GGCCCTTCAGCTGAGAGCCAACGTGTACCCCACGCGG<br>GAGTTCCTGGCCAGAGGTAGACAGGACCAGTACATCA<br>AGGCCTGCAGACATTGTGATGCTGATATCGAGTCTTGC<br>GCCCACATCATCGGCAACTGCCCTGTGACACAGGACG<br>CGAGAATCAAAAGACACAACTACATCTGCGAGCTGCT<br>GCTGGAAGAGGCCAAGAAGAAGGACTGGGTGGTGTTC<br>AAGGAACCCCACATCAGAGACAGCAATAAGGAACTCT<br>ATAAACCTGACCTGATCTTCGTGAAGGACGCCCGGGC<br>CCTGGTCGTGGACGTGACCGTGAGATACGAGGCCGCC<br>AAGTCTAGCCTGGAGGAGGCTGCCGCCGAGAAAGTGC<br>GGAAGTACAAGCACCTTGAAACAGAAGTGCGGCACCT<br>GACCAACGCCAAGGACGTCACATTCGTGGGCTTCCCC<br>CTGGGCGCCAGAGGCAAATGGCACCAGGATAACTTCA<br>AGCTGCTGACAGAGCTGGGCCTGAGCAAGTCCCGCCA<br>AGTGAAGATGGCCGAGACCTTCAGCACCGTGGCCCTG<br>TTCTCCTCTGTGGACATCGTGCACATGTTCGCTAGCAG<br>AGCCAGAAAGAGCATGGTGATGCATATGGGTGGAGGT<br>AGCGGGGGCAGTGGAGGGATGGGGAGCGACTACAAA<br>GACCATGACGGTGATTATAAAGATCATGACATCGATT<br>ACAAGGATGACGATGACAAGAAGTGATGACCTCGAGC<br>TGGTACTGCATGCACGCAATGCTAGCTGCCCCTTTCCC<br>GTCCTGGGTACCCCGAGTCTCCCCCGACCTCGGGTCCC<br>AGGTATGCTCCCACCTCCACCTGCCCCACTCACCACCT<br>CTGCTAGTTCCAGACACCTCCCAAGCACGCAGCAATG<br>CAGCTCAAAACGCTTAGCCTAGCCACACCCCCACGGG<br>AAACAGCAGTGATTAACCTTTAGCAATAAACGAAAGT<br>TTAACTAAGCTATACTAACCCCAGGGTTGGTCAATTTC<br>GTGCCAGCCACACCCTGGAGCTAGCAAAAAAAAAAAA<br>AAAAAAAAAAAAAAAAAAAAGTCTTCATCGGAAAGA<br>ACATGTGAGCAAAAGGCCAGCAAAAGGCCAGGAACC<br>GTAAAAAGGCCGCGTTGCTGGCGTTTTTCCATAGGCTC<br>CGCCCCCCTGACGAGCATCACAAAAATCGACGCTCAA<br>GTCAGAGGTGGCGAAACCCGACAGGACTATAAAGATA<br>CCAGGCGTTTCCCCCTGGAAGCTCCCTCGTGCGCTCTC<br>CTGTTCCGACCCTGCCGCTTACCGGATACCTGTCCGCC	

	TTTCTCCCTTCGGGAAGCGTGGCGCTTTCTCATAGCTC ACGCTGTAGGTATCTCAGTTCGGTGTAGGTCGTTCGCT CCAAGCTGGGCTGTGTGCACGAACCCCCCGTTCAGCCC GACCGCTGCGCCTTATCCGGTAACTATCGTCTTGAGTC CAACCCGGTAAGACACGACTTATCGCCACTGGCAGCA GCCACTGGTAACAGGATTAGCAGAGCGAGGTATGTAG GCGGTGCTACAGAGTTCTTGAAGTGGTGGCCTAACTAC GGCTACACTAGAAGAACAGTATTTGGTATCTGCGCTCT GCTGAAGCCAGTTACCTTCGGAAAAAGAGTTGGTAGC TCTTGATCCGGCAAACAAACCACCGCTGGTAGCGGTG GTTTTTTTGTTTGCAAGCAGCAGATTACGCGCAGAAAA AAAGGATCTCAAGAAGATCCTTTGATCTTTTCTACGGG GTCTGACGCTCAGTGGAACGAAAACTCACGTTAAGGG ATTTTGGTCATGAGATTATCAAAAAGGATCTTCACCTA GATCCTTTTAAATTAAAAATGAAGTTTTAAATCAATCT AAAGTATATATGAGTAAACTTGGTCTGACAGTTACCA ATGCTTAATCAGTGAGGCACCTATCTCAGCGATCTGTC TATTTCGTTCATCCATAGTTGCCTGACTCCCCGTCGTGT AGATAACTACGATACGGGAGGGCTTACCATCTGGCCC CAGTGCTGCAATGATACCGCGAGACCCACGCTCACCG GCTCCAGATTTATCAGCAATAAACCAGCCAGCCGGAA GGGCCGAGCGCAGAAGTGGTCCTGCAACTTTATCCGC CTCCATCCAGTCTATTAATTGTTGCCGGGAAGCTAGAG TAAGTAGTTCGCCAGTTAATAGTTTGCGCAACGTTGTT GCCATTGCTACAGGCATCGTGGTGTCACGCTCGTCGTT TGGTATGGCTTCATTCAGCTCCGGTTCCCAACGATCAA GGCGAGTTACATGATCCCCCATGTTGTGCAAAAAAGC GGTTAGCTCCTTCGGTCCTCCGATCGTTGTCAGAAGTA AGTTGGCCGCAGTGTTATCACTCATGGTTATGGCAGCA CTGCATAATTCTCTTACTGTCATGCCATCCGTAAGATG CTTTTCTGTGACTGGTGAGTACTCAACCAAGTCATTCT GAGAATAGTGTATGCGGCGACCGAGTTGCTCTTGCCC GGCGTCAATACGGGATAATACCGCGCCACATAGCAGA ACTTTAAAAGTGCTCATCATTGGAAAACGTTCTTCGGG GCGAAAACTCTCAAGGATCTTACCGCTGTTGAGATCCA GTTCGATGTAACCCACTCGTGCACCCAACTGATCTTCA GCATCTTTTACTTTCACCAGCGTTTCTGGGTGAGCAAA AACAGGAAGGCAAAATGCCGCAAAAAAGGGAATAAG GGCGACACGGAAATGTTGAATACTCATACTCTTCCTTT TTCAATATTATTGAAGCATTTATCAGGGTTATTGTCTC ATGAGCGGATACATATTTGAATGTATTTAGAAAAATA AACAAAT	
TaGu PmCTI	AGGGGTTCCGCGCACATTTCCCCGAAAAGTGCCACCT GACGTCCCAATGATTAATACGACTCACTATAAGGAAT AAACTAGTATTCTTCTGGTCCCCACAGACTCAGAGAGA ACCCGCCACCATGGAGAAGGTGATGGTCACCGTTCCG GACAAGAACCCTCCATGTCCATGCTGCGGCACCCGGG TGAACAGCGTGCTGAACCTTATCGAGCACCTGAAGGT CAGCCACGGAAAGCGGGGCGTGTGCTTCCGGTGCGCG AAGTGTGGGAAGGAAAATAGCAACTACCACAGCGTGG TGTGCCATTTCCCAAAATGTAGAGGACCTGAGACAGA GAAAGCTCCAGCCGGCGAGTGGATTTGTGAAGTGTGC AACAGAGACTTCACCACCAAAATCGGCCTGGGCCAGC ATAAGAGATTAGCTCACCCCGCTGTTCGGAATCAGGA GAGAATCGTCGCCAGCCAGCCTAAGGAAACTAGCAAC AGAGGCGCTCACAAAAGATGCTGGACCAAAGAGGAG	Fig. 4

	GAGGAACTGCTGATCAGACTGGAAGCCCAGTTTGAGG<br>GCAACAAGAACATCAACAAGCTGATCGCAGAACACAT<br>CACCACAAAGACCGCCAAGCAGATCTCCGATAAGAGG<br>AGACTGCTGAGCCGCAAGCCTGCCGAGGAGCCTAGAG<br>AGGAACCTGGCACATGCCACCACACCAGACGGGCAGC<br>TGCCAGCCTGAGAACAGAGCCCGAGATGTCCCACCAC<br>GCTCAGGCCGAAGATCGGGATAATGGCCCTGGCCGGA<br>GACCCCTGCCTGGTAGAGCCGCCGCCGGCGGCAGAAC<br>AATGGACGAGATCAGGAGACACCCTGACAAGGGCAAT<br>GGACAGCAGCGGCCTACCAAACAGAAGTCTGAGGAGC<br>AGCTGCAAGCCTACTACAAAAAGACACTGGAGGAGCG<br>GCTGAGCGCCGGAGCCCTGAATACCTTCCCCCGCGCCT<br>TCAAGCAAGTGATGGAAGGCAGAGATATCAAGCTGGT<br>GATCAACCAGACAGCCCAGGACTGCTTCGGCTGTCTG<br>GAGAGCATCTCTCAGATCAGAACAGCGACCAGAGATA<br>AGAAAGATACCGTGACCAGAGAGAAGCACCCGAAGA<br>AGCCATTCCAAAAGTGGATGAAGGACCGGGCCATAAA<br>GAAGGGCAACTACCTGAGGTTCCAGAGATTGTTCTAC<br>CTGGACCGAGGCAAACTGGCCAAGATCATCCTGGACG<br>ATATCGAATGCCTGAGCTGCGACATCCCCCTGTCTGAG<br>ATCTACAGCGTGTTTAAGACAAGATGGGAAACCACAG<br>GAAGCTTCAAATCTCTGGGCGATTTTAAAACCTACGGC<br>AAGGCCGACAACACTGCCTTCAGAGAACTGATTACAG<br>CTAAGGAAATCGAGAAAAACGTGCAGGAGATGAGCA<br>AGGGCAGCGCCCCTGGCCCTGACGGTATCACCCTGGG<br>AGATGTGGTGAAAATGGACCCCGAGTTCAGCCGCACA<br>ATGGAGATCTTCAACCTGTGGCTGACCACTGGCAAAA<br>TCCCTGACATGGTGCGGGGATGCCGCACCGTGCTGAT<br>ACCTAAGAGCAGCAAGCCAGATAGACTGAAGGATATC<br>AACAACTGGCGGCCTATCACAATCGGCAGCATCCTGC<br>TCCGGCTGTTTAGCAGAATCGTAACCGCCCGTCTGAGT<br>AAGGCCTGTCCTCTGAACCCCAGACAGCGGGGCTTCA<br>TCCGGGCCGCCGGCTGCAGCGAGAACCTCAAACTGCT<br>GCAGACCATCATCTGGTCGGCCAAACGAGAGCACAGA<br>CCTCTCGGCGTGGTGTTCGTGGACATCGCCAAAGCTTT<br>TGATACCGTCTCCCACCAGCACATCATCCACGCCCTGC<br>AGCAGCGGGAAGTGGACCCTCACATCGTGGGACTGGT<br>GTCCAACATGTACGAGAACATCAGCACTTATATCACC<br>ACCAAGAGAAACACCCACACAGACAAGATCCAGATCA<br>GAGTGGGCGTTAAGCAGGGCGATCCTATGTCTCCTCTG<br>CTGTTCAACCTGGCTATGGACCCTCTGCTCTGTAAACT<br>GGAGGAGTCTGGCAAGGGATACCACAGAGGCCAAAG<br>CTCCATCACCGCTATGGCCTTTGCCGACGACCTGGTGC<br>TGCTGTCTGATTCTTGGGAGAACATGAATACCAACATA<br>AGCATCCTGGAAACATTCTGCAACCTTACAGGCCTGA<br>AAACCCAGGGACAAAAGTGCCACGGCTTTTACATCAA<br>GCCTACCAAAGACAGCTACACCATCAACGACTGCGCC<br>GCCTGGACCATCAACGGCACCCCTTTAAACATGATCG<br>ACCCTGGAGAAAGCGAGAAGTATCTGGGCCTGCAGTT<br>CGACCCCTGGATCGGCATCGCTAGAAGCGGCCTGAGC<br>ACAAAGCTGGACTTTTGGCTGCAGAGAATTGACCAGG<br>CCCCTCTGAAGCCTCTGCAGAAAACCGACATCCTGAA<br>AACCTACACAATCCCTCGGCTGATCTACATTGCCGATC<br>ACAGCGAGGTAAAGACCGCCCTGCTGGAAACCCTGGA<br>CCAAAAGATCAGAACCGCCGTGAAGGAATGGCTCCAC<br>CTGCCCCCCTGCACCTGCGACGCTATCCTGTACAGCAG	

	CACCAGGGACGGCGGCCTGGGCATCACCAAGCTGGCG<br>GGCCTGATCCCCTCCGTCCAGGCTAGACGGCTGCATAG<br>AATCGCCCAGAGCAGCGATGACACAATGAAGTGCTTT<br>ATGGAAAAGGAAAAGATGGAACAGCTGCACAAGAAG<br>CTGTGGATTCAGGCCGGCGGCAAGAAGGAAGAGATCC<br>CCAGAATCACCGACCCGGTGTCCATCGACTACAGACT<br>GCCAAGACGGATTCTGGAACTGCTGAATGAGTGGGAG<br>AAGCCAGCTCCAAAGAAGATGTACCCCATCCCTTGCA<br>ACTGGAGAAAGAACGAGTTCAAGAAGTGGACAAAGCT<br>GGCCTCTCAGGGCCGGGGAATTGTGAATTTCGAGCGG<br>GACAAGATCAGCAATCACTGGATTCAGTACTACAGAA<br>GAATCCCACACAGAAAGCTGCTGACGGCCCTTCAGCT<br>GAGAGCCAACGTGTACCCCACGCGGGAGTTCCTGGCC<br>AGAGGTAGACAGGACCAGTACATCAAGGCCTGCAGAC<br>ATTGTGATGCTGATATCGAGTCTTGCGCCCACATCATC<br>GGCAACTGCCCTGTGACACAGGACGCGAGAATCAAAA<br>GACACAACTACATCTGCGAGCTGCTGCTGGAAGAGGC<br>CAAGAAGAAGGACTGGGTGGTGTTCAAGGAACCCCAC<br>ATCAGAGACAGCAATAAGGAACTCTATAAACCTGACC<br>TGATCTTCGTGAAGGACGCCCGGGCCCTGGTCGTGGA<br>CGTGACCGTGAGATACGAGGCCGCCAAGTCTAGCCTG<br>GAGGAGGCTGCCGCCGAGAAAGTGCGGAAGTACAAG<br>CACCTTGAAACAGAAGTGCGGCACCTGACCAACGCCA<br>AGGACGTCACATTCGTGGGCTTCCCCCTGGGCGCCAG<br>AGGCAAATGGCACCAGGATAACTTCAAGCTGCTGACA<br>GAGCTGGGCCTGAGCAAGTCCCGCCAAGTGAAGATGG<br>CCGAGACCTTCAGCACCGTGGCCCTGTTCTCCTCTGTG<br>GACATCGTGCACATGTTCGCTAGCAGAGCCAGAAAGA<br>GCATGGTGATGCATATGGGTGGAGGTAGCGGGGGCAG<br>TGGAGGGATGGGGAGCGACTACAAAGACCATGACGGT<br>GATTATAAAGATCATGACATCGATTACAAGGATGACG<br>ATGACAAGAAGTGATGACCTCGAGCTGGTACTGCATG<br>CACGCAATGCTAGCTGCCCCTTTCCCGTCCTGGGTACC<br>CCGAGTCTCCCCCGACCTCGGGTCCCAGGTATGCTCCC<br>ACCTCCACCTGCCCCACTCACCACCTCTGCTAGTTCCA<br>GACACCTCCCAAGCACGCAGCAATGCAGCTCAAAACG<br>CTTAGCCTAGCCACACCCCCACGGGAAACAGCAGTGA<br>TTAACCTTTAGCAATAAACGAAAGTTTAACTAAGCTAT<br>ACTAACCCCAGGGTTGGTCAATTTCGTGCCAGCCACAC<br>CCTGGAGCTAGCAAAAAAAAAAAAAAAAAAAAAAAAA<br>AAAAAAAAGTCTTCATCGGAAAGAACATGTGAGCAAA<br>AGGCCAGCAAAAGGCCAGGAACCGTAAAAAGGCCGC<br>GTTGCTGGCGTTTTTCCATAGGCTCCGCCCCCCTGACG<br>AGCATCACAAAAATCGACGCTCAAGTCAGAGGTGGCG<br>AAACCCGACAGGACTATAAAGATACCAGGCGTTTCCC<br>CCTGGAAGCTCCCTCGTGCGCTCTCCTGTTCCGACCCT<br>GCCGCTTACCGGATACCTGTCCGCCTTTCTCCCTTCGG<br>GAAGCGTGGCGCTTTCTCATAGCTCACGCTGTAGGTAT<br>CTCAGTTCGGTGTAGGTCGTTCGCTCCAAGCTGGGCTG<br>TGTGCACGAACCCCCCGTTCAGCCCGACCGCTGCGCCT<br>TATCCGGTAACTATCGTCTTGAGTCCAACCCGGTAAGA<br>CACGACTTATCGCCACTGGCAGCAGCCACTGGTAACA<br>GGATTAGCAGAGCGAGGTATGTAGGCGGTGCTACAGA<br>GTTCTTGAAGTGGTGGCCTAACTACGGCTACACTAGAA<br>GAACAGTATTTGGTATCTGCGCTCTGCTGAAGCCAGTT<br>ACCTTCGGAAAAAGAGTTGGTAGCTCTTGATCCGGCA	

	AACAAACCACCGCTGGTAGCGGTGGTTTTTTTGTTTGC AAGCAGCAGATTACGCGCAGAAAAAAAGGATCTCAAG AAGATCCTTTGATCTTTTCTACGGGGTCTGACGCTCAG TGGAACGAAAACTCACGTTAAGGGATTTTGGTCATGA GATTATCAAAAAGGATCTTCACCTAGATCCTTTTAAAT TAAAAATGAAGTTTTAAATCAATCTAAAGTATATATGA GTAAACTTGGTCTGACAGTTACCAATGCTTAATCAGTG AGGCACCTATCTCAGCGATCTGTCTATTTCGTTCATCC ATAGTTGCCTGACTCCCCGTCGTGTAGATAACTACGAT ACGGGAGGGCTTACCATCTGGCCCCAGTGCTGCAATG ATACCGCGAGACCCACGCTCACCGGCTCCAGATTTATC AGCAATAAACCAGCCAGCCGGAAGGGCCGAGCGCAG AAGTGGTCCTGCAACTTTATCCGCCTCCATCCAGTCTA TTAATTGTTGCCGGGAAGCTAGAGTAAGTAGTTCGCCA GTTAATAGTTTGCGCAACGTTGTTGCCATTGCTACAGG CATCGTGGTGTCACGCTCGTCGTTTGGTATGGCTTCAT TCAGCTCCGGTTCCCAACGATCAAGGCGAGTTACATG ATCCCCCATGTTGTGCAAAAAAGCGGTTAGCTCCTTCG GTCCTCCGATCGTTGTCAGAAGTAAGTTGGCCGCAGTG TTATCACTCATGGTTATGGCAGCACTGCATAATTCTCT TACTGTCATGCCATCCGTAAGATGCTTTTCTGTGACTG GTGAGTACTCAACCAAGTCATTCTGAGAATAGTGTATG CGGCGACCGAGTTGCTCTTGCCCGGCGTCAATACGGG ATAATACCGCGCCACATAGCAGAACTTTAAAAGTGCT CATCATTGGAAAACGTTCTTCGGGGCGAAAACTCTCA AGGATCTTACCGCTGTTGAGATCCAGTTCGATGTAACC CACTCGTGCACCCAACTGATCTTCAGCATCTTTTACTTT CACCAGCGTTTCTGGGTGAGCAAAAACAGGAAGGCAA AATGCCGCAAAAAAGGGAATAAGGGCGACACGGAAA TGTTGAATACTCATACTCTTCCTTTTTCAATATTATTGA AGCATTTATCAGGGTTATTGTCTCATGAGCGGATACAT ATTTGAATGTATTTAGAAAAATAAACAAAT	
PlaMe ΔCTI	AGGGGTTCCGCGCACATTTCCCCGAAAAGTGCCACCT GACGTCCCAATGATTAATACGACTCACTATAAGGAAT AAACTAGTATTCTTCTGGTCCCCACAGACTCAGAGAGA ACCCGCCACCATGCAGAAAACCATCATCCAGCTGCCG AATGATAACCCTGCCTGTCCTTTCTGCGGCGATCACGT GGGCAAGCCTTCCGCTCTGAACGTGCACCTGAAGCGC AACCACGGAGGCCGTGAGGTGGAATTCCAGTGTTCTA TGTGCAACAAGGCCGACCCCAAGGCCCACAGCATCCT GTGCCACATCCCTAAGTGTAAAGGAAAGGTGACCGAG GAACCCACCGGCGATTGGGCCTGCGAGACATGTAACA AGCAGTTCAACACCAAGAGCGGCCTGTCCCAGCACAA GAGAATCGCCCATCCCGCTATCCGGAACCAGGAGAGA ATCGCCGCCAGCCAGCCTAAGCCTAACTCTCAAAGAG GAAAGCACAACAGCTGCTGGACGGTGGAAGAAGAAC AGCTGCTGGCCGCTTTCAACAACATGTTCTGGGGCAAG AAAAATATCAATATCCTGATCTCTGATCACATCCACAT GAAAACAGCCAAGCAGATCAGCGAGAAGAGACGGCT GCTGGGACTGAACAAGAACGCGACAGTGACAACCACA AACCCCCTGCCTGTATCCAGCACCTGTCACCTGAAGAT CCGGACCGACTCCCCTAATACCACCACCGGCCTGAAG GATACCTACATGTGCAAGATCAACGAGAACATCGTGA ACCAGGGCCAGATCAAGTTCGATTCTGAGGTTATCAG CGCCTGGATGGCAGGCGACTCTAATATCCGGAGCCTG GTTGAGAGCACTAGCCTGGACATCCTGAGCACATTCCT	Fig. 4

	GATGGAAACACCTAAGCCTAGAAAGAAAGGCAACAA<br>CAAAATCACAAATAAAAAGAGCGGCAAGAAGAAGAA<br>ATGGATGGAAAAGAGAGCCGTGAAAAAAGGATTCTAC<br>AAAAGATACCAACATCTGTTCGAGACAGATAGATGCA<br>AACTGGCAAGCATCATTCTGGATGGCACCGAGCGACT<br>CCAGTGCCAGATTCCTCTGACAGAGATCCTGGAAACA<br>TATAAGTCTAAGTGGGAGACTCTGACTCCATTCGAGG<br>GCCTCGGCCAATTTAAGAGCCACGCCGTGGCCGACAA<br>CACCGCCTTCGAGATTCTGCTGAGCGCCAAGGAAATC<br>ATGAAGAACATCAAGGAGATGAACAAGAACAGCGCC<br>CCAGGCCCTGATAAGGTGAGCCTGAGAGATCTGCTTCT<br>GGCCGACCCCGAATGCAACGCCCTGGAAAAGCTGTTC<br>AACACCTGGCTGATCACCGGAATCATTCCAAACAGCA<br>TAAAAGAATGTAGAAGCCTGCTGATCCCTAAGACGGC<br>GGACCCCGAGGCCCTGAAGGAACTGGGAAATTGGCGG<br>CCTCTGACCATCGGCAGCATCGTGCTGCGGCTGTTTAG<br>CAGAATCATCACCAACAGACTGGCTAAGGCCTGCCCC<br>ATCAACGCCCGGCAGAGAGGCTTCATCGCCACCCCTG<br>GTTGTAGCGAGAACCTGAAGATTCTTCACACAATCGTT<br>AAACAAGCCAAGACCTCCAAAAAGAGCCTGGGAGTGG<br>TGTTCGTGGACATCGCCAAGGCCTTCGACTCAGTGAGC<br>CACGACCACATTATGTGGGTGCTGCAGGAGCGGGGAC<br>TCGACCAGCACATCGTGAACATTATCGAGGACTCTTAT<br>AAGAAGATCCACACCAGAATGGAGGTGGGCACCGAG<br>AGAACCCCCCCTATCGAGATCAAGGTGGGCGTGAAAC<br>AAGGGGACCCCATGAGCCCTCTGCTGTTTAACCTGGCC<br>ATCGACCCTCTGATCACAGCCCTGGAGAAAGCTAATA<br>CCGGCTTTAGCTACGGCAAGAATAAGATCACCTCTCTG<br>GCCTTCGCGGATGACCTGGTGATGCTGTCCGACACCTG<br>GGAGGGCATGAACAAGAATATCCAGATCCTGGAAACA<br>TTTTGCAATCTGAGTGGCCTGAAGGTCCAGGCTAAGA<br>AATGCTACGGCTTCTTCCTGAGCCCTACCCACGATTCA<br>TATACTATCAACAAATGCGACGCCTGGAAGATCGACA<br>AGGACAGCCTGAACATGATCCAGCCTGGAGAATCTGA<br>GAAGTACCTGGGCCTGAAGGTGGACCCTTGGATCGGC<br>TTCAGCAAGCCCGTGCTGGCCGAAAAGCTTACAATCT<br>GGCTGAAGCGGCTGACCGAAGCCCCTCTGAAGCCTAG<br>CCAGAAACTGACAATGCTAAACATCTACACCATCCCG<br>AGAATCATATACCTGGCCGATCACACCGACACCAAGA<br>AAACCCTGCTGAGCAGCCTTGACGACAACATCAGGAC<br>GGTGGTGAAGGGCTGGCTGCACCTGCCTCCTGACACA<br>TGCAACGGCTTCATCTACACCAAAACTCGGGACGGCG<br>GCCTGGGCGTGACCAGACTGGCTTCTCTTATCCCCAGC<br>ATCCAGGCCAGACGGCTGCACCGGATCGCCACCAGCG<br>AGGACGAGACAATCCGGAACATTGCTATGGCCAACAA<br>TATCGAGGAAGAGTTCCAAAACCTGTGGGTGACCGCC<br>GGCGGCAAGAAGGAAGAGATCCCCATCCCTTGCAACT<br>GGCGGGAAGCCGAGATGGCCCACTGGAAGAACCTGCC<br>TTGCCAGGGATCAGGCATCGAGCACTTCGACAATGAC<br>ACCATCTCCAACGACTGGCTGCAGTTTCACCGGGGCTT<br>CTCCGAGCGACAGTTCCTGATGGGCCTTAAGATCAGA<br>GCCAACGTGTACCCTACCCGGGAGTACCAGGGCAGAG<br>GCAGAACAAACAAGAACGTGAATTGTAGAAATTGCAC<br>CGCCTCTTACGAGAGCCTGTCTCATATCCTGGGCCAGT<br>GCCCTGCCGTGCAGGGCGCTAGAATCCGGCGGCACAA<br>CAAGCTGTGCAGCATGCTGAAGCGGGAGGCCAAGGAA	

	CTGAAGTGGGTCGTGTACGAGGAACCTCACCTACATA<br>CAACAGAAAAAGAGCTGAGAAAGCCTGACCTGATCTT<br>CGTGAAGGAGGAAATGGCCCTGGTGGTCGATGTGACA<br>GTGCGGTTTGAGTACAAGGAAAAGGTGTTCGAGGATG<br>CCGCTGCTGAGAAAGTGCGGCACTACAAGGACCTGAC<br>CAGCCAGATCAAGGAGCTGACCGGCGCCAAAGAGATC<br>GAGTACTTCGGCTTCCCCCTGGGCGCCAGAGGAAAGT<br>GGCCTGAGATCAACGAGAAGGTGCTGACAGCCCTCGG<br>CATGCCTGATTACCAGCAGAAGCGCACCGCCAAACGG<br>TTCAGCAAGAGAACCCTGCTGTACAGCATCGACGTGA<br>TCAACACCTTTGAGAACATCGGCAAGAACAACAAGAA<br>CAACGTCCCCCATATGGGTGGAGGTAGCGGGGGCAGT<br>GGAGGGATGGGGAGCGACTACAAAGACCATGACGGT<br>GATTATAAAGATCATGACATCGATTACAAGGATGACG<br>ATGACAAGAAGTGATGACCTCGAGCTGGTACTGCATG<br>CACGCAATGCTAGCTGCCCCTTTCCCGTCCTGGGTACC<br>CCGAGTCTCCCCCGACCTCGGGTCCCAGGTATGCTCCC<br>ACCTCCACCTGCCCCACTCACCACCTCTGCTAGTTCCA<br>GACACCTCCCAAGCACGCAGCAATGCAGCTCAAAACG<br>CTTAGCCTAGCCACACCCCCACGGGAAACAGCAGTGA<br>TTAACCTTTAGCAATAAACGAAAGTTTAACTAAGCTAT<br>ACTAACCCCAGGGTTGGTCAATTTCGTGCCAGCCACAC<br>CCTGGAGCTAGCAAAAAAAAAAAAAAAAAAAAAAAAA<br>AAAAAAAAGTCTTCATCGGAAAGAACATGTGAGCAAA<br>AGGCCAGCAAAAGGCCAGGAACCGTAAAAAGGCCGC<br>GTTGCTGGCGTTTTTCCATAGGCTCCGCCCCCCTGACG<br>AGCATCACAAAAATCGACGCTCAAGTCAGAGGTGGCG<br>AAACCCGACAGGACTATAAAGATACCAGGCGTTTCCC<br>CCTGGAAGCTCCCTCGTGCGCTCTCCTGTTCCGACCCT<br>GCCGCTTACCGGATACCTGTCCGCCTTTCTCCCTTCGG<br>GAAGCGTGGCGCTTTCTCATAGCTCACGCTGTAGGTAT<br>CTCAGTTCGGTGTAGGTCGTTCGCTCCAAGCTGGGCTG<br>TGTGCACGAACCCCCCGTTCAGCCCGACCGCTGCGCCT<br>TATCCGGTAACTATCGTCTTGAGTCCAACCCGGTAAGA<br>CACGACTTATCGCCACTGGCAGCAGCCACTGGTAACA<br>GGATTAGCAGAGCGAGGTATGTAGGCGGTGCTACAGA<br>GTTCTTGAAGTGGTGGCCTAACTACGGCTACACTAGAA<br>GAACAGTATTTGGTATCTGCGCTCTGCTGAAGCCAGTT<br>ACCTTCGGAAAAAGAGTTGGTAGCTCTTGATCCGGCA<br>AACAAACCACCGCTGGTAGCGGTGGTTTTTTTGTTTGC<br>AAGCAGCAGATTACGCGCAGAAAAAAAGGATCTCAAG<br>AAGATCCTTTGATCTTTTCTACGGGGTCTGACGCTCAG<br>TGGAACGAAAACTCACGTTAAGGGATTTTGGTCATGA<br>GATTATCAAAAAGGATCTTCACCTAGATCCTTTTAAAT<br>TAAAAATGAAGTTTTAAATCAATCTAAAGTATATATGA<br>GTAAACTTGGTCTGACAGTTACCAATGCTTAATCAGTG<br>AGGCACCTATCTCAGCGATCTGTCTATTTCGTTCATCC<br>ATAGTTGCCTGACTCCCCGTCGTGTAGATAACTACGAT<br>ACGGGAGGGCTTACCATCTGGCCCCAGTGCTGCAATG<br>ATACCGCGAGACCCACGCTCACCGGCTCCAGATTTATC<br>AGCAATAAACCAGCCAGCCGGAAGGGCCGAGCGCAG<br>AAGTGGTCCTGCAACTTTATCCGCCTCCATCCAGTCTA<br>TTAATTGTTGCCGGGAAGCTAGAGTAAGTAGTTCGCCA<br>GTTAATAGTTTGCGCAACGTTGTTGCCATTGCTACAGG<br>CATCGTGGTGTCACGCTCGTCGTTTGGTATGGCTTCAT<br>TCAGCTCCGGTTCCCAACGATCAAGGCGAGTTACATG	

	ATCCCCCATGTTGTGCAAAAAAGCGGTTAGCTCCTTCG GTCCTCCGATCGTTGTCAGAAGTAAGTTGGCCGCAGTG TTATCACTCATGGTTATGGCAGCACTGCATAATTCTCT TACTGTCATGCCATCCGTAAGATGCTTTTCTGTGACTG GTGAGTACTCAACCAAGTCATTCTGAGAATAGTGTATG CGGCGACCGAGTTGCTCTTGCCCGGCGTCAATACGGG ATAATACCGCGCCACATAGCAGAACTTTAAAAGTGCT CATCATTGGAAAACGTTCTTCGGGGCGAAAACTCTCA AGGATCTTACCGCTGTTGAGATCCAGTTCGATGTAACC CACTCGTGCACCCAACTGATCTTCAGCATCTTTTACTTT CACCAGCGTTTCTGGGTGAGCAAAAACAGGAAGGCAA AATGCCGCAAAAAAGGGAATAAGGGCGACACGGAAA TGTTGAATACTCATACTCTTCCTTTTTCAATATTATTGA AGCATTTATCAGGGTTATTGTCTCATGAGCGGATACAT ATTTGAATGTATTTAGAAAAATAAACAAAT	
PlaMe TgCTI	AGGGGTTCCGCGCACATTTCCCCGAAAAGTGCCACCT GACGTCCCAATGATTAATACGACTCACTATAAGGAAT AAACTAGTATTCTTCTGGTCCCCACAGACTCAGAGAGA ACCCGCCACCATGCAGAAAACCATCATCCAGCTGCCG AATGATAACCCTGCCTGTCCTTTCTGCGGCGATCACGT GGGCAAGCCTTCCGCTCTGAACGTGCACCTGAAGCGC AACCACGGAGGCCGTGAGGTGGAATTCCAGTGTTCTA TGTGCAACAAGGCCGACCCCAAGGCCCACAGCATCCT GTGCCACATCCCTAAGTGTAAAGGAAAGGTGACCGAG GAACCCACCGGCGATTGGGCCTGCGAGACATGTAACA AGCAGTTCAACACCAAGAGCGGCCTGTCCCAGCACAA GAGAATCGCCCATCCCGCTATCCGGAACCAGGAGAGA ATCGCCGCCAGCCAGCCTAAGCCTAACTCTCAAAGAG GAAAGCACAACAGCTGCTGGACGGTGGAAGAAGAAC AGCTGCTGGCCGCTTTCAACAACATGTTCTGGGGCAAG AAAAATATCAATATCCTGATCTCTGATCACATCCACAT GAAAACAGCCAAGCAGATCAGCGAGAAGAGACGGCT GCTGGGACTGAACAAGAACGCGACAGTGACAACCACA AACCCCCTGCCTGTATCCAGCACCTGTCACCTGAAGAT CCGGACCGACTCCCCTAATACCACCACCGGCCTGAAG GATACCTACATGTGCAAGATCAACGAGAACATCGTGA ACCAGGGCCAGATCAAGTTCGATTCTGAGGTTATCAG CGCCTGGATGGCAGGCGACTCTAATATCCGGAGCCTG GTTGAGAGCACTAGCCTGGACATCCTGAGCACATTCCT GATGGAAACACCTAAGCCTAGAAAGAAAGGCAACAA CAAAATCACAAATAAAAAGAGCGGCAAGAAGAAGAA ATGGATGGAAAAGAGAGCCGTGAAAAAAGGATTCTAC AAAAGATACCAACATCTGTTCGAGACAGATAGATGCA AACTGGCAAGCATCATTCTGGATGGCACCGAGCGACT CCAGTGCCAGATTCCTCTGACAGAGATCCTGGAAACA TATAAGTCTAAGTGGGAGACTCTGACTCCATTCGAGG GCCTCGGCCAATTTAAGAGCCACGCCGTGGCCGACAA CACCGCCTTCGAGATTCTGCTGAGCGCCAAGGAAATC ATGAAGAACATCAAGGAGATGAACAAGAACAGCGCC CCAGGCCCTGATAAGGTGAGCCTGAGAGATCTGCTTCT GGCCGACCCCGAATGCAACGCCCTGGAAAAGCTGTTC AACACCTGGCTGATCACCGGAATCATTCCAAACAGCA TAAAAGAATGTAGAAGCCTGCTGATCCCTAAGACGGC GGACCCCGAGGCCCTGAAGGAACTGGGAAATTGGCGG CCTCTGACCATCGGCAGCATCGTGCTGCGGCTGTTTAG CAGAATCATCACCAACAGACTGGCTAAGGCCTGCCCC	Fig. 4

	ATCAACGCCCGGCAGAGAGGCTTCATCGCCACCCCTG<br>GTTGTAGCGAGAACCTGAAGATTCTTCACACAATCGTT<br>AAACAAGCCAAGACCTCCAAAAAGAGCCTGGGAGTGG<br>TGTTCGTGGACATCGCCAAGGCCTTCGACTCAGTGAGC<br>CACGACCACATTATGTGGGTGCTGCAGGAGCGGGGAC<br>TCGACCAGCACATCGTGAACATTATCGAGGACTCTTAT<br>AAGAAGATCCACACCAGAATGGAGGTGGGCACCGAG<br>AGAACCCCCCCTATCGAGATCAAGGTGGGCGTGAAAC<br>AAGGGGACCCCATGAGCCCTCTGCTGTTTAACCTGGCC<br>ATCGACCCTCTGATCACAGCCCTGGAGAAAGCTAATA<br>CCGGCTTTAGCTACGGCAAGAATAAGATCACCTCTCTG<br>GCCTTCGCGGATGACCTGGTGATGCTGTCCGACACCTG<br>GGAGGGCATGAACAAGAATATCCAGATCCTGGAAACA<br>TTTTGCAATCTGAGTGGCCTGAAGGTCCAGGCTAAGA<br>AATGCTACGGCTTCTTCCTGAGCCCTACCCACGATTCA<br>TATACTATCAACAAATGCGACGCCTGGAAGATCGACA<br>AGGACAGCCTGAACATGATCCAGCCTGGAGAATCTGA<br>GAAGTACCTGGGCCTGAAGGTGGACCCTTGGATCGGC<br>TTCAGCAAGCCCGTGCTGGCCGAAAAGCTTACAATCT<br>GGCTGAAGCGGCTGACCGAAGCCCCTCTGAAGCCTAG<br>CCAGAAACTGACAATGCTAAACATCTACACCATCCCG<br>AGAATCATATACCTGGCCGATCACACCGACACCAAGA<br>AAACCCTGCTGAGCAGCCTTGACGACAACATCAGGAC<br>GGTGGTGAAGGGCTGGCTGCACCTGCCTCCTGACACA<br>TGCAACGGCTTCATCTACACCAAAACTCGGGACGGCG<br>GCCTGGGCGTGACCAGACTGGCTTCTCTTATCCCCAGC<br>ATCCAGGCCAGACGGCTGCACCGGATCGCCACCAGCG<br>AGGACGAGACAATCCGGAACATTGCTATGGCCAACAA<br>TATCGAGGAAGAGTTCCAAAACCTGTGGGTGACCGCC<br>GGTGGCGACAGAGAGAACATTCCTAGCATCTGGGAGG<br>CGCCGCCTAGTAGCGAGCCTCCCAACAACGTGTCTACC<br>AATTCTGAGTGGGAAGCCCCTACACAGAAGGACAAGT<br>TCCCTAAGCCTTGTAATTGGCGGGAAGCCGAGATGGC<br>CCACTGGAAGAACCTGCCTTGCCAGGGATCAGGCATC<br>GAGCACTTCGACAATGACACCATCTCCAACGACTGGC<br>TGCAGTTTCACCGGGGCTTCTCCGAGCGACAGTTCCTG<br>ATGGGCCTTAAGATCAGAGCCAACGTGTACCCTACCC<br>GGGAGTACCAGGGCAGAGGCAGAACAAACAAGAACG<br>TGAATTGTAGAAATTGCACCGCCTCTTACGAGAGCCTG<br>TCTCATATCCTGGGCCAGTGCCCTGCCGTGCAGGGCGC<br>TAGAATCCGGCGGCACAACAAGCTGTGCAGCATGCTG<br>AAGCGGGAGGCCAAGGAACTGAAGTGGGTCGTGTACG<br>AGGAACCTCACCTACATACAACAGAAAAAGAGCTGAG<br>AAAGCCTGACCTGATCTTCGTGAAGGAGGAAATGGCC<br>CTGGTGGTCGATGTGACAGTGCGGTTTGAGTACAAGG<br>AAAAGGTGTTCGAGGATGCCGCTGCTGAGAAAGTGCG<br>GCACTACAAGGACCTGACCAGCCAGATCAAGGAGCTG<br>ACCGGCGCCAAAGAGATCGAGTACTTCGGCTTCCCCCT<br>GGGCGCCAGAGGAAAGTGGCCTGAGATCAACGAGAA<br>GGTGCTGACAGCCCTCGGCATGCCTGATTACCAGCAG<br>AAGCGCACCGCCAAACGGTTCAGCAAGAGAACCCTGC<br>TGTACAGCATCGACGTGATCAACACCTTTGAGAACATC<br>GGCAAGAACAACAAGAACAACGTCCCCCATATGGGTG<br>GAGGTAGCGGGGGCAGTGGAGGGATGGGGAGCGACT<br>ACAAAGACCATGACGGTGATTATAAAGATCATGACAT<br>CGATTACAAGGATGACGATGACAAGAAGTGATGACCT	

	CGAGCTGGTACTGCATGCACGCAATGCTAGCTGCCCCT<br>TTCCCGTCCTGGGTACCCCGAGTCTCCCCCGACCTCGG<br>GTCCCAGGTATGCTCCCACCTCCACCTGCCCCACTCAC<br>CACCTCTGCTAGTTCCAGACACCTCCCAAGCACGCAGC<br>AATGCAGCTCAAAACGCTTAGCCTAGCCACACCCCCA<br>CGGGAAACAGCAGTGATTAACCTTTAGCAATAAACGA<br>AAGTTTAACTAAGCTATACTAACCCCAGGGTTGGTCAA<br>TTTCGTGCCAGCCACACCCTGGAGCTAGCAAAAAAA<br>AAAAAAAAAAAAAAAAAAAAAAAAAGTCTTCATCGGA<br>AAGAACATGTGAGCAAAAGGCCAGCAAAAGGCCAGG<br>AACCGTAAAAAGGCCGCGTTGCTGGCGTTTTTCCATAG<br>GCTCCGCCCCCCTGACGAGCATCACAAAAATCGACGC<br>TCAAGTCAGAGGTGGCGAAACCCGACAGGACTATAAA<br>GATACCAGGCGTTTCCCCCTGGAAGCTCCCTCGTGCGC<br>TCTCCTGTTCCGACCCTGCCGCTTACCGGATACCTGTC<br>CGCCTTTCTCCCTTCGGGAAGCGTGGCGCTTTCTCATA<br>GCTCACGCTGTAGGTATCTCAGTTCGGTGTAGGTCGTT<br>CGCTCCAAGCTGGGCTGTGTGCACGAACCCCCCGTTCA<br>GCCCGACCGCTGCGCCTTATCCGGTAACTATCGTCTTG<br>AGTCCAACCCGGTAAGACACGACTTATCGCCACTGGC<br>AGCAGCCACTGGTAACAGGATTAGCAGAGCGAGGTAT<br>GTAGGCGGTGCTACAGAGTTCTTGAAGTGGTGGCCTA<br>ACTACGGCTACACTAGAAGAACAGTATTTGGTATCTGC<br>GCTCTGCTGAAGCCAGTTACCTTCGGAAAAAGAGTTG<br>GTAGCTCTTGATCCGGCAAACAAACCACCGCTGGTAG<br>CGGTGGTTTTTTTGTTTGCAAGCAGCAGATTACGCGCA<br>GAAAAAAAGGATCTCAAGAAGATCCTTTGATCTTTTCT<br>ACGGGGTCTGACGCTCAGTGGAACGAAAACTCACGTT<br>AAGGGATTTTGGTCATGAGATTATCAAAAAGGATCTTC<br>ACCTAGATCCTTTTAAATTAAAAATGAAGTTTTAAATC<br>AATCTAAAGTATATATGAGTAAACTTGGTCTGACAGTT<br>ACCAATGCTTAATCAGTGAGGCACCTATCTCAGCGATC<br>TGTCTATTTCGTTCATCCATAGTTGCCTGACTCCCCGTC<br>GTGTAGATAACTACGATACGGGAGGGCTTACCATCTG<br>GCCCCAGTGCTGCAATGATACCGCGAGACCCACGCTC<br>ACCGGCTCCAGATTTATCAGCAATAAACCAGCCAGCC<br>GGAAGGGCCGAGCGCAGAAGTGGTCCTGCAACTTTAT<br>CCGCCTCCATCCAGTCTATTAATTGTTGCCGGGAAGCT<br>AGAGTAAGTAGTTCGCCAGTTAATAGTTTGCGCAACGT<br>TGTTGCCATTGCTACAGGCATCGTGGTGTCACGCTCGT<br>CGTTTGGTATGGCTTCATTCAGCTCCGGTTCCCAACGA<br>TCAAGGCGAGTTACATGATCCCCCATGTTGTGCAAAA<br>AAGCGGTTAGCTCCTTCGGTCCTCCGATCGTTGTCAGA<br>AGTAAGTTGGCCGCAGTGTTATCACTCATGGTTATGGC<br>AGCACTGCATAATTCTCTTACTGTCATGCCATCCGTAA<br>GATGCTTTTCTGTGACTGGTGAGTACTCAACCAAGTCA<br>TTCTGAGAATAGTGTATGCGGCGACCGAGTTGCTCTTG<br>CCCGGCGTCAATACGGGATAATACCGCGCCACATAGC<br>AGAACTTTAAAAGTGCTCATCATTGGAAAACGTTCTTC<br>GGGGCGAAAACTCTCAAGGATCTTACCGCTGTTGAGA<br>TCCAGTTCGATGTAACCCACTCGTGCACCCAACTGATC<br>TTCAGCATCTTTTACTTTCACCAGCGTTTCTGGGTGAG<br>CAAAAACAGGAAGGCAAAATGCCGCAAAAAAGGGAA<br>TAAGGGCGACACGGAAATGTTGAATACTCATACTCTTC<br>CTTTTTCAATATTATTGAAGCATTTATCAGGGTTATTGT	

	CTCATGAGCGGATACATATTTGAATGTATTTAGAAAAATAAACAAAT	
**Plasmid sequence for PRINT template RNA IVT**		
Gf-full	ttgagatcctttttttctgcgcgtaatctgctgcttgcaaacaaaaaaaccaccgctaccagc<br>ggtggtttgtttgccggatcaagagctaccaactctttttccgaaggtaactggcttcagcag<br>agcgcagataccaaatactgttcttctagtgtagccgtagttaggccaccacttcaagaact<br>ctgtagcaccgcctacatacctcgctctgctaatcctgttaccagtggctgctgccagtggc<br>gataagtcgtgtcttaccgggttggactcaagacgatagttaccggataaggcgcagcggt<br>cgggctgaacggggggttcgtgcacacagcccagcttggagcgaacgacctacaccga<br>actgagatacctacagcgtgagctatgagaaagcgccacgcttcccgaagggagaaagg<br>cggacaggtatccggtaagcggcagggtcggaacaggagagcgcacgagggagcttc<br>cagggggaaacgcctggtatctttatagtcctgtcgggtttcgccacctctgacttgagcgt<br>cgatttttgtgatgctcgtcaggggggcggagcctatggaaaaacgccagcaacgcggc<br>ctttttacggttcctggccttttgctggccttttgctcacatgttctttccgatgaagactttttttt<br>tttttttttttgctattttccgaacacatataattaatatatgttccttttccgggttaagtaaaggt<br>ggcccgtccaccttggacaagtctgggagatcatgacaagtccggtaagggtatccgtgt<br>cagatggtattgacgcgttgtgaatcaaaaggcactgtccctactcactatgagcaccctac<br>ctatctttggcttgtggctgccttgcgaacctagctaccgtctgttttattgaaaatcaaataaa<br>agaaacccggtacatgagatcccccccactatacaaagattatctacctcagccacacaa<br>aaaccaacacacagatctaatgaacataaagatcttttattttacttgtacagctcgtccatgc<br>cgagagtgatcccggcggcggtcacgaactccagcaggaccatgtgatcgcgcttctcgt<br>tggggtctttgctcagggcggactgggtgctcaggtagtggttgtcgggcagcagcacgg<br>ggccgtcgccgatgggggtgttctgctggtagtggtcggcgagctgcacgctgccgtcct<br>cgatgttgtggcggatcttgaagttcaccttgatgccgttcttctgcttgtcggccatgatata<br>gacgttgtggctgttgtagttgtactccagcttgtgccccaggatgttgccgtcctccttgaa<br>gtcgatgcccttcagctcgatgcggttcaccagggtgtcgccctcgaacttcacctcggcg<br>cgggtcttgtagttgccgtcgtccttgaagaagatggtgcgctcctggacgtagccttcgg<br>gcatggcggacttgaagaagtcgtgctgcttcatgtggtcggggtagcggctgaagcact<br>gcacgccgtaggtcagggtggtcacgagggtgggccagggcacgggcagcttgccgg<br>tggtgcagatgaacttcagggtcagcttgccgtaggtggcatcgccctcgccctcgccgg<br>acacgctgaacttgtggccgtttacgtcgccgtccagctcgaccaggatgggcaccaccc<br>cggtgaacagctcctcgcccttgctcaccatggtggccgtacgacactcggagtgcggca<br>gcggcttttcttcccttgtgtaacagaacagacgcgggtggcggcggccgggagtttccg<br>agcgttccccggagcgagacaagcggttcactaaacgagctctgcttatatagacctccca<br>ccgtacacgcctaccgcccatttgcgtcaatggggcggagttgttacgacattttggaaagt<br>cccgttgattttggtgccaaaacaaactcccattgacgtcaatggggtggagacttggaaat<br>ccccgtgagtcaaacccctatccacgcccattgatgtactgccaaaaccgcatcaccatgg<br>taatagcgatgactaatacgtagatgtactgccaagtaggaaagtcccataaggtcatgtac<br>tgggcataatgccaggcgggccatttaccgtcattgacgtcaatagggggcgtacttggca<br>tatgatacacttgatgtactgccaagtgggcagtttaccgtaaatactccacccattgacgtc<br>aatggaaagtccctattggcgttactatgggaacatacgtcattattgacgtcaatgggcgg<br>gggtcgttgggcggtcagccaggcgggccatttaccgtaagttatgtaacgtctagagcc<br>agtcaaatttgccacagaaaaattaatccctgtgcccagcgggaccacgaggaggttctg<br>gctctatgactccttaagagagtcatagttactcccgccgtttaccctatagtgagtcgtatta<br>gaattccttaagatcattgggacgtcaggtggcacttttcggggaaatgtgcgcggaaccc<br>ctatttgtttatttttctaaatacattcaaatatgtatccgctcatgagacaataaccctgataaat<br>gcttcaataatattgaaaaaggaagagtatgagtattcaacatttccgtgtcgcccttattccc<br>ttttttgcggcattttgccttcctgtttttgctcacccagaaacgctggtgaaagtaaaagatgc<br>tgaagatcagttgggtgcacgagtgggttacatcgaactggatctcaacagcggtaagatc<br>cttgagagttttcgccccgaagaacgttttccaatgatgagcacttttaaagttctgctatgtg<br>gcgcggtattatcccgtattgacgccgggcaagagcaactcggtcgccgcatacactattc<br>tcagaatgacttggttgagtactcaccagtcacagaaaagcatcttacggatggcatgaca<br>gtaagagaattatgcagtgctgccataaccatgagtgataacactgcggccaacttacttct<br>gacaacgatcggaggaccgaaggagctaaccgcttttttgcacaacatgggggatcatgt	Fig. 1, Fig. 3, Fig. 4, Fig. S2, Fig. S8

	aactcgccttgatcgttgggaaccggagctgaatgaagccataccaaacgacgagcgtg acaccacgatgcctgtagcaatggcaacaacgttgcgcaaactattaactggcgaactact tactctagcttcccggcaacaattaatagactggatggaggcggataaagttgcaggacca cttctgcgctcggcccttccggctggctggtttattgctgataaatctggagccggtgagcg tggatctcgcggtatcattgcagcactggggccagatggtaagccctcccgtatcgtagtta tctacacgacggggagtcaggcaactatggatgaacgaaatagacagatcgctgagata ggtgcctcactgattaagcattggtaactgtcagaccaagtttactcatatatactttagattga tttaaaacttcatttttaatttaaaaggatctaggtgaagatcctttttgataatctcatgaccaaa atcccttaacgtgagttttcgttccactgagcgtcagaccccgtagaaaagatcaaaggatc ttc	
Pm-full	GAAGATCCTTTGATCTTTTCTACGGGGTCTGACGCTCA GTGGAACGAAAACTCACGTTAAGGGATTTTGGTCATG AGATTATCAAAAAGGATCTTCACCTAGATCCTTTTAAA TTAAAAATGAAGTTTTAAATCAATCTAAAGTATATATG AGTAAACTTGGTCTGACAGTTACCAATGCTTAATCAGT GAGGCACCTATCTCAGCGATCTGTCTATTTCGTTCATC CATAGTTGCCTGACTCCCCGTCGTGTAGATAACTACGA TACGGGAGGGCTTACCATCTGGCCCCAGTGCTGCAAT GATACCGCGAGATCCACGCTCACCGGCTCCAGATTTAT CAGCAATAAACCAGCCAGCCGGAAGGGCCGAGCGCA GAAGTGGTCCTGCAACTTTATCCGCCTCCATCCAGTCT ATTAATTGTTGCCGGGAAGCTAGAGTAAGTAGTTCGCC AGTTAATAGTTTGCGCAACGTTGTTGCCATTGCTACAG GCATCGTGGTGTCACGCTCGTCGTTTGGTATGGCTTCA TTCAGCTCCGGTTCCCAACGATCAAGGCGAGTTACATG ATCCCCCATGTTGTGCAAAAAAGCGGTTAGCTCCTTCG GTCCTCCGATCGTTGTCAGAAGTAAGTTGGCCGCAGTG TTATCACTCATGGTTATGGCAGCACTGCATAATTCTCT TACTGTCATGCCATCCGTAAGATGCTTTTCTGTGACTG GTGAGTACTCAACCAAGTCATTCTGAGAATAGTGTATG CGGCGACCGAGTTGCTCTTGCCCGGCGTCAATACGGG ATAATACCGCGCCACATAGCAGAACTTTAAAAGTGCT CATCATTGGAAAACGTTCTTCGGGGCGAAAACTCTCA AGGATCTTACCGCTGTTGAGATCCAGTTCGATGTAACC CACTCGTGCACCCAACTGATCTTCAGCATCTTTTACTTT CACCAGCGTTTCTGGGTGAGCAAAAACAGGAAGGCAA AATGCCGCAAAAAAGGGAATAAGGGCGACACGGAAA TGTTGAATACTCATACTCTTCCTTTTTCAATATTATTGA AGCATTTATCAGGGTTATTGTCTCATGAGCGGATACAT ATTTGAATGTATTTAGAAAAATAAACAAATAGGGGTT CCGCGCACATTTCCCCGAAAAGTGCCACCTGACGTCCC AATGATcttaaggaattctaatacgactcactatagggtaaacGGCGGGAGT AACTATGACTCTCTTAAGGAGTCATAGAGCCAGAACC TCCTCGTGGTCCCGCTGGGCACAGGGATTAATTTTTCT GTGGCAAATTTGACTGGCTCTAGACGTTACATAACTTA CGGTAAATGGCCCGCCTGGCTGACCGCCCAACGACCC CCGCCCATTGACGTCAATAATGACGTATGTTCCCATAG TAACGCCAATAGGGACTTTCCATTGACGTCAATGGGTG GAGTATTTACGGTAAACTGCCCACTTGGCAGTACATCA AGTGTATCATATGCCAAGTACGCCCCCTATTGACGTCA ATGACGGTAAATGGCCCGCCTGGCATTATGCCCAGTA CATGACCTTATGGGACTTTCCTACTTGGCAGTACATCT ACGTATTAGTCATCGCTATTACCATGGTGATGCGGTTT TGGCAGTACATCAATGGGCGTGGATAGGGGTTTGACT CACGGGGATTTCCAAGTCTCCACCCCATTGACGTCAAT GGGAGTTTGTTTTGGCACCAAAATCAACGGGACTTTCC	Fig. 1, Fig. S2

	AAAATGTCGTAACAACTCCGCCCCATTGACGCAAATG<br>GGCGGTAGGCGTGTACGGTGGGAGGTCTATATAAGCA<br>GAGCTCGTTTAGTGAACCGCTTGTCTCGCTCCGGGGAA<br>CGCTCGGAAACTCCCGGCCGCCGCCACCCGCGTCTGTT<br>CTGTTACACAAGGGAAGAAAAGCCGCTGCCGCACTCC<br>GAGTGTCGTACGGCCACCATGGTGAGCAAGGGCGAGG<br>AGCTGTTCACCGGGGTGGTGCCCATCCTGGTCGAGCTG<br>GACGGCGACGTAAACGGCCACAAGTTCAGCGTGTCCG<br>GCGAGGGCGAGGGCGATGCCACCTACGGCAAGCTGAC<br>CCTGAAGTTCATCTGCACCACCGGCAAGCTGCCCGTGC<br>CCTGGCCCACCCTCGTGACCACCCTGACCTACGGCGTG<br>CAGTGCTTCAGCCGCTACCCCGACCACATGAAGCAGC<br>ACGACTTCTTCAAGTCCGCCATGCCCGAAGGCTACGTC<br>CAGGAGCGCACCATCTTCTTCAAGGACGACGGCAACT<br>ACAAGACCCGCGCCGAGGTGAAGTTCGAGGGCGACAC<br>CCTGGTGAACCGCATCGAGCTGAAGGGCATCGACTTC<br>AAGGAGGACGGCAACATCCTGGGGCACAAGCTGGAGT<br>ACAACTACAACAGCCACAACGTCTATATCATGGCCGA<br>CAAGCAGAAGAACGGCATCAAGGTGAACTTCAAGATC<br>CGCCACAACATCGAGGACGGCAGCGTGCAGCTCGCCG<br>ACCACTACCAGCAGAACACCCCCATCGGCGACGGCCC<br>CGTGCTGCTGCCCGACAACCACTACCTGAGCACCCAGT<br>CCGCCCTGAGCAAAGACCCCAACGAGAAGCGCGATCA<br>CATGGTCCTGCTGGAGTTCGTGACCGCCGCCGGGATCA<br>CTCTCGGCATGGACGAGCTGTACAAGTAAaataaaagatcttta<br>tgttcattagatctgtgtgttggttttttgtgtgGCTAATGAGGTTCTATCCCT<br>CATGTGCAGAATTTCCTTTCTAAACCTATCTCTTATCCA<br>AACTATATACCCGCCCCCCTTTTTCATGGGAAACTCGT<br>AATGATTACAATAATTCATGACCGCTCACTGGACACG<br>GCAACCCTGGTTGGACGGGCCTCCAGGGGTGTACATA<br>CACTCCGAATAACTCGAAAAAGAAACCCGCGAGGGTT<br>TTCAAAGTAGCAAAAAAAAAAAAAAAAAAAAAAAAAAAG<br>TCTTCATCGGAAAGAACATGTGAGCAAAAGGCCAGCA<br>AAAGGCCAGGAACCGTAAAAAGGCCGCGTTGCTGGCG<br>TTTTTCCATAGGCTCCGCCCCCCTGACGAGCATCACAA<br>AAATCGACGCTCAAGTCAGAGGTGGCGAAACCCGACA<br>GGACTATAAAGATACCAGGCGTTTCCCCCTGGAAGCT<br>CCCTCGTGCGCTCTCCTGTTCCGACCCTGCCGCTTACC<br>GGATACCTGTCCGCCTTTCTCCCTTCGGGAAGCGTGGC<br>GCTTTCTCATAGCTCACGCTGTAGGTATCTCAGTTCGG<br>TGTAGGTCGTTCGCTCCAAGCTGGGCTGTGTGCACGAA<br>CCCCCCGTTCAGCCCGACCGCTGCGCCTTATCCGGTAA<br>CTATCGTCTTGAGTCCAACCCGGTAAGACACGACTTAT<br>CGCCACTGGCAGCAGCCACTGGTAACAGGATTAGCAG<br>AGCGAGGTATGTAGGCGGTGCTACAGAGTTCTTGAAG<br>TGGTGGCCTAACTACGGCTACACTAGAAGAACAGTAT<br>TTGGTATCTGCGCTCTGCTGAAGCCAGTTACCTTCGGA<br>AAAAGAGTTGGTAGCTCTTGATCCGGCAAACAAACCA<br>CCGCTGGTAGCGGTGGTTTTTTTGTTTGCAAGCAGCAG<br>ATTACGCGCAGAAAAAAAGGATCTCAA	
Gf-98	GTCCTCCGATCGTTGTCAGAAGTAAGTTGGCCGCAGTG<br>TTATCACTCATGGTTATGGCAGCACTGCATAATTCTCT<br>TACTGTCATGCCATCCGTAAGATGCTTTTCTGTGACTG<br>GTGAGTACTCAACCAAGTCATTCTGAGAATAGTGTATG<br>CGGCGACCGAGTTGCTCTTGCCCGGCGTCAATACGGG<br>ATAATACCGCGCCACATAGCAGAACTTTAAAAGTGCT	Fig. 1, Fig. 2, Fig. S2

	CATCATTGGAAAACGTTCTTCGGGGCGAAAACTCTCA<br>AGGATCTTACCGCTGTTGAGATCCAGTTCGATGTAACC<br>CACTCGTGCACCCAACTGATCTTCAGCATCTTTTACTTT<br>CACCAGCGTTTCTGGGTGAGCAAAAACAGGAAGGCAA<br>AATGCCGCAAAAAAGGGAATAAGGGCGACACGGAAA<br>TGTTGAATACTCATACTCTTCCTTTTTCAATATTATTGA<br>AGCATTTATCAGGGTTATTGTCTCATGAGCGGATACAT<br>ATTTGAATGTATTTAGAAAAATAAACAAATAGGGGTT<br>CCGCGCACATTTCCCCGAAAAGTGCCACCTGACGTCCC<br>AATGATcttaaggaattctaatacgactcactatagggtaaacGGCGGGAGT<br>AACTATGACTCTCTTAAGGAGTCATAGAGCCAGAACC<br>TCCTCGTGGTCCCGCTGGGCACAGGGATTAATTTTTCT<br>GTGGCAAATTTGACTGGCTCTAGACGTTACATAACTTA<br>CGGTAAATGGCCCGCCTGGCTGACCGCCCAACGACCC<br>CCGCCCATTGACGTCAATAATGACGTATGTTCCCATAG<br>TAACGCCAATAGGGACTTTCCATTGACGTCAATGGGTG<br>GAGTATTTACGGTAAACTGCCCACTTGGCAGTACATCA<br>AGTGTATCATATGCCAAGTACGCCCCCTATTGACGTCA<br>ATGACGGTAAATGGCCCGCCTGGCATTATGCCCAGTA<br>CATGACCTTATGGGACTTTCCTACTTGGCAGTACATCT<br>ACGTATTAGTCATCGCTATTACCATGGTGATGCGGTTT<br>TGGCAGTACATCAATGGGCGTGGATAGGGGTTTGACT<br>CACGGGGATTTCCAAGTCTCCACCCCATTGACGTCAAT<br>GGGAGTTTGTTTTGGCACCAAAATCAACGGGACTTTCC<br>AAAATGTCGTAACAACTCCGCCCCATTGACGCAAATG<br>GGCGGTAGGCGTGTACGGTGGGAGGTCTATATAAGCA<br>GAGCTCGTTTAGTGAACCGCTTGTCTCGCTCCGGGGAA<br>CGCTCGGAAACTCCCGGCCGCCGCCACCCGCGTCTGTT<br>CTGTTACACAAGGGAAGAAAAGCCGCTGCCGCACTCC<br>GAGTGTCGTACGGCCACCATGGTGAGCAAGGGCGAGG<br>AGCTGTTCACCGGGGTGGTGCCCATCCTGGTCGAGCTG<br>GACGGCGACGTAAACGGCCACAAGTTCAGCGTGTCCG<br>GCGAGGGCGAGGGCGATGCCACCTACGGCAAGCTGAC<br>CCTGAAGTTCATCTGCACCACCGGCAAGCTGCCCGTGC<br>CCTGGCCCACCCTCGTGACCACCCTGACCTACGGCGTG<br>CAGTGCTTCAGCCGCTACCCCGACCACATGAAGCAGC<br>ACGACTTCTTCAAGTCCGCCATGCCCGAAGGCTACGTC<br>CAGGAGCGCACCATCTTCTTCAAGGACGACGGCAACT<br>ACAAGACCCGCGCCGAGGTGAAGTTCGAGGGCGACAC<br>CCTGGTGAACCGCATCGAGCTGAAGGGCATCGACTTC<br>AAGGAGGACGGCAACATCCTGGGGCACAAGCTGGAGT<br>ACAACTACAACAGCCACAACGTCTATATCATGGCCGA<br>CAAGCAGAAGAACGGCATCAAGGTGAACTTCAAGATC<br>CGCCACAACATCGAGGACGGCAGCGTGCAGCTCGCCG<br>ACCACTACCAGCAGAACACCCCCATCGGCGACGGCCC<br>CGTGCTGCTGCCCGACAACCACTACCTGAGCACCCAGT<br>CCGCCCTGAGCAAAGACCCCAACGAGAAGCGCGATCA<br>CATGGTCCTGCTGGAGTTCGTGACCGCCGCCGGGATCA<br>CTCTCGGCATGGACGAGCTGTACAAGTAAaataaaagatcttta<br>tgttcattagatctgtgtgttggtttttgtgtgGCTGAccggacttgtcatgatctcccaga<br>cttgtccaaggtggacgggccacctttacttaacccggaaaaggaacatatattaattatatg<br>tgttcggaaaaTAGCAAAAAAAAAAAAAAAAAAAAAAAAAG<br>TCTTCATCGGAAAGAACATGTGAGCAAAAGGCCAGCA<br>AAAGGCCAGGAACCGTAAAAAGGCCGCGTTGCTGGCG<br>TTTTTCCATAGGCTCCGCCCCCCTGACGAGCATCACAA<br>AAATCGACGCTCAAGTCAGAGGTGGCGAAACCCGACA	

	GGACTATAAAGATACCAGGCGTTTCCCCCTGGAAGCT<br>CCCTCGTGCGCTCTCCTGTTCCGACCCTGCCGCTTACC<br>GGATACCTGTCCGCCTTTCTCCCTTCGGGAAGCGTGGC<br>GCTTTCTCATAGCTCACGCTGTAGGTATCTCAGTTCGG<br>TGTAGGTCGTTCGCTCCAAGCTGGGCTGTGTGCACGAA<br>CCCCCCGTTCAGCCCGACCGCTGCGCCTTATCCGGTAA<br>CTATCGTCTTGAGTCCAACCCGGTAAGACACGACTTAT<br>CGCCACTGGCAGCAGCCACTGGTAACAGGATTAGCAG<br>AGCGAGGTATGTAGGCGGTGCTACAGAGTTCTTGAAG<br>TGGTGGCCTAACTACGGCTACACTAGAAGAACAGTAT<br>TTGGTATCTGCGCTCTGCTGAAGCCAGTTACCTTCGGA<br>AAAAGAGTTGGTAGCTCTTGATCCGGCAAACAAACCA<br>CCGCTGGTAGCGGTGGTTTTTTTGTTTGCAAGCAGCAG<br>ATTACGCGCAGAAAAAAAGGATCTCAAGAAGATCCTT<br>TGATCTTTTCTACGGGGTCTGACGCTCAGTGGAACGAA<br>AACTCACGTTAAGGGATTTTGGTCATGAGATTATCAAA<br>AAGGATCTTCACCTAGATCCTTTTAAATTAAAAATGAA<br>GTTTTAAATCAATCTAAAGTATATATGAGTAAACTTGG<br>TCTGACAGTTACCAATGCTTAATCAGTGAGGCACCTAT<br>CTCAGCGATCTGTCTATTTCGTTCATCCATAGTTGCCTG<br>ACTCCCCGTCGTGTAGATAACTACGATACGGGAGGGC<br>TTACCATCTGGCCCCAGTGCTGCAATGATACCGCGAGA<br>CCCACGCTCACCGGCTCCAGATTTATCAGCAATAAACC<br>AGCCAGCCGGAAGGGCCGAGCGCAGAAGTGGTCCTGC<br>AACTTTATCCGCCTCCATCCAGTCTATTAATTGTTGCC<br>GGGAAGCTAGAGTAAGTAGTTCGCCAGTTAATAGTTT<br>GCGCAACGTTGTTGCCATTGCTACAGGCATCGTGGTGT<br>CACGCTCGTCGTTTGGTATGGCTTCATTCAGCTCCGGT<br>TCCCAACGATCAAGGCGAGTTACATGATCCCCCATGTT<br>GTGCAAAAAAGCGGTTAGCTCCTTCG	
Gf-68	GAAGATCCTTTGATCTTTTCTACGGGGTCTGACGCTCA<br>GTGGAACGAAAACTCACGTTAAGGGATTTTGGTCATG<br>AGATTATCAAAAAGGATCTTCACCTAGATCCTTTTAAA<br>TTAAAAATGAAGTTTTAAATCAATCTAAAGTATATATG<br>AGTAAACTTGGTCTGACAGTTACCAATGCTTAATCAGT<br>GAGGCACCTATCTCAGCGATCTGTCTATTTCGTTCATC<br>CATAGTTGCCTGACTCCCCGTCGTGTAGATAACTACGA<br>TACGGGAGGGCTTACCATCTGGCCCCAGTGCTGCAAT<br>GATACCGCGAGATCCACGCTCACCGGCTCCAGATTTAT<br>CAGCAATAAACCAGCCAGCCGGAAGGGCCGAGCGCA<br>GAAGTGGTCCTGCAACTTTATCCGCCTCCATCCAGTCT<br>ATTAATTGTTGCCGGGAAGCTAGAGTAAGTAGTTCGCC<br>AGTTAATAGTTTGCGCAACGTTGTTGCCATTGCTACAG<br>GCATCGTGGTGTCACGCTCGTCGTTTGGTATGGCTTCA<br>TTCAGCTCCGGTTCCCAACGATCAAGGCGAGTTACATG<br>ATCCCCCATGTTGTGCAAAAAAGCGGTTAGCTCCTTCG<br>GTCCTCCGATCGTTGTCAGAAGTAAGTTGGCCGCAGTG<br>TTATCACTCATGGTTATGGCAGCACTGCATAATTCTCT<br>TACTGTCATGCCATCCGTAAGATGCTTTTCTGTGACTG<br>GTGAGTACTCAACCAAGTCATTCTGAGAATAGTGTATG<br>CGGCGACCGAGTTGCTCTTGCCCGGCGTCAATACGGG<br>ATAATACCGCGCCACATAGCAGAACTTTAAAAGTGCT<br>CATCATTGGAAAACGTTCTTCGGGGCGAAAACTCTCA<br>AGGATCTTACCGCTGTTGAGATCCAGTTCGATGTAACC<br>CACTCGTGCACCCAACTGATCTTCAGCATCTTTTACTTT<br>CACCAGCGTTTCTGGGTGAGCAAAAACAGGAAGGCAA	Fig. 1, Fig. S2

	AATGCCGCAAAAAAGGGAATAAGGGCGACACGGAAA TGTTGAATACTCATACTCTTCCTTTTTCAATATTATTGA AGCATTTATCAGGGTTATTGTCTCATGAGCGGATACAT ATTTGAATGTATTTAGAAAAATAAACAAATAGGGGTT CCGCGCACATTTCCCCGAAAAGTGCCACCTGACGTCCC AATGATcttaaggaattctaatacgactcactatagggtaaacGGCGGGAGT AACTATGACTCTCTTAAGGAGTCATAGAGCCAGAACC TCCTCGTGGTCCCGCTGGGCACAGGGATTAATTTTTCT GTGGCAAATTTGACTGGCTCTAGACGTTACATAACTTA CGGTAAATGGCCCGCCTGGCTGACCGCCCAACGACCC CCGCCCATTGACGTCAATAATGACGTATGTTCCCATAG TAACGCCAATAGGGACTTTCCATTGACGTCAATGGGTG GAGTATTTACGGTAAACTGCCCACTTGGCAGTACATCA AGTGTATCATATGCCAAGTACGCCCCCTATTGACGTCA ATGACGGTAAATGGCCCGCCTGGCATTATGCCCAGTA CATGACCTTATGGGACTTTCCTACTTGGCAGTACATCT ACGTATTAGTCATCGCTATTACCATGGTGATGCGGTTT TGGCAGTACATCAATGGGCGTGGATAGGGGTTTGACT CACGGGGATTTCCAAGTCTCCACCCCATTGACGTCAAT GGGAGTTTGTTTTGGCACCAAAATCAACGGGACTTTCC AAAATGTCGTAACAACTCCGCCCCATTGACGCAAATG GGCGGTAGGCGTGTACGGTGGGAGGTCTATATAAGCA GAGCTCGTTTAGTGAACCGCTTGTCTCGCTCCGGGGAA CGCTCGGAAACTCCCGGCCGCCGCCACCCGCGTCTGTT CTGTTACACAAGGGAAGAAAAGCCGCTGCCGCACTCC GAGTGTCGTACGGCCACCATGGTGAGCAAGGGCGAGG AGCTGTTCACCGGGGTGGTGCCCATCCTGGTCGAGCTG GACGGCGACGTAAACGGCCACAAGTTCAGCGTGTCCG GCGAGGGCGAGGGCGATGCCACCTACGGCAAGCTGAC CCTGAAGTTCATCTGCACCACCGGCAAGCTGCCCGTGC CCTGGCCCACCCTCGTGACCACCCTGACCTACGGCGTG CAGTGCTTCAGCCGCTACCCCGACCACATGAAGCAGC ACGACTTCTTCAAGTCCGCCATGCCCGAAGGCTACGTC CAGGAGCGCACCATCTTCTTCAAGGACGACGGCAACT ACAAGACCCGCGCCGAGGTGAAGTTCGAGGGCGACAC CCTGGTGAACCGCATCGAGCTGAAGGGCATCGACTTC AAGGAGGACGGCAACATCCTGGGGCACAAGCTGGAGT ACAACTACAACAGCCACAACGTCTATATCATGGCCGA CAAGCAGAAGAACGGCATCAAGGTGAACTTCAAGATC CGCCACAACATCGAGGACGGCAGCGTGCAGCTCGCCG ACCACTACCAGCAGAACACCCCCATCGGCGACGGCCC CGTGCTGCTGCCCGACAACCACTACCTGAGCACCCAGT CCGCCCTGAGCAAAGACCCCAACGAGAAGCGCGATCA CATGGTCCTGCTGGAGTTCGTGACCGCCGCCGGGATCA CTCTCGGCATGGACGAGCTGTACAAGTAAaataaaagatcttta tgttcattagatctgtgtgttggttttttgtgtgGCTAACAAGGTGGACGGGC CACCTTTACTTAACCCGGAAAAGGAACATATATTAATT ATATGTGTTCGGAAAATAGCAAAAAAAAAAAAAAAA AAAAAAAAGTCTTCATCGGAAAGAACATGTGAGCAAA AGGCCAGCAAAAGGCCAGGAACCGTAAAAAGGCCGC GTTGCTGGCGTTTTTCCATAGGCTCCGCCCCCCTGACG AGCATCACAAAAATCGACGCTCAAGTCAGAGGTGGCG AAACCCGACAGGACTATAAAGATACCAGGCGTTTCCC CCTGGAAGCTCCCTCGTGCGCTCTCCTGTTCCGACCCT GCCGCTTACCGGATACCTGTCCGCCTTTCTCCCTTCGG GAAGCGTGGCGCTTTCTCATAGCTCACGCTGTAGGTAT	

	CTCAGTTCGGTGTAGGTCGTTCGCTCCAAGCTGGGCTG TGTGCACGAACCCCCCGTTCAGCCCGACCGCTGCGCCT TATCCGGTAACTATCGTCTTGAGTCCAACCCGGTAAGA CACGACTTATCGCCACTGGCAGCAGCCACTGGTAACA GGATTAGCAGAGCGAGGTATGTAGGCGGTGCTACAGA GTTCTTGAAGTGGTGGCCTAACTACGGCTACACTAGAA GAACAGTATTTGGTATCTGCGCTCTGCTGAAGCCAGTT ACCTTCGGAAAAAGAGTTGGTAGCTCTTGATCCGGCA AACAAACCACCGCTGGTAGCGGTGGTTTTTTTGTTTGC AAGCAGCAGATTACGCGCAGAAAAAAAGGATCTCAA	
Pm-112	GAAGATCCTTTGATCTTTTCTACGGGGTCTGACGCTCA GTGGAACGAAAACTCACGTTAAGGGATTTTGGTCATG AGATTATCAAAAAGGATCTTCACCTAGATCCTTTTAAA TTAAAAATGAAGTTTTAAATCAATCTAAAGTATATATG AGTAAACTTGGTCTGACAGTTACCAATGCTTAATCAGT GAGGCACCTATCTCAGCGATCTGTCTATTTCGTTCATC CATAGTTGCCTGACTCCCCGTCGTGTAGATAACTACGA TACGGGAGGGCTTACCATCTGGCCCCAGTGCTGCAAT GATACCGCGAGATCCACGCTCACCGGCTCCAGATTTAT CAGCAATAAACCAGCCAGCCGGAAGGGCCGAGCGCA GAAGTGGTCCTGCAACTTTATCCGCCTCCATCCAGTCT ATTAATTGTTGCCGGGAAGCTAGAGTAAGTAGTTCGCC AGTTAATAGTTTGCGCAACGTTGTTGCCATTGCTACAG GCATCGTGGTGTCACGCTCGTCGTTTGGTATGGCTTCA TTCAGCTCCGGTTCCCAACGATCAAGGCGAGTTACATG ATCCCCCATGTTGTGCAAAAAAGCGGTTAGCTCCTTCG GTCCTCCGATCGTTGTCAGAAGTAAGTTGGCCGCAGTG TTATCACTCATGGTTATGGCAGCACTGCATAATTCTCT TACTGTCATGCCATCCGTAAGATGCTTTTCTGTGACTG GTGAGTACTCAACCAAGTCATTCTGAGAATAGTGTATG CGGCGACCGAGTTGCTCTTGCCCGGCGTCAATACGGG ATAATACCGCGCCACATAGCAGAACTTTAAAAGTGCT CATCATTGGAAAACGTTCTTCGGGGCGAAAACTCTCA AGGATCTTACCGCTGTTGAGATCCAGTTCGATGTAACC CACTCGTGCACCCAACTGATCTTCAGCATCTTTTACTTT CACCAGCGTTTCTGGGTGAGCAAAAACAGGAAGGCAA AATGCCGCAAAAAAGGGAATAAGGGCGACACGGAAA TGTTGAATACTCATACTCTTCCTTTTTCAATATTATTGA AGCATTTATCAGGGTTATTGTCTCATGAGCGGATACAT ATTTGAATGTATTTAGAAAAATAAACAAATAGGGGTT CCGCGCACATTTCCCCGAAAAGTGCCACCTGACGTCCC AATGATcttaaggaattctaatacgactcactatagggtaaacGGCGGGAGT AACTATGACTCTCTTAAGGAGTCATAGAGCCAGAACC TCCTCGTGGTCCCGCTGGGCACAGGGATTAATTTTTCT GTGGCAAATTTGACTGGCTCTAGACGTTACATAACTTA CGGTAAATGGCCCGCCTGGCTGACCGCCCAACGACCC CCGCCCATTGACGTCAATAATGACGTATGTTCCCATAG TAACGCCAATAGGGACTTTCCATTGACGTCAATGGGTG GAGTATTTACGGTAAACTGCCCACTTGGCAGTACATCA AGTGTATCATATGCCAAGTACGCCCCCTATTGACGTCA ATGACGGTAAATGGCCCGCCTGGCATTATGCCCAGTA CATGACCTTATGGGACTTTCCTACTTGGCAGTACATCT ACGTATTAGTCATCGCTATTACCATGGTGATGCGGTTT TGGCAGTACATCAATGGGCGTGGATAGGGGTTTGACT CACGGGGATTTCCAAGTCTCCACCCCATTGACGTCAAT GGGAGTTTGTTTTGGCACCAAAATCAACGGGACTTTCC	Fig. 1, Fig. S2

	AAAATGTCGTAACAACTCCGCCCCATTGACGCAAATG<br>GGCGGTAGGCGTGTACGGTGGGAGGTCTATATAAGCA<br>GAGCTCGTTTAGTGAACCGCTTGTCTCGCTCCGGGGAA<br>CGCTCGGAAACTCCCGGCCGCCGCCACCCGCGTCTGTT<br>CTGTTACACAAGGGAAGAAAAGCCGCTGCCGCACTCC<br>GAGTGTCGTACGGCCACCATGGTGAGCAAGGGCGAGG<br>AGCTGTTCACCGGGGTGGTGCCCATCCTGGTCGAGCTG<br>GACGGCGACGTAAACGGCCACAAGTTCAGCGTGTCCG<br>GCGAGGGCGAGGGCGATGCCACCTACGGCAAGCTGAC<br>CCTGAAGTTCATCTGCACCACCGGCAAGCTGCCCGTGC<br>CCTGGCCCACCCTCGTGACCACCCTGACCTACGGCGTG<br>CAGTGCTTCAGCCGCTACCCCGACCACATGAAGCAGC<br>ACGACTTCTTCAAGTCCGCCATGCCCGAAGGCTACGTC<br>CAGGAGCGCACCATCTTCTTCAAGGACGACGGCAACT<br>ACAAGACCCGCGCCGAGGTGAAGTTCGAGGGCGACAC<br>CCTGGTGAACCGCATCGAGCTGAAGGGCATCGACTTC<br>AAGGAGGACGGCAACATCCTGGGGCACAAGCTGGAGT<br>ACAACTACAACAGCCACAACGTCTATATCATGGCCGA<br>CAAGCAGAAGAACGGCATCAAGGTGAACTTCAAGATC<br>CGCCACAACATCGAGGACGGCAGCGTGCAGCTCGCCG<br>ACCACTACCAGCAGAACACCCCCATCGGCGACGGCCC<br>CGTGCTGCTGCCCGACAACCACTACCTGAGCACCCAGT<br>CCGCCCTGAGCAAAGACCCCAACGAGAAGCGCGATCA<br>CATGGTCCTGCTGGAGTTCGTGACCGCCGCCGGGATCA<br>CTCTCGGCATGGACGAGCTGTACAAGTAAaataaaagatcttta<br>tgttcattagatctgtgtgttggttttttgtgtgGCTAATACAATAATTCATGA<br>CCGCTCACTGGACACGGCAACCCTGGTTGGACGGGCC<br>TCCAGGGGTGTACATACACTCCGAATAACTCGAAAAA<br>GAAACCCGCGAGGGTTTCAAAGTAGCAAAAAAAAAA<br>AAAAAAAAAAAAAAGTCTTCATCGGAAAGAACATGTG<br>AGCAAAAGGCCAGCAAAAGGCCAGGAACCGTAAAAA<br>GGCCGCGTTGCTGGCGTTTTTCCATAGGCTCCGCCCCC<br>CTGACGAGCATCACAAAAATCGACGCTCAAGTCAGAG<br>GTGGCGAAACCCGACAGGACTATAAAGATACCAGGCG<br>TTTCCCCCTGGAAGCTCCCTCGTGCGCTCTCCTGTTCCG<br>ACCCTGCCGCTTACCGGATACCTGTCCGCCTTTCTCCC<br>TTCGGGAAGCGTGGCGCTTTCTCATAGCTCACGCTGTA<br>GGTATCTCAGTTCGGTGTAGGTCGTTCGCTCCAAGCTG<br>GGCTGTGTGCACGAACCCCCCGTTCAGCCCGACCGCTG<br>CGCCTTATCCGGTAACTATCGTCTTGAGTCCAACCCGG<br>TAAGACACGACTTATCGCCACTGGCAGCAGCCACTGG<br>TAACAGGATTAGCAGAGCGAGGTATGTAGGCGGTGCT<br>ACAGAGTTCTTGAAGTGGTGGCCTAACTACGGCTACA<br>CTAGAAGAACAGTATTTGGTATCTGCGCTCTGCTGAAG<br>CCAGTTACCTTCGGAAAAAGAGTTGGTAGCTCTTGATC<br>CGGCAAACAAACCACCGCTGGTAGCGGTGGTTTTTTTG<br>TTTGCAAGCAGCAGATTACGCGCAGAAAAAAAGGATC<br>TCAA	
Gf-98 G-256>A	GTCCTCCGATCGTTGTCAGAAGTAAGTTGGCCGCAGTG<br>TTATCACTCATGGTTATGGCAGCACTGCATAATTCTCT<br>TACTGTCATGCCATCCGTAAGATGCTTTTCTGTGACTG<br>GTGAGTACTCAACCAAGTCATTCTGAGAATAGTGTATG<br>CGGCGACCGAGTTGCTCTTGCCCGGCGTCAATACGGG<br>ATAATACCGCGCCACATAGCAGAACTTTAAAAGTGCT<br>CATCATTGGAAAACGTTCTTCGGGGCGAAAACTCTCA<br>AGGATCTTACCGCTGTTGAGATCCAGTTCGATGTAACC	Fig. 2

	CACTCGTGCACCCAACTGATCTTCAGCATCTTTTACTTT CACCAGCGTTTCTGGGTGAGCAAAAACAGGAAGGCAA AATGCCGCAAAAAAGGGAATAAGGGCGACACGGAAA TGTTGAATACTCATACTCTTCCTTTTTCAATATTATTGA AGCATTTATCAGGGTTATTGTCTCATGAGCGGATACAT ATTTGAATGTATTTAGAAAAATAAACAAATAGGGGTT CCGCGCACATTTCCCCGAAAAGTGCCACCTGACGTCCC AATGATcttaaggaattctaatacgactcactatagggtaaacGGCGGGAGT AACTATGACTCTCTTAAGGAGTCATAGAGCCAGAACC TCCTCGTGGTCCCGCTGGGCACAGGGATTAATTTTTCT GTGGCAAATTTGACTGGCTCTAGACGTTACATAACTTA CGGTAAATGGCCCGCCTGGCTGACCGCCCAACGACCC CCGCCCATTGACGTCAATAATGACGTATGTTCCCATAG TAACGCCAATAGGGACTTTCCATTGACGTCAATGGGTG GAGTATTTACGGTAAACTGCCCACTTGGCAGTACATCA AGTGTATCATATGCCAAGTACGCCCCCTATTGACGTCA ATGACGGTAAATGGCCCGCCTGGCATTATGCCCAGTA CATGACCTTATGGGACTTTCCTACTTGGCAGTACATCT ACGTATTAGTCATCGCTATTACCATGGTGATGCGGTTT TGGCAGTACATCAATGGGCGTGGATAGGGGTTTGACT CACGGGGATTTCCAAGTCTCCACCCCATTGACGTCAAT GGGAGTTTGTTTTGGCACCAAAATCAACGGGACTTTCC AAAATGTCGTAACAACTCCGCCCCATTGACGCAAATG GGCGGTAGGCGTGTACGGTGGGAGGTCTATATAAGCA GAGCTCGTTTAGTGAACCGCTTGTCTCGCTCCGGGGAA CGCTCGGAAACTCCCGGCCGCCGCCACCCGCGTCTGTT CTGTTACACAAGGGAAGAAAAGCCGCTGCCGCACTCC GAGTGTCGTACGGCCACCATGGTGAGCAAGGGCGAGG AGCTGTTCACCGGGGTGGTGCCCATCCTGGTCGAGCTG GACGGCGACGTAAACGGCCACAAGTTCAGCGTGTCCG GCGAGGGCGAGGGCGATGCCACCTACGGCAAGCTGAC CCTGAAGTTCATCTGCACCACCGGCAAGCTGCCCGTGC CCTGGCCCACCCTCGTGACCACCCTGACCTACGGCGTG CAGTGCTTCAGCCGCTACCCCGACCACATGAAGCAGC ACGACTTCTTCAAGTCCGCCATGCCCGAAGGCTACGTC CAGGAGCGCACCATCTTCTTCAAGGACGACGGCAACT ACAAGACCCGCGCCGAGGTGAAGTTCGAGGGCGACAC CCTGGTGAACCGCATCGAGCTGAAGGGCATCGACTTC AAGGAGGACGGCAACATCCTGGGGCACAAGCTGGAGT ACAACTACAACAGCCACAACGTCTATATCATGGCCGA CAAGCAGAAGAACGGCATCAAGGTGAACTTCAAGATC CGCCACAACATCGAGGACGGCAGCGTGCAGCTCGCCG ACCACTACCAGCAGAACACCCCCATCGGCGACGGCCC CGTGCTGCTGCCCGACAACCACTACCTGAGCACCCAGT CCGCCCTGAGCAAAGACCCCAACGAGAAGCGCGATCA CATGGTCCTGCTGGAGTTCGTGACCGCCGCCGGGATCA CTCTCGGCATGGACGAGCTGTACAAGTAAaataaaagatcttta tgttcattagatctgtgtgttggttttttgtgtgGCTGACCGGACTTGTCATGA TCTCCCAGACTTGTCCAAGGTGGACGGGCCACCTTTAC TTAACCCGCAAAAGGAACATATATTAATTATATGTGTT CGGAAAATAGCAAAAAAAAAAAAAAAAAAAAAAAAAG TCTTCATCGGAAAGAACATGTGAGCAAAAGGCCAGCA AAAGGCCAGGAACCGTAAAAAGGCCGCGTTGCTGGCG TTTTTCCATAGGCTCCGCCCCCCTGACGAGCATCACAA AAATCGACGCTCAAGTCAGAGGTGGCGAAACCCGACA GGACTATAAAGATACCAGGCGTTTCCCCCTGGAAGCT	

	CCCTCGTGCGCTCTCCTGTTCCGACCCTGCCGCTTACC<br>GGATACCTGTCCGCCTTTCTCCCTTCGGGAAGCGTGGC<br>GCTTTCTCATAGCTCACGCTGTAGGTATCTCAGTTCGG<br>TGTAGGTCGTTCGCTCCAAGCTGGGCTGTGTGCACGAA<br>CCCCCCGTTCAGCCCGACCGCTGCGCCTTATCCGGTAA<br>CTATCGTCTTGAGTCCAACCCGGTAAGACACGACTTAT<br>CGCCACTGGCAGCAGCCACTGGTAACAGGATTAGCAG<br>AGCGAGGTATGTAGGCGGTGCTACAGAGTTCTTGAAG<br>TGGTGGCCTAACTACGGCTACACTAGAAGAACAGTAT<br>TTGGTATCTGCGCTCTGCTGAAGCCAGTTACCTTCGGA<br>AAAAGAGTTGGTAGCTCTTGATCCGGCAAACAAACCA<br>CCGCTGGTAGCGGTGGTTTTTTTGTTTGCAAGCAGCAG<br>ATTACGCGCAGAAAAAAAGGATCTCAAGAAGATCCTT<br>TGATCTTTTCTACGGGGTCTGACGCTCAGTGGAACGAA<br>AACTCACGTTAAGGGATTTTGGTCATGAGATTATCAAA<br>AAGGATCTTCACCTAGATCCTTTTAAATTAAAAATGAA<br>GTTTTAAATCAATCTAAAGTATATATGAGTAAACTTGG<br>TCTGACAGTTACCAATGCTTAATCAGTGAGGCACCTAT<br>CTCAGCGATCTGTCTATTTCGTTCATCCATAGTTGCCTG<br>ACTCCCCGTCGTGTAGATAACTACGATACGGGAGGGC<br>TTACCATCTGGCCCCAGTGCTGCAATGATACCGCGAGA<br>CCCACGCTCACCGGCTCCAGATTTATCAGCAATAAACC<br>AGCCAGCCGGAAGGGCCGAGCGCAGAAGTGGTCCTGC<br>AACTTTATCCGCCTCCATCCAGTCTATTAATTGTTGCC<br>GGGAAGCTAGAGTAAGTAGTTCGCCAGTTAATAGTTT<br>GCGCAACGTTGTTGCCATTGCTACAGGCATCGTGGTGT<br>CACGCTCGTCGTTTGGTATGGCTTCATTCAGCTCCGGT<br>TCCCAACGATCAAGGCGAGTTACATGATCCCCCATGTT<br>GTGCAAAAAAGCGGTTAGCTCCTTCG	
Gf-98 G-255>A	GTCCTCCGATCGTTGTCAGAAGTAAGTTGGCCGCAGTG<br>TTATCACTCATGGTTATGGCAGCACTGCATAATTCTCT<br>TACTGTCATGCCATCCGTAAGATGCTTTTCTGTGACTG<br>GTGAGTACTCAACCAAGTCATTCTGAGAATAGTGTATG<br>CGGCGACCGAGTTGCTCTTGCCCGGCGTCAATACGGG<br>ATAATACCGCGCCACATAGCAGAACTTTAAAAGTGCT<br>CATCATTGGAAAACGTTCTTCGGGGCGAAAACTCTCA<br>AGGATCTTACCGCTGTTGAGATCCAGTTCGATGTAACC<br>CACTCGTGCACCCAACTGATCTTCAGCATCTTTTACTTT<br>CACCAGCGTTTCTGGGTGAGCAAAAACAGGAAGGCAA<br>AATGCCGCAAAAAAGGGAATAAGGGCGACACGGAAA<br>TGTTGAATACTCATACTCTTCCTTTTTCAATATTATTGA<br>AGCATTTATCAGGGTTATTGTCTCATGAGCGGATACAT<br>ATTTGAATGTATTTAGAAAAATAAACAAATAGGGGTT<br>CCGCGCACATTTCCCCGAAAAGTGCCACCTGACGTCCC<br>AATGATcttaaggaattctaatacgactcactatagggtaaacGGCGGGAGT<br>AACTATGACTCTCTTAAGGAGTCATAGAGCCAGAACC<br>TCCTCGTGGTCCCGCTGGGCACAGGGATTAATTTTTCT<br>GTGGCAAATTTGACTGGCTCTAGACGTTACATAACTTA<br>CGGTAAATGGCCCGCCTGGCTGACCGCCCAACGACCC<br>CCGCCCATTGACGTCAATAATGACGTATGTTCCCATAG<br>TAACGCCAATAGGGACTTTCCATTGACGTCAATGGGTG<br>GAGTATTTACGGTAAACTGCCCACTTGGCAGTACATCA<br>AGTGTATCATATGCCAAGTACGCCCCCTATTGACGTCA<br>ATGACGGTAAATGGCCCGCCTGGCATTATGCCCAGTA<br>CATGACCTTATGGGACTTTCCTACTTGGCAGTACATCT<br>ACGTATTAGTCATCGCTATTACCATGGTGATGCGGTTT	Fig. 2

	TGGCAGTACATCAATGGGCGTGGATAGGGGTTTGACT CACGGGGATTTCCAAGTCTCCACCCCATTGACGTCAAT GGGAGTTTGTTTTGGCACCAAAATCAACGGGACTTTCC AAAATGTCGTAACAACTCCGCCCCATTGACGCAAATG GGCGGTAGGCGTGTACGGTGGGAGGTCTATATAAGCA GAGCTCGTTTAGTGAACCGCTTGTCTCGCTCCGGGGAA CGCTCGGAAACTCCCGGCCGCCGCCACCCGCGTCTGTT CTGTTACACAAGGGAAGAAAAGCCGCTGCCGCACTCC GAGTGTCGTACGGCCACCATGGTGAGCAAGGGCGAGG AGCTGTTCACCGGGGTGGTGCCCATCCTGGTCGAGCTG GACGGCGACGTAAACGGCCACAAGTTCAGCGTGTCCG GCGAGGGCGAGGGCGATGCCACCTACGGCAAGCTGAC CCTGAAGTTCATCTGCACCACCGGCAAGCTGCCCGTGC CCTGGCCCACCCTCGTGACCACCCTGACCTACGGCGTG CAGTGCTTCAGCCGCTACCCCGACCACATGAAGCAGC ACGACTTCTTCAAGTCCGCCATGCCCGAAGGCTACGTC CAGGAGCGCACCATCTTCTTCAAGGACGACGGCAACT ACAAGACCCGCGCCGAGGTGAAGTTCGAGGGCGACAC CCTGGTGAACCGCATCGAGCTGAAGGGCATCGACTTC AAGGAGGACGGCAACATCCTGGGGCACAAGCTGGAGT ACAACTACAACAGCCACAACGTCTATATCATGGCCGA CAAGCAGAAGAACGGCATCAAGGTGAACTTCAAGATC CGCCACAACATCGAGGACGGCAGCGTGCAGCTCGCCG ACCACTACCAGCAGAACACCCCCATCGGCGACGGCCC CGTGCTGCTGCCCGACAACCACTACCTGAGCACCCAGT CCGCCCTGAGCAAAGACCCCAACGAGAAGCGCGATCA CATGGTCCTGCTGGAGTTCGTGACCGCCGCCGGGATCA CTCTCGGCATGGACGAGCTGTACAAGTAAaataaaagatcttta tgttcattagatctgtgtgttggttttttgtgtgGCTGACCGGACTTGTCATGA TCTCCCAGACTTGTCCAAGGTGGACGGGCCACCTTTAC TTAACCCAGAAAAGGAACATATATTAATTATATGTGTT CGGAAAATAGCAAAAAAAAAAAAAAAAAAAAAAAAAG TCTTCATCGGAAAGAACATGTGAGCAAAAGGCCAGCA AAAGGCCAGGAACCGTAAAAAGGCCGCGTTGCTGGCG TTTTTCCATAGGCTCCGCCCCCCTGACGAGCATCACAA AAATCGACGCTCAAGTCAGAGGTGGCGAAACCCGACA GGACTATAAAGATACCAGGCGTTTCCCCCTGGAAGCT CCCTCGTGCGCTCTCCTGTTCCGACCCTGCCGCTTACC GGATACCTGTCCGCCTTTCTCCCTTCGGGAAGCGTGGC GCTTTCTCATAGCTCACGCTGTAGGTATCTCAGTTCGG TGTAGGTCGTTCGCTCCAAGCTGGGCTGTGTGCACGAA CCCCCCGTTCAGCCCGACCGCTGCGCCTTATCCGGTAA CTATCGTCTTGAGTCCAACCCGGTAAGACACGACTTAT CGCCACTGGCAGCAGCCACTGGTAACAGGATTAGCAG AGCGAGGTATGTAGGCGGTGCTACAGAGTTCTTGAAG TGGTGGCCTAACTACGGCTACACTAGAAGAACAGTAT TTGGTATCTGCGCTCTGCTGAAGCCAGTTACCTTCGGA AAAAGAGTTGGTAGCTCTTGATCCGGCAAACAAACCA CCGCTGGTAGCGGTGGTTTTTTTGTTTGCAAGCAGCAG ATTACGCGCAGAAAAAAAGGATCTCAAGAAGATCCTT TGATCTTTTCTACGGGGTCTGACGCTCAGTGGAACGAA AACTCACGTTAAGGGATTTTGGTCATGAGATTATCAAA AAGGATCTTCACCTAGATCCTTTTAAATTAAAAATGAA GTTTTAAATCAATCTAAAGTATATATGAGTAAACTTGG TCTGACAGTTACCAATGCTTAATCAGTGAGGCACCTAT CTCAGCGATCTGTCTATTTCGTTCATCCATAGTTGCCTG	

	ACTCCCCGTCGTGTAGATAACTACGATACGGGAGGGC<br>TTACCATCTGGCCCCAGTGCTGCAATGATACCGCGAGA<br>CCCACGCTCACCGGCTCCAGATTTATCAGCAATAAACC<br>AGCCAGCCGGAAGGGCCGAGCGCAGAAGTGGTCCTGC<br>AACTTTATCCGCCTCCATCCAGTCTATTAATTGTTGCC<br>GGGAAGCTAGAGTAAGTAGTTCGCCAGTTAATAGTTT<br>GCGCAACGTTGTTGCCATTGCTACAGGCATCGTGGTGT<br>CACGCTCGTCGTTTGGTATGGCTTCATTCAGCTCCGGT<br>TCCCAACGATCAAGGCGAGTTACATGATCCCCCATGTT<br>GTGCAAAAAAGCGGTTAGCTCCTTCG	
Gf-98 C-254>A	GTCCTCCGATCGTTGTCAGAAGTAAGTTGGCCGCAGTG<br>TTATCACTCATGGTTATGGCAGCACTGCATAATTCTCT<br>TACTGTCATGCCATCCGTAAGATGCTTTTCTGTGACTG<br>GTGAGTACTCAACCAAGTCATTCTGAGAATAGTGTATG<br>CGGCGACCGAGTTGCTCTTGCCCGGCGTCAATACGGG<br>ATAATACCGCGCCACATAGCAGAACTTTAAAAGTGCT<br>CATCATTGGAAAACGTTCTTCGGGGCGAAAACTCTCA<br>AGGATCTTACCGCTGTTGAGATCCAGTTCGATGTAACC<br>CACTCGTGCACCCAACTGATCTTCAGCATCTTTTACTTT<br>CACCAGCGTTTCTGGGTGAGCAAAAACAGGAAGGCAA<br>AATGCCGCAAAAAAGGGAATAAGGGCGACACGGAAA<br>TGTTGAATACTCATACTCTTCCTTTTTCAATATTATTGA<br>AGCATTTATCAGGGTTATTGTCTCATGAGCGGATACAT<br>ATTTGAATGTATTTAGAAAAATAAACAAATAGGGGTT<br>CCGCGCACATTTCCCCGAAAAGTGCCACCTGACGTCCC<br>AATGATcttaaggaattctaatacgactcactatagggtaaacGGCGGGAGT<br>AACTATGACTCTCTTAAGGAGTCATAGAGCCAGAACC<br>TCCTCGTGGTCCCGCTGGGCACAGGGATTAATTTTTCT<br>GTGGCAAATTTGACTGGCTCTAGACGTTACATAACTTA<br>CGGTAAATGGCCCGCCTGGCTGACCGCCCAACGACCC<br>CCGCCCATTGACGTCAATAATGACGTATGTTCCCATAG<br>TAACGCCAATAGGGACTTTCCATTGACGTCAATGGGTG<br>GAGTATTTACGGTAAACTGCCCACTTGGCAGTACATCA<br>AGTGTATCATATGCCAAGTACGCCCCCTATTGACGTCA<br>ATGACGGTAAATGGCCCGCCTGGCATTATGCCCAGTA<br>CATGACCTTATGGGACTTTCCTACTTGGCAGTACATCT<br>ACGTATTAGTCATCGCTATTACCATGGTGATGCGGTTT<br>TGGCAGTACATCAATGGGCGTGGATAGGGGTTTGACT<br>CACGGGGATTTCCAAGTCTCCACCCCATTGACGTCAAT<br>GGGAGTTTGTTTTGGCACCAAAATCAACGGGACTTTCC<br>AAAATGTCGTAACAACTCCGCCCCATTGACGCAAATG<br>GGCGGTAGGCGTGTACGGTGGGAGGTCTATATAAGCA<br>GAGCTCGTTTAGTGAACCGCTTGTCTCGCTCCGGGGAA<br>CGCTCGGAAACTCCCGGCCGCCGCCACCCGCGTCTGTT<br>CTGTTACACAAGGGAAGAAAAGCCGCTGCCGCACTCC<br>GAGTGTCGTACGGCCACCATGGTGAGCAAGGGCGAGG<br>AGCTGTTCACCGGGGTGGTGCCCATCCTGGTCGAGCTG<br>GACGGCGACGTAAACGGCCACAAGTTCAGCGTGTCCG<br>GCGAGGGCGAGGGCGATGCCACCTACGGCAAGCTGAC<br>CCTGAAGTTCATCTGCACCACCGGCAAGCTGCCCGTGC<br>CCTGGCCCACCCTCGTGACCACCCTGACCTACGGCGTG<br>CAGTGCTTCAGCCGCTACCCCGACCACATGAAGCAGC<br>ACGACTTCTTCAAGTCCGCCATGCCCGAAGGCTACGTC<br>CAGGAGCGCACCATCTTCTTCAAGGACGACGGCAACT<br>ACAAGACCCGCGCCGAGGTGAAGTTCGAGGGCGACAC<br>CCTGGTGAACCGCATCGAGCTGAAGGGCATCGACTTC	Fig. 2

	AAGGAGGACGGCAACATCCTGGGGCACAAGCTGGAGT<br>ACAACTACAACAGCCACAACGTCTATATCATGGCCGA<br>CAAGCAGAAGAACGGCATCAAGGTGAACTTCAAGATC<br>CGCCACAACATCGAGGACGGCAGCGTGCAGCTCGCCG<br>ACCACTACCAGCAGAACACCCCCATCGGCGACGGCCC<br>CGTGCTGCTGCCCGACAACCACTACCTGAGCACCCAGT<br>CCGCCCTGAGCAAAGACCCCAACGAGAAGCGCGATCA<br>CATGGTCCTGCTGGAGTTCGTGACCGCCGCCGGGATCA<br>CTCTCGGCATGGACGAGCTGTACAAGTAAaataaaagatcttta<br>tgttcattagatctgtgtgttggttttttgtgtgGCTGAccggacttgtcatgatctcccaga<br>cttgtccaaggtggacgggccacctttacttaaccaggaaaaggaacatatattaattatatg<br>tgttcggaaaaTAGCAAAAAAAAAAAAAAAAAAAAAAAAG<br>TCTTCATCGGAAAGAACATGTGAGCAAAAGGCCAGCA<br>AAAGGCCAGGAACCGTAAAAAGGCCGCGTTGCTGGCG<br>TTTTTCCATAGGCTCCGCCCCCCTGACGAGCATCACAA<br>AAATCGACGCTCAAGTCAGAGGTGGCGAAACCCGACA<br>GGACTATAAAGATACCAGGCGTTTCCCCCTGGAAGCT<br>CCCTCGTGCGCTCTCCTGTTCCGACCCTGCCGCTTACC<br>GGATACCTGTCCGCCTTTCTCCCTTCGGGAAGCGTGGC<br>GCTTTCTCATAGCTCACGCTGTAGGTATCTCAGTTCGG<br>TGTAGGTCGTTCGCTCCAAGCTGGGCTGTGTGCACGAA<br>CCCCCCGTTCAGCCCGACCGCTGCGCCTTATCCGGTAA<br>CTATCGTCTTGAGTCCAACCCGGTAAGACACGACTTAT<br>CGCCACTGGCAGCAGCCACTGGTAACAGGATTAGCAG<br>AGCGAGGTATGTAGGCGGTGCTACAGAGTTCTTGAAG<br>TGGTGGCCTAACTACGGCTACACTAGAAGAACAGTAT<br>TTGGTATCTGCGCTCTGCTGAAGCCAGTTACCTTCGGA<br>AAAAGAGTTGGTAGCTCTTGATCCGGCAAACAAACCA<br>CCGCTGGTAGCGGTGGTTTTTTTGTTTGCAAGCAGCAG<br>ATTACGCGCAGAAAAAAAGGATCTCAAGAAGATCCTT<br>TGATCTTTTCTACGGGGTCTGACGCTCAGTGGAACGAA<br>AACTCACGTTAAGGGATTTTGGTCATGAGATTATCAAA<br>AAGGATCTTCACCTAGATCCTTTTAAATTAAAAATGAA<br>GTTTTAAATCAATCTAAAGTATATATGAGTAAACTTGG<br>TCTGACAGTTACCAATGCTTAATCAGTGAGGCACCTAT<br>CTCAGCGATCTGTCTATTTCGTTCATCCATAGTTGCCTG<br>ACTCCCCGTCGTGTAGATAACTACGATACGGGAGGGC<br>TTACCATCTGGCCCCAGTGCTGCAATGATACCGCGAGA<br>CCCACGCTCACCGGCTCCAGATTTATCAGCAATAAACC<br>AGCCAGCCGGAAGGGCCGAGCGCAGAAGTGGTCCTGC<br>AACTTTATCCGCCTCCATCCAGTCTATTAATTGTTGCC<br>GGGAAGCTAGAGTAAGTAGTTCGCCAGTTAATAGTTT<br>GCGCAACGTTGTTGCCATTGCTACAGGCATCGTGGTGT<br>CACGCTCGTCGTTTGGTATGGCTTCATTCAGCTCCGGT<br>TCCCAACGATCAAGGCGAGTTACATGATCCCCCATGTT<br>GTGCAAAAAAGCGGTTAGCTCCTTCG	
Gf-98 G-235>U C-254>A	GTCCTCCGATCGTTGTCAGAAGTAAGTTGGCCGCAGTG<br>TTATCACTCATGGTTATGGCAGCACTGCATAATTCTCT<br>TACTGTCATGCCATCCGTAAGATGCTTTTCTGTGACTG<br>GTGAGTACTCAACCAAGTCATTCTGAGAATAGTGTATG<br>CGGCGACCGAGTTGCTCTTGCCCGGCGTCAATACGGG<br>ATAATACCGCGCCACATAGCAGAACTTTAAAAGTGCT<br>CATCATTGGAAAACGTTCTTCGGGGCGAAAACTCTCA<br>AGGATCTTACCGCTGTTGAGATCCAGTTCGATGTAACC<br>CACTCGTGCACCCAACTGATCTTCAGCATCTTTTACTTT<br>CACCAGCGTTTCTGGGTGAGCAAAAACAGGAAGGCAA	Fig. 2

	AATGCCGCAAAAAAGGGAATAAGGGCGACACGGAAA<br>TGTTGAATACTCATACTCTTCCTTTTTCAATATTATTGA<br>AGCATTTATCAGGGTTATTGTCTCATGAGCGGATACAT<br>ATTTGAATGTATTTAGAAAAATAAACAAATAGGGGTT<br>CCGCGCACATTTCCCCGAAAAGTGCCACCTGACGTCCC<br>AATGATcttaaggaattctaatacgactcactatagggtaaacGGCGGGAGT<br>AACTATGACTCTCTTAAGGAGTCATAGAGCCAGAACC<br>TCCTCGTGGTCCCGCTGGGCACAGGGATTAATTTTTCT<br>GTGGCAAATTTGACTGGCTCTAGACGTTACATAACTTA<br>CGGTAAATGGCCCGCCTGGCTGACCGCCCAACGACCC<br>CCGCCCATTGACGTCAATAATGACGTATGTTCCCATAG<br>TAACGCCAATAGGGACTTTCCATTGACGTCAATGGGTG<br>GAGTATTTACGGTAAACTGCCCACTTGGCAGTACATCA<br>AGTGTATCATATGCCAAGTACGCCCCCTATTGACGTCA<br>ATGACGGTAAATGGCCCGCCTGGCATTATGCCCAGTA<br>CATGACCTTATGGGACTTTCCTACTTGGCAGTACATCT<br>ACGTATTAGTCATCGCTATTACCATGGTGATGCGGTTT<br>TGGCAGTACATCAATGGGCGTGGATAGGGGTTTGACT<br>CACGGGGATTTCCAAGTCTCCACCCCATTGACGTCAAT<br>GGGAGTTTGTTTTGGCACCAAAATCAACGGGACTTTCC<br>AAAATGTCGTAACAACTCCGCCCCATTGACGCAAATG<br>GGCGGTAGGCGTGTACGGTGGGAGGTCTATATAAGCA<br>GAGCTCGTTTAGTGAACCGCTTGTCTCGCTCCGGGGAA<br>CGCTCGGAAACTCCCGGCCGCCGCCACCCGCGTCTGTT<br>CTGTTACACAAGGGAAGAAAAGCCGCTGCCGCACTCC<br>GAGTGTCGTACGGCCACCATGGTGAGCAAGGGCGAGG<br>AGCTGTTCACCGGGGTGGTGCCCATCCTGGTCGAGCTG<br>GACGGCGACGTAAACGGCCACAAGTTCAGCGTGTCCG<br>GCGAGGGCGAGGGCGATGCCACCTACGGCAAGCTGAC<br>CCTGAAGTTCATCTGCACCACCGGCAAGCTGCCCGTGC<br>CCTGGCCCACCCTCGTGACCACCCTGACCTACGGCGTG<br>CAGTGCTTCAGCCGCTACCCCGACCACATGAAGCAGC<br>ACGACTTCTTCAAGTCCGCCATGCCCGAAGGCTACGTC<br>CAGGAGCGCACCATCTTCTTCAAGGACGACGGCAACT<br>ACAAGACCCGCGCCGAGGTGAAGTTCGAGGGCGACAC<br>CCTGGTGAACCGCATCGAGCTGAAGGGCATCGACTTC<br>AAGGAGGACGGCAACATCCTGGGGCACAAGCTGGAGT<br>ACAACTACAACAGCCACAACGTCTATATCATGGCCGA<br>CAAGCAGAAGAACGGCATCAAGGTGAACTTCAAGATC<br>CGCCACAACATCGAGGACGGCAGCGTGCAGCTCGCCG<br>ACCACTACCAGCAGAACACCCCCATCGGCGACGGCCC<br>CGTGCTGCTGCCCGACAACCACTACCTGAGCACCCAGT<br>CCGCCCTGAGCAAAGACCCCAACGAGAAGCGCGATCA<br>CATGGTCCTGCTGGAGTTCGTGACCGCCGCCGGGATCA<br>CTCTCGGCATGGACGAGCTGTACAAGTAAaataaaagatcttta<br>tgttcattagatctgtgtgttggttttttgtgtgGCTGAccggacttgtcatgatctcccaga<br>cttgtccaaggtggacTggccacctttacttaaccaggaaaaggaacatatattaattatat<br>gtgttcggaaaaTAGCAAAAAAAAAAAAAAAAAAAAAAAAAG<br>TCTTCATCGGAAAGAACATGTGAGCAAAAGGCCAGCA<br>AAAGGCCAGGAACCGTAAAAAGGCCGCGTTGCTGGCG<br>TTTTTCCATAGGCTCCGCCCCCCTGACGAGCATCACAA<br>AAATCGACGCTCAAGTCAGAGGTGGCGAAACCCGACA<br>GGACTATAAAGATACCAGGCGTTTCCCCCTGGAAGCT<br>CCCTCGTGCGCTCTCCTGTTCCGACCCTGCCGCTTACC<br>GGATACCTGTCCGCCTTTCTCCCTTCGGGAAGCGTGGC<br>GCTTTCTCATAGCTCACGCTGTAGGTATCTCAGTTCGG	

	TGTAGGTCGTTCGCTCCAAGCTGGGCTGTGTGCACGAA<br>CCCCCCGTTCAGCCCGACCGCTGCGCCTTATCCGGTAA<br>CTATCGTCTTGAGTCCAACCCGGTAAGACACGACTTAT<br>CGCCACTGGCAGCAGCCACTGGTAACAGGATTAGCAG<br>AGCGAGGTATGTAGGCGGTGCTACAGAGTTCTTGAAG<br>TGGTGGCCTAACTACGGCTACACTAGAAGAACAGTAT<br>TTGGTATCTGCGCTCTGCTGAAGCCAGTTACCTTCGGA<br>AAAAGAGTTGGTAGCTCTTGATCCGGCAAACAAACCA<br>CCGCTGGTAGCGGTGGTTTTTTTGTTTGCAAGCAGCAG<br>ATTACGCGCAGAAAAAAAGGATCTCAAGAAGATCCTT<br>TGATCTTTTCTACGGGGTCTGACGCTCAGTGGAACGAA<br>AACTCACGTTAAGGGATTTTGGTCATGAGATTATCAAA<br>AAGGATCTTCACCTAGATCCTTTTAAATTAAAAATGAA<br>GTTTTAAATCAATCTAAAGTATATATGAGTAAACTTGG<br>TCTGACAGTTACCAATGCTTAATCAGTGAGGCACCTAT<br>CTCAGCGATCTGTCTATTTCGTTCATCCATAGTTGCCTG<br>ACTCCCCGTCGTGTAGATAACTACGATACGGGAGGGC<br>TTACCATCTGGCCCCAGTGCTGCAATGATACCGCGAGA<br>CCCACGCTCACCGGCTCCAGATTTATCAGCAATAAACC<br>AGCCAGCCGGAAGGGCCGAGCGCAGAAGTGGTCCTGC<br>AACTTTATCCGCCTCCATCCAGTCTATTAATTGTTGCC<br>GGGAAGCTAGAGTAAGTAGTTCGCCAGTTAATAGTTT<br>GCGCAACGTTGTTGCCATTGCTACAGGCATCGTGGTGT<br>CACGCTCGTCGTTTGGTATGGCTTCATTCAGCTCCGGT<br>TCCCAACGATCAAGGCGAGTTACATGATCCCCCATGTT<br>GTGCAAAAAAGCGGTTAGCTCCTTCG	
**Oligos for ddPCR**		
**5′ end fwd**	5′-GAGCCAGAACCTCCTCGTG -3′	Fig. S8
**5′ end rev**	5′-TGGAAAGTCCCTATTGGCGTT -3′	Fig. S8
**5′ end probe**	5′-/56-FAM/CCGCCCAAC/ZEN/GACCCCCGC/3IABkFQ/-3′	Fig. S8
**3′ end fwd**	5′-CCGGACTTGTCATGATCTCC-3′	Fig. S8
**3′ end rev**	5′-CCGGGTTAAGTAAAGGTGGC-3′	Fig. S8
**3′ end probe**	5′-/56-FAM/CCGTCCACC/ZEN/TTGGACAAGTCTG/3IABkFQ/-3′	Fig. S8
**RPP30 fwd**	5′-GATTTGGACCTGCGAGCG -3′	Fig. S8
**RPP30 rev**	5′-GCGGCTGTCTCCACAAGT -3′	Fig. S8
**RPP30 probe**	5′-/5HEX/CTGACCTGA/ZEN/AGGCTCTGCGCG/3IABkFQ/-3′	Fig. S8

Table S2. Cryo-EM data collection, refinement and validation statistics

	PlaMe TPRT initiation state (EMDB-49515) (PDB 9NL2)	TaGu TPRT initiation state (EMDB- 49516) (PDB 9NL3)	PlaMe second strand nicked state (EMDB- 49517) (PDB 9NL4)
**Data collection and processing**			
Magnification	81,000	81,000	36,000
Voltage (kV)	300	300	200
Electron exposure (e–/Å^2)	50	50	50
Defocus range (μm)	-1.5 to -2.5	-1.5 to -2.5	-1.5 to -2.5
Pixel size (Å)	1.05	1.05	1.14
Symmetry imposed	*C1*	*C1*	*C1*
Initial particle images (no.)	400,309	763,427	77,001
Final particle images (no.)	30,692	18,892	32,239
Map resolution (Å)	3.2	3.3	4.6
FSC threshold	0.143	0.143	0.143
Map resolution range (Å)	3.0 to 5.0	3.0 to 5.0	4.0 to 7.0
**Refinement**			
Initial model used (PDB code)	none (generated in AlphaFold3)	none (generated in AlphaFold3)	PlaMe first strand synthesis state
Model resolution (Å)	3.3	3.3	5.7
FSC threshold	0.5	0.5	0.5
Map sharpening *B* factor (Å^2)	-50	-50	-50
Model composition			
Non-hydrogen atoms	12,160	11,899	10,770
Protein residues	1,107	1,079	1,107
Nucleic acid atoms	157	154	93
Ligands	4 (Zn), 1 (Mg), 1 (dTTP)	4 (Zn), 1 (Mg), 1 (dTTP)	4 (Zn)
B factors (Å^2)			
Protein	80.05	77.93	291.98
Nucleotide	81.15	110.45	234.45
Ligand	56.65	88.38	362.32
R.m.s. deviations			
Bond lengths (Å)	0.004	0.014	0.006
Bond angles (°)	0.881	1.100	0.831
Validation			
MolProbity score	1.91	1.92	2.55
Clashscore	7.73	8.72	26.74
Poor rotamers (%)	0.30	0.42	0.00
Ramachandran plot			
Favored (%)	92.20	93.00	86.13
Allowed (%)	7.43	6.54	13.24
Disallowed (%)	0.54	0.47	0.63
